# Size Selective Recognition of G‑Series Nerve Agents via Aryl–C–H Interactions with the Supramolecular Pinwheel and Tetracene-Based Host: A Computational Study

**DOI:** 10.1021/acsomega.6c05223

**Published:** 2026-08-25

**Authors:** Dipankar Das, Rabindranath Lo, Bishwajit Ganguly

**Affiliations:** † Computational and Simulation Unit (Marine Elements and Marine Environment Division and Centralized Instrument Facility), CSIR-Central Salt & Marine Chemicals Research Institute, Bhavnagar 364002, Gujarat, India; ‡ Academy of Scientific and Innovative Research (AcSIR), Ghaziabad 201002, Uttar Pradesh, India; § Institute of Organic Chemistry and Biochemistry, Czech Academy of Sciences, Flemingovo nám. 542/2, Prague 6 160 00, Czech Republic; ∥ CSIR-Central Glass and Ceramic Research Institute, Kolkata 700032, India

## Abstract

Organophosphorus nerve agents exert their toxicity by irreversibly inhibiting acetylcholinesterase, disrupting neurotransmission in both the central and peripheral nervous systems. Consequently, rapid and reliable recognition of these agents is crucial for timely detection and mitigation. Herein, we investigate size-selective host–guest interactions between a supramolecular pinwheel and a modeled tetracene-based host with G-series nerve agents as guests, using density functional theory (DFT) at the M06-2X-D3/6-31+G­(d) level. The computational results demonstrated that the G-series nerve agent Tabun (GA) exhibits stronger affinity and higher selectivity for encapsulation with the supramolecular pinwheel host. Similarly, Soman (GD) and Cyclosarin (GF) are effectively encapsulated within the electron-rich tetracene-based host cavity, with higher binding energies than those of the congener nerve agents. All host–guest complexes are thermodynamically favorable. Stabilization arises from multiple noncovalent interactions, including C–H···F, C–H···O hydrogen bonding, C–H···π interactions, and van der Waals forces, along with structural complementarity between host and guest. Noncovalent interactions are characterized using molecular electrostatic potential (MESP) surface topography and atoms-in-molecules (AIM) analysis, in conjunction with the reduced density gradient (RDG) method for noncovalent interaction visualization. Energy decomposition analysis further elucidates the nature and contributions of interactions governing the stability of these encapsulated complexes.

## Graphical abstract

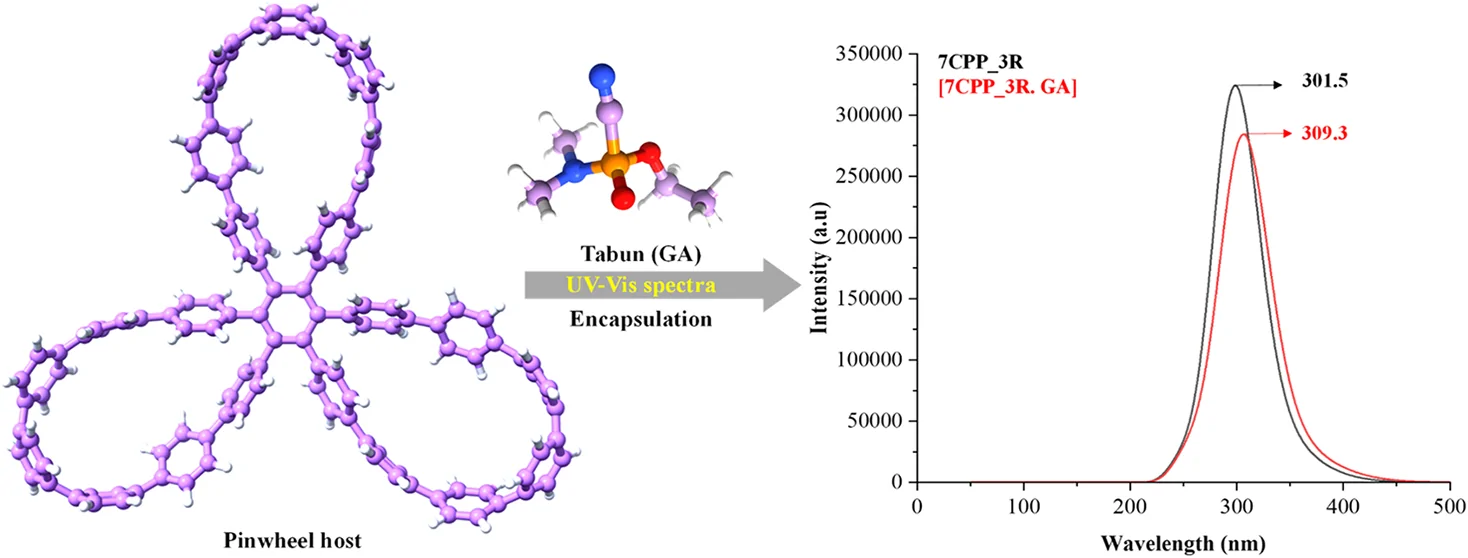

## 1 Introduction

The physiological role of the acetylcholinesterase (AChE) enzyme is to mediate neurotransmission at the cholinergic synapse in the central as well as the peripheral (CNS & PNS) nervous system.^1,2^ The pivotal role of AChE is the hydrolysis of the neurotransmitter (ACh) into acetic acid and choline at the synaptic cleft.^3,4^ This AChE-mediated hydrolysis of neurotransmitter takes place via the acylation and deacylation steps with the help of the serine residue of the catalytic triad of the AChE. As a result, a precise physiological concentration of ACh is maintained at the cholinergic synapse.^5^ Numerous natural and synthetic compounds can act as inhibitors that bind exclusively to the serine residue of the catalytic triad, leading to inhibition of the enzyme AChE. This leads to the inactivation of AChE, which completely shuts down its natural physiological functions.^6^

Such foreign molecules typically belong to the organophosphorus (OP) compound class, namely the poisonous pesticides (largely used for agricultural purposes) and the more toxic Chemical Warfare Nerve agents (CWNAs).^7^ Upon exposure to these nerve agents, the serine residue of the enzyme forms an irreversible covalent bond between the oxygen atom of the serine residue of the enzyme and the phosphorus atom of the nerve agents. As a consequence of this covalent bond formation, the enzyme is unable to hydrolyze the neurotransmitter acetylcholine at the cholinergic synapse.^8–10^ This is followed by a sudden surge of the neurotransmitter within the neuromuscular junction. As a result, overstimulation of the whole synaptic neurons finally disrupts the physiological functions of AChE, which can cause severe outcomes such as respiratory failure and even death.^10^ Therefore, owing to the toxic character of these nerve agents in real-life scenarios, they pose a serious threat to humanity.^11–14^

These OPs containing nerve agents are generally classified into two types. The G-type (German type) includes Sarin (GB), Soman (GD), Tabun (GA), and Cyclosarin (GF). The venom type V-series nerve agents contain VX, Russian VX, and VR.^7,15^ Upon exposure to these toxic nerve agents, it is most important to detect these nerve agents in real time for timely diagnosis and intervention. There is a limited number of detection methods that have been cited in the literature, such as instrumental techniques (HPLC, gas chromatography, and NMR methods),^16–19^ as well as covalent approaches,^20^ and more recently, supramolecular recognition strategies.^15^ Instrumental techniques are generally costly, whereas sensor-based covalent approaches are relatively inexpensive and allow rapid detection. However, covalent sensing approaches involve bond formation between the analyte and the sensor, which can form new compounds, leading to false-positive responses.^15^ Consequently, such approaches may lead to false positives and reduced reliability of these nerve agents.

Recently, supramolecular strategies have been developed in which supramolecular hosts encapsulate nerve agents within their cavities via noncovalent interactions. Such recognition processes are completely reversible in nature.^21,22^ Owing to these properties, supramolecular hosts have attracted considerable attention from several research groups. Numerous supramolecular hosts have been reported in the literature for sensing and recognizing various guest molecules.^23^ Among them, the tunable nature of the [n]­cycloparaphenylene supramolecular hosts has made them an emerging class of molecular hosts in recent years. Previously, we have also employed supramolecular approaches in which the host^6^ cycloparaphenylene encapsulated the specific G-series nerve agents Tabun.^22^ Due to their complementary nature, these nerve agents are effectively encapsulated within the core cavity of the host molecule. In view of these evolving supramolecular recognition strategies, we introduce the Pinwheel supramolecular host for the recognition of a variety of G-series nerve agents. In addition, recent advances in cycloparaphenylene (CPP)-based nanohoop architectures have demonstrated that precisely engineered π-conjugated hosts can achieve highly selective guest encapsulation through shape complementarity and concave–convex π–π interactions. Structurally defined double-nanohoops reported in the literature are highlighted to control cavity size, rigidity, and multivalent interactions that govern molecular recognition and higher-order complexation with fullerenes.^24,25^ CPP-based frameworks incorporating two or more nanohoop rings create multiple, spatially separated binding cavities. As a result, these systems are well-suited for forming 1:*n* ternary host–guest complexes, in which a single host can bind several guest molecules at the same time.^25,26^ Compared to single nanohoop systems, multinanohoop architectures offer several advantages: they enable stepwise complexation, where guests bind sequentially to different nanohoops, often with varying binding constants, and heterogeneous guest binding, where distinct nanohoops can selectively accommodate different types of guest molecules. These characteristics are particularly important in supramolecular chemistry, especially for the design of porous materials with controlled uptake of multiple species and chemical sensors capable of selective, multianalyte detection. Inspired by these principles, we extended this nanohoop design strategy toward the selective recognition of organophosphorus nerve agents. Unlike spherical fullerenes, G-series agents possess anisotropic geometries and polar –P=O functionalities that demand directional noncovalent interactions. Accordingly, we have modeled supramolecular anthracene-based host systems with a cavity tailored to the size and shape of these agents. The presence of positioned interaction sites enables hydrogen bonding and C–H···π surface interactions with the phosphoryl oxygen and alkyl groups, thereby enabling the selective detection of toxic organophosphorus compounds (Figure 1).

**Figure 1:**
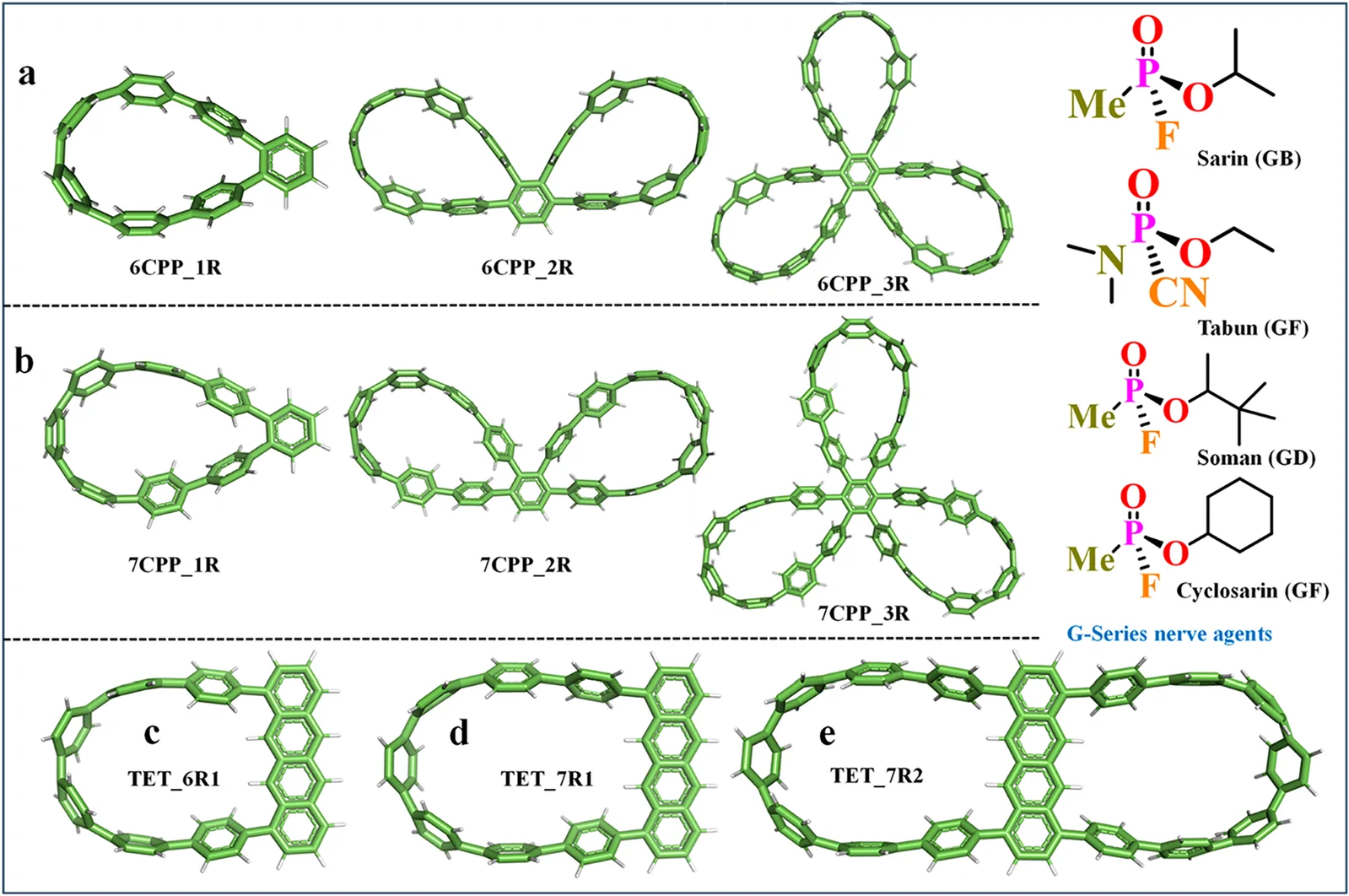
Geometries of the constituent fragments of the pinwheel supramolecular host and the tetracene-based supramolecular host. (a) six-membered, (b) seven-membered pinwheel host, and (c) six-membered tetracene-based host, (d) seven-membered tetracene-based host, and (e) Seven-membered tetracene-based double-hooped supramolecular host. Extremely right chemical structure of G-series nerve agents.

The hosts investigated in this study were classified according to their synthetic accessibility. (a) Synthesized and structurally characterized hosts: None of the hosts investigated in this study were used directly from the experimentally reported pinwheel host. (b) Modified versions of synthesized hosts: The experimentally determined parent pinwheel host reported by Clayton et al. (CCDC 2334370),^27^ was used for the recognition study. Due to the relatively large cavity size of the experimentally reported parent pinwheel host, which contains nine phenylene units per pore, it is less suitable for efficient encapsulation of G-series nerve agents. Therefore, the experimentally determined parent pinwheel structure was rationally modified by reducing the nanohoop size while preserving the overall pinwheel framework. This strategy led to the design of **6CPP_1R**, **7CPP_1R**, **7CPP_2R**, and **7CPP_3R** hosts, containing six or seven phenylene units in each pore, which provide more appropriate cavity dimensions for effective encapsulation and enhanced recognition of G-series nerve agents. Consequently, these hosts represent the computationally modified hosts of an experimentally characterized structure. In addition, (c) Computationally designed hosts: The tetracene-based hosts (**TET_7R1** and **TET_7R2)** were computationally designed to explore the influence of π-conjugation and the cavity for the supramolecular recognition study. However, these design hosts have not yet been experimentally synthesized. Nevertheless, considering the substantial progress in cycloparaphenylene and nanohoop chemistry,^25^ these hosts may be synthetically accessible through suitably adapted macrocyclization and cross-coupling strategies,^28,29^ although their experimental realization remains a subject for future investigation.

The size and shape of the G-series nerve agents are complementary to the six- and seven-membered pinwheel supramolecular host. Therefore, in this study, we selected six- and seven-membered pinwheel hosts. To gain deeper insights into the binding interactions and stability of these host–guest complexes, we have employed a range of computational approaches, including molecular electrostatic potential (MESP) surface topography and atoms-in-molecules (AIM) analysis, in conjunction with the reduced density gradient (RDG) method for noncovalent interaction (NCI) visualization, energy decomposition analysis (EDA), and time-dependent density functional theory (TD-DFT).

## 2 Computational Methodology

Initially, the supramolecular pinwheel hosts were obtained from the CCDC (CCDC ID: 2334370) crystallographic database.^27^ Subsequently, all fluorine atoms were deleted and replaced with hydrogen atoms. Afterward, we designed supramolecular pinwheel hosts with an appropriate cavity size tailored to G-series nerve agents, such as **6CPP_3R** and **7CPP_3R**. The hydrogen-substituted pinwheel host examined in this study was previously synthesized,^27^ but its crystallographic structure remains unreported. We therefore used the experimentally characterized fluorinated analogue as the template for our computational models. Furthermore, we modeled tetracene-based supramolecular hosts **TET_6R2** and **TET_7R2**. Thereafter, conformational searches of all supramolecular hosts and the G-series nerve agents were performed employing the MMFF force field^30^ implemented in the Spartan08 software package.^31^ The lowest energy conformers of these nerve agents and hosts were studied for further DFT studies. All individual molecules and complex systems were optimized using the M06-2X functional^32^ developed by Truhlar and co-workers,^33^ integrated with Grimme’s (D3) dispersion corrections.^34^ Grimme’s dispersion corrections are widely applied for the investigation of noncovalent interactions.^34–39^ The 6–31+G­(d) basis set was used for the complex optimization. Additionally, basis set superposition error (BSSE) was corrected using the counterpoise (CP) method^40^ to obtain more accurate interaction energies. The frequency calculations were performed for all the studied systems to confirm the true energy minima. Standard rigid rotor–harmonic oscillator (RRHO) approximations were used to compute the Δ*G* at the M06-2X level. Please note that quasi-RRHO models (e.g., Grimme’s treatment) generally refine entropy estimates for low-frequency vibrations in flexible hosts and guests.^35^ However, because all systems were evaluated under an identical protocol, systematic errors inherent to the RRHO framework are expected to cancel out. The derived relative binding affinities, selectivity trends, and qualitative insights therefore remain dependable, despite potential minor deviations in absolute Δ*G* and *K*_a_ values. All theoretical calculations were performed using the Gaussian 09 software.^41^ To verify the binding energy of these complexes, single-point energies were calculated with M06-2X-D3/6-311++G­(d,p) level of theory in the gas phase. Furthermore, to investigate the influence of solvent effects on the nerve agent-encapsulated complex system, additional theoretical calculations were carried out at the M06-2X-D3/6-31+G­(d) level of theory using the polarizable continuum model (PCM) solvent model in dichloromethane (DCM = 8.39).^42^ All the encapsulated complex system binding energies (Δ*E*) were calculated using the following eq ([Disp-formula eq1])

1 $$\Delta E_{\mathrm{binding}\;\mathrm{energy}}=E_{\mathrm{complex}}-\left(E_{\mathrm{host}}+E_{\mathrm{nerve}\;\mathrm{agents}}\right)$$

The Δ*E*_binding energy_ is defined as the difference between the energy of the encapsulated host–guest complex (*E*_complex_) and the energies of the isolated host (*E*_host_) and G-series nerve agents (*E*_nerve agents_).

Similarly, the binding free energies of G-series nerve agents encapsulated in the complex with the host system were also computed using the following eq ([Disp-formula eq2]) as

2 $$\Delta G_{\mathrm{binding}\;\mathrm{energy}}=G_{\mathrm{complex}}-\left(G_{\mathrm{host}}+G_{\mathrm{nerve}\;\mathrm{agents}}\right)$$

where the Δ*G*_binding energy_ signifies the total binding free energy of the encapsulated complex systems, whereas the *G*_host_ and *G*_nerve agents_ are the separated free energies of the supramolecular host and individual nerve agents.

The equilibrium association constant (*K*_a_) was also calculated by using the binding free energies of these nerve agents encapsulated complex systems, applying the given eq ([Disp-formula eq3]) at 298 K.

3 $$K_{a}=e^{\left(-\Delta G/\mathrm{RT}\right)}$$

Where, Δ*G* is the binding free energy, temperature *T*, and *R* is the gas constant.

To map the molecular surface of the supramolecular host molecules and the G-series nerve agents, we also performed MESP using the Multiwfn 3.6 software^43^ employing the following eq ([Disp-formula eq4]).

4 $$V\left(r\right)=\sum_{A}\frac{Z_{A}}{\left|R_{A}-r\right|}-\int \frac{\rho \left(r^{\prime }\right)}{\left\lfloor r^{\prime }-r\right\rfloor }{dr}^{\prime }$$

In eq ([Disp-formula eq3]), ρ­(*r*′) refers to the electron density, where the *V*(r) is the potential at a point r relative to the nuclei and electrons of the molecules, and *Z*_A_ denotes the charge of the nucleus A situated at *R*_A_.

In order to examine the optoelectronic properties of the G-series nerve agent encapsulated complex system with the supramolecular host, time–dependent density functional theory has been employed.^44,45^ The ground-state geometry optimization of these complex systems was carried out at the M06-2X-D3/6-31+G­(d) level of theory in the gas phase. The optimized ground-state geometries of the studied nerve-agent-encapsulated complex systems have been used to compute the vertical excitation energies (S_0_ → S_1_) and the oscillatory factor (*f*) in this TD-DFT analysis. In this work, for these studied complex systems, the 50 singlet excited states (*n* states = 50) were considered. For each excited state, the vertical TD-DFT calculation basically yielded transition energies, associated oscillator strengths, and molecular orbital composition. The same TD-DFT method was applied to investigate the emission spectra of these G-series nerve agents encapsulated complex systems by optimizing the first singlet excited state (*S*_1_). To confirm that the excited structure corresponds to a true minimum, we performed frequency analysis, and no imaginary frequencies were present in the computed encapsulated complex systems. The computed TD-DFT results for these G-series nerve agent-encapsulated complex systems provide valuable optoelectronic properties for the recognition of G-series nerve agents.

Furthermore, to elucidate the nature of the fundamental weak noncovalent interactions of the G-series nerve agents encapsulated complex systems with the supramolecular host, symmetry-adapted perturbation theory (SAPT) calculations were performed using the PSI4 package.^46^ Considering computational limitations imposed by the size of the G-series nerve agents encapsulated in the studied complex systems, the simplified SAPT0 ^47^ level of theory was adopted in conjunction with the def2-SVP basis set. The following interaction terms are associated with SAPT0 in eq ([Disp-formula eq5]) as follows

5 $$E^{\mathrm{SAPT}0}=E_{\mathrm{elect}}+E_{\mathrm{exch}}+E_{\mathrm{ind}}+E_{\mathrm{disp}}$$

The *E*_elect_, *E*_exch_, *E*_ind_, and *E*_disp_ signify the contributions of electrostatic, exchange-repulsion, induction, and dispersion interactions of the G-series nerve agent-encapsulated complex systems, respectively. The electrostatic energy terms (*E*_elect_) originate from the Coulombic interactions between the charge densities of the individual supramolecular host and the nerve agent monomers. The exchange-repulsion energy contribution (*E*_exch_) comes from the interaction between the overlapping supramolecular host and the nerve agent monomer wave functions. The induction energy contribution term (*E*_ind_) represents the polarization effects induced by the individual monomers electric fields, along with the contribution of intermolecular charge transfer. Similarly, the dispersion energy term (*E*_disp_) arises from the electron–electron correlation between the fluctuating charge densities of the supramolecular host and the nerve agent’s monomers. In general, the electrostatic, induction, and dispersion energy terms play a crucial role in the encapsulation of G-series nerve agents within the supramolecular host and in stabilizing the complex systems. Therefore, we determined the percentage contributions of these individual energy terms of these encapsulated complex systems as follows in eq ([Disp-formula eq6]).

6 $$percentage\;of\;contribution=\left(\frac{E_{energy\;term}}{E_{elec}+E_{ind}+E_{dis}}\right)\times 100\%$$

Where the *E*_energy term_ represents the individual electrostatic, induction, and dispersion interaction energies.

## 3 Results and Discussion

All calculations of these encapsulated G-series nerve agents with respective pinwheel host and modeled host have been carried out using the M06-2X-D3/6-31+G­(d) level of theory in the gas phase. To investigate the binding sites of the host and guest molecules, molecular electrostatic potential (MESP) surface maps were constructed. The MESP surface analysis demonstrated that the blue regions define the corresponding positive potential, whereas the red regions signify the negative potentials of these G-series nerve agents and host molecules (Figure 2). For the supramolecular pinwheel and anthracene-based designed hosts, the negative electrostatic potential is predominantly distributed within the core cavity of these hosts, as well as the outer periphery near the phenyl groups. Additionally, the positive potential region is localized along the rim of these two host molecules. Conversely, the MESP surface analysis of the G-series nerve agents showed that the maximum negative potential was located on the oxygen atom of these nerve agents, except for Tabun, where the −CN group also displayed an electron-rich site along with the oxygen atoms. During the encapsulation of the G-series nerve agents within the supramolecular pinwheel host, the positively charged region interacts with the negatively charged sites, resulting in well-defined directional interactions between the host and guest molecules.

**Figure 2:**
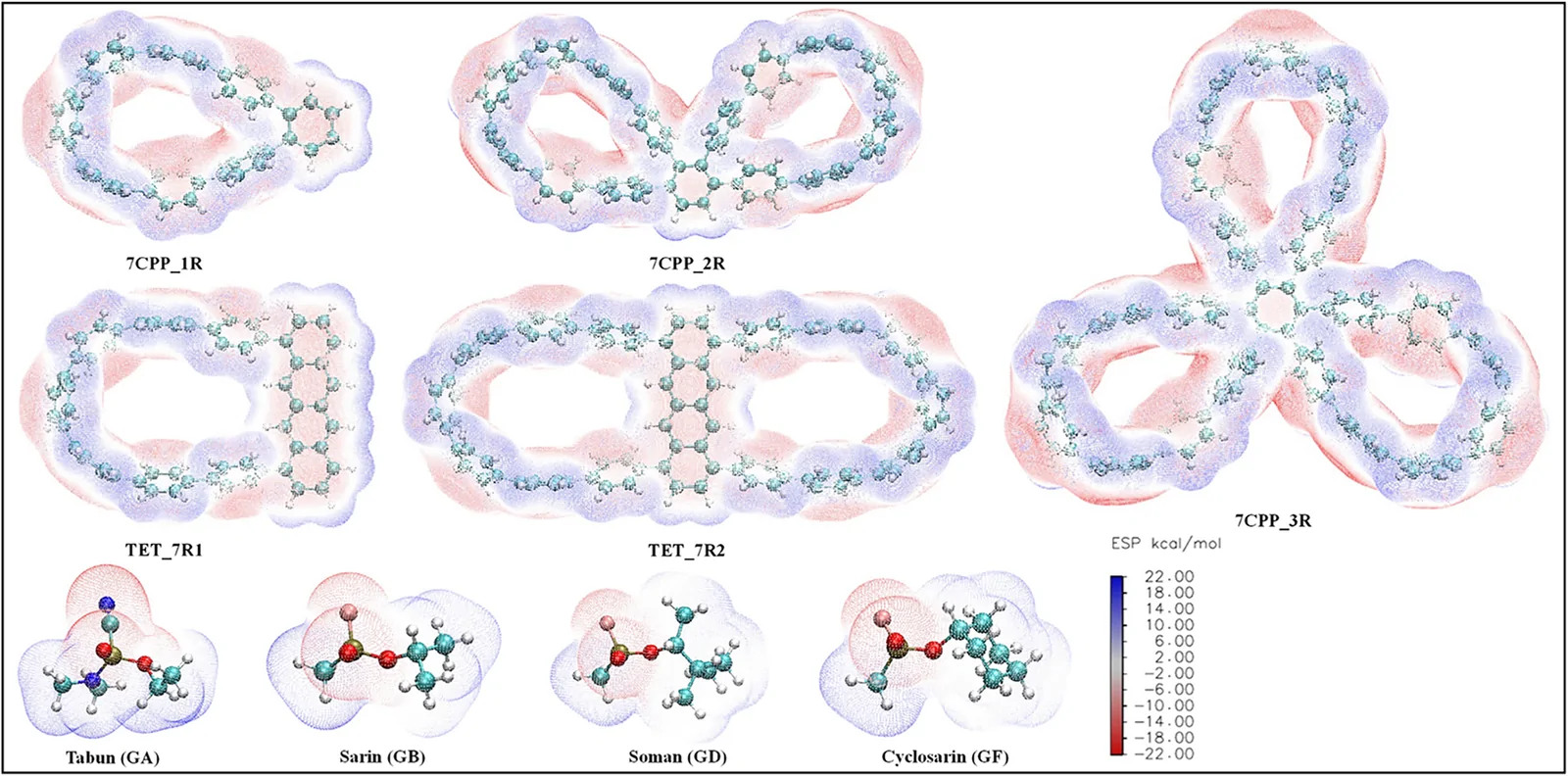
MESP surface of the supramolecular pinwheel and tetracene-based host and G-series nerve agents employing the M06-2X-D3/6-31+G­(d) level of theory in the gas phase.

Initially, we have calculated the binding energies of the G-series nerve agent encapsulated in the complex with the fragment of the pinwheel, i.e., the six-membered **6CPP_1R** supramolecular host. Similarly, we have investigated the binding energies with the seven-membered fragment of the pinwheel host (**7CPP_1R**) (Figure 3). The observed results showed that the seven-membered pinwheel fragment supramolecular host encapsulates the G-series nerve agent more strongly within its cavity of the host (Table 1), compared to the six-membered fragment pinwheel host (Table S1). The complementary size of the G-series nerve agents with the seven-membered **7CPP_1R** (cavity volume 665.9 Å^3^) pinwheel host suggests that more efficient host–guest encapsulation occurs (Figure S1). In contrast, the six-membered **6CPP_1R** pinwheel host possesses a relatively smaller and more compact cavity (**6CPP_1R**: cavity volume 582.8 Å^3^) compared to the molecular dimensions of G-series nerve agents (Figure S1). Consequently, this geometrically restricted cavity is expected to exhibit a relatively lower binding affinity for the G-series nerve agents than the larger host. Therefore, in the present study, we considered the seven-membered fragment of the pinwheel supramolecular host for further investigation. The computed binding free energies revealed that the supramolecular host encapsulated the G-series nerve agents exhibited the following order: **[7CPP_1R. GA] > [7CPP_1R. GD] > [7CPP_1R. GF] > [7CPP_1R. GB]** (Table 1). The G-series nerve agent Tabun is encapsulated within the supramolecular host cavity with the highest binding free energy compared to the rest of the G-series nerve agents. Furthermore, we have calculated the BSSE correction energies of these complex systems. The calculated BSSE-corrected energies showed a similar trend to that observed in binding energies (Table 1), where Tabun exhibited the highest binding energy. Additional calculations were performed in dichloromethane (DCM) solvent medium to account for the solvent effects in the complexation between the supramolecular host and the G-series nerve agents. The computed single-point calculation exhibited a similar trend to that obtained in the gas-phase study (Table S2).

**Figure 3:**
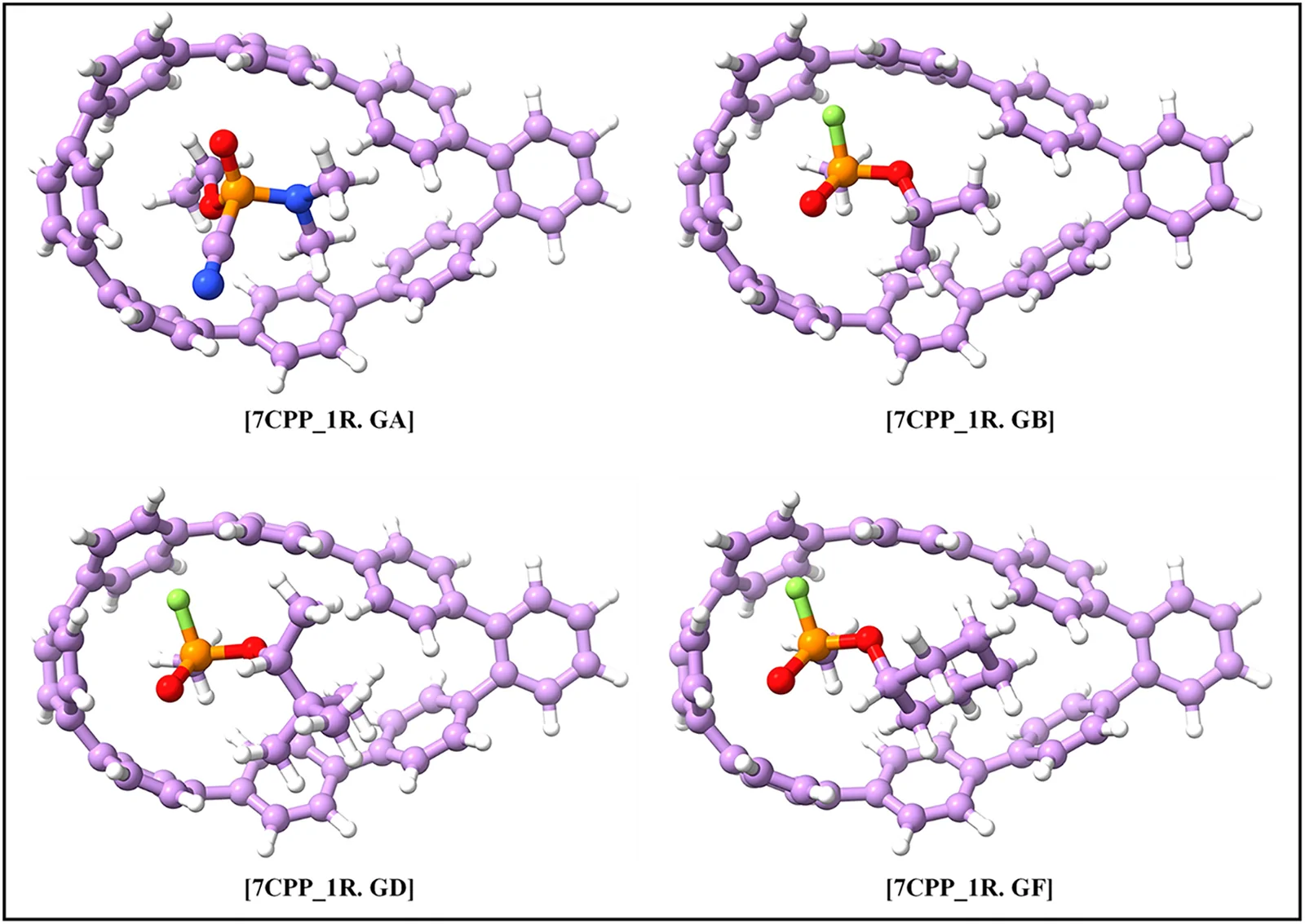
Optimized encapsulated complex geometries of the host **7CPP_1R** with G-series nerve agents, calculated at the M06-2X-D3/6-31+G­(d) level of theory in the gas phase. In the host, carbons and hydrogens are depicted in magenta and white, whereas in nerve agents, green, red, orange, magenta, and white colors signify the fluorine, oxygen, phosphorus, carbon, and hydrogen atoms, respectively, shown in a ball-and-stick model.

**Table 1:** Computed Binding Energies, the Counterpoise-Corrected Interaction Energies (Δ*E*_CP_), Binding Free Energies, and the Equilibrium Association Constants (*K*_a_) of the G-Series Nerve Agents Encapsulated within the Supramolecular Pinwheel Fragment Host **7CPP_1R** Complex Systems Using M06-2X-D3/6-31+G­(d) Level of Theory in the Gas Phase^a^

system	Δ*E*	*ΔE*_CP_	Δ*G*	*K*_a_ (M^–1^)
**[7CPP_1R. GA]**	–26.4	–25.8	–9.1	4.7 × 10^6^
**[7CPP_1R. GD]**	–22.6	–21.2	–5.0	4.6 × 10^3^
**[7CPP_1R. GF]**	–21.6	–21.9	–4.8	3.3 × 10^3^
**[7CPP_1R. GB]**	–19.8	–19.2	–4.1	1.0 × 10^3^

a All energies are given in kcal/mol.

In addition, for the thermodynamic preference of these nerve agents toward the pinwheel host, we calculated the equilibrium association constant (*K*_a_) applying the standard thermodynamic eq 3. The calculated (*K*_a_) values indicate that the Tabun encapsulated complex exhibits the strongest gas-phase association, with an association constant of (4.7 × 10^6^ M^–1^), whereas the rest of the encapsulated complex systems show considerably lower values (Table 1). Apart from that, for **[7CPP_1R. GA]** and **[7CPP_1R. GD]** encapsulated systems, the *K*_a_ value has been calculated in DCM solvent as a representative case. In this solvent, the observed association constant *K*_a_ values are 4.6 × 10^3^ and 5.41 M^–1^, respectively, which are lower than corresponding values obtained in the gas phase. Notably, the difference in the (*K*_a_) values of GA and GD exceeds 3 orders of magnitude ((>10^3^)), indicating a remarkable difference in their binding affinities. Thus, the **7CPP_1R** host displays a pronounced thermodynamic preference for nerve agent Tabun (GA) over Soman (GD) even in solution. The relatively large gas-phase (*K*_a_) values arise from the absence of solvent screening and solvent–solute competition. Under gas-phase conditions, hydrogen-bonding, electrostatic, dispersion, and CH···π interactions can therefore contribute more directly to complex stabilization. The DCM values yield a more realistic model of binding in low-polarity environments; in more polar media, association constants decrease significantly owing to strong solvation and competing host/guest–solvent interactions.

The binding free energy of the encapsulated complex system demonstrated that the Tabun nerve agent efficiently and most effectively encapsulated within the supramolecular pinwheel host cavity. The enhanced binding affinity of the Tabun (GA) originates mainly from its suitably orientated functional groups, where the linearly oriented ethyl fragment enables the favorable noncovalent interactions (C–H···π interactions) within the electron-rich cavity of the supramolecular pinwheel host, along with other H-bonding interactions (H-bonding interactions with N, F, and O atoms of the nerve agent with periphery hydrogen atoms of the host molecule). This favorable molecular orientation is disrupted when the relatively bulkier G-series nerve agent is encapsulated within the pinwheel **7CPP_1R** host cavity. The gas phase single-point energies were calculated at the M06-2X-D3/6-311++G­(d,p) level of theory, and followed the same pattern as those computed employing the M06-2X-D3/6-31+G­(d) level of theory (Table S2). However, to gain further insight into the magnitude of C–H···π interactions in these G-series nerve agents encapsulation complex systems, an additional calculation was performed at the same level of theory using the four small molecular systems (cyclohexanol, isobutene, isopropanol, and ethanol) with the **6CPP_1R** host. In practical applications, additional interferents such as water and the organophosphorus pesticide parathion have been studied with the **6CPP_1R** host. The computed results revealed that the G-series nerve agents exhibited significantly higher binding affinities than the smaller molecules, including the pesticide parathion reference molecules (Figure S2 and Table S3).

The noncovalent interactions, such as van der Waals interactions, C–H···π interactions, and H-bonding interactions, play a crucial role in both the encapsulation of analytes within supramolecular hosts and the selective recognition of nerve agents.^22,48,49^ In our present study, the strong affinity of the G-series nerve agent Tabun toward encapsulation within the pinwheel host cavity is significantly stabilized by the CH···π noncovalent interactions. Such CH···π and H-bonding interactions are also evident in various supramolecular systems involving cavitands with nerve agent simulant DMMP and Calix[4]­pyrrole hosts with different ligands.^50^ They are particularly significant in the Chrysenylene[4]­CC complex with a bowl-shaped guest, where CH···π interactions serve as a dominant driving force for the complex stability.^51^

In DFT-based studies, the molecular characteristics of supramolecular host–guest interactions are elucidated using a set of quantum descriptors, including atoms-in-molecules (AIM) analysis, noncovalent interaction (NCI) analysis, and the MESP, as described in an earlier section. In this context, Bader’s AIM methodology has been widely applied to investigate H-bonding, CH···π, and other weak noncovalent interactions.^52^ To examine such interaction patterns between the supramolecular pinwheel host and G-series nerve agents, AIM analysis was performed at the M06-2X-D3/6-31+G­(d) level of theory in the gas phase employing the Multiwfn 3.6 program. These interactions are characterized by evaluating the electron density at the bond critical point (3, −1), its Laplacian, and associated local energy densities (G, V, and H), which collectively indicate the strength and degree of covalency. By evaluating the values and sign of these topological parameters, Bader’s theory distinguishes the nature of the chemical bonds according to the following criteria. In the case of closed-shell interactions, *V*(r)/*G*(r) < 1, while in shared-shell interactions, *V*(r)/*G*(r) > 2. Values between 1 and 2 indicate intermediate interactions (Figure S3 and Table S4).

Further, the noncovalent interaction (NCI) provides a real-space graphical representation of the noncovalent interactions and is capable of distinguishing between hydrogen bonding, van der Waals, and steric repulsion interactions of the encapsulated complexed systems.^53–55^ Therefore, to investigate the characteristics of noncovalent interactions between these guests, G-series nerve agent encapsulated with the supramolecular pinwheel host molecules at the M06-2X-D3/6-31+G­(d) level of theory in the gaseous phase, we employed the NCI-RGD using the Multiwfn software, which relies on the electron density redistribution analysis. According to this NCI methodology, the nature and the strength of the noncovalent interactions represented by the RDG isosurfaces are interpreted using the parameter (sin λ_2_)­ρ, which is defined by the electron density (ρ) and multiplied by the sign of the second eigenvalue (λ_2_) of the electron density Hessian matrix. The parameter (sin λ_2_)­ρ serves as a useful indicator of these interactions, where (sin λ_2_)­ρ > 0 represents repulsive interactions, (sin λ_2_) ρ < 0 represents hydrogen bonding interactions, and (sin λ_2_)­ρ ≈ 0 signifies attractive van der Waals interactions. The RDG isosurfaces for the G-series nerve agent-encapsulated complex systems are shown in Figure 4, along with the corresponding 2D plots. Furthermore, in a 2D representation, the spike appearing at ρ ≈ 0 indicates the confirmed presence of CH···π and other van der Waals interactions within the encapsulated complex systems. In contrast, the appearance of the positive RGD region near ρ = 0.002 au is attributed to the bulky hydrophobic moieties of the G-series nerve agents, reflecting the steric repulsion within the supramolecular host cavity. Overall, the NCI analyses reveal the presence of diverse weak interactions in these nerve agent-encapsulated complex systems, with CH···π and van der Waals interactions predominantly governing the stabilization of the encapsulation complexes.

**Figure 4:**
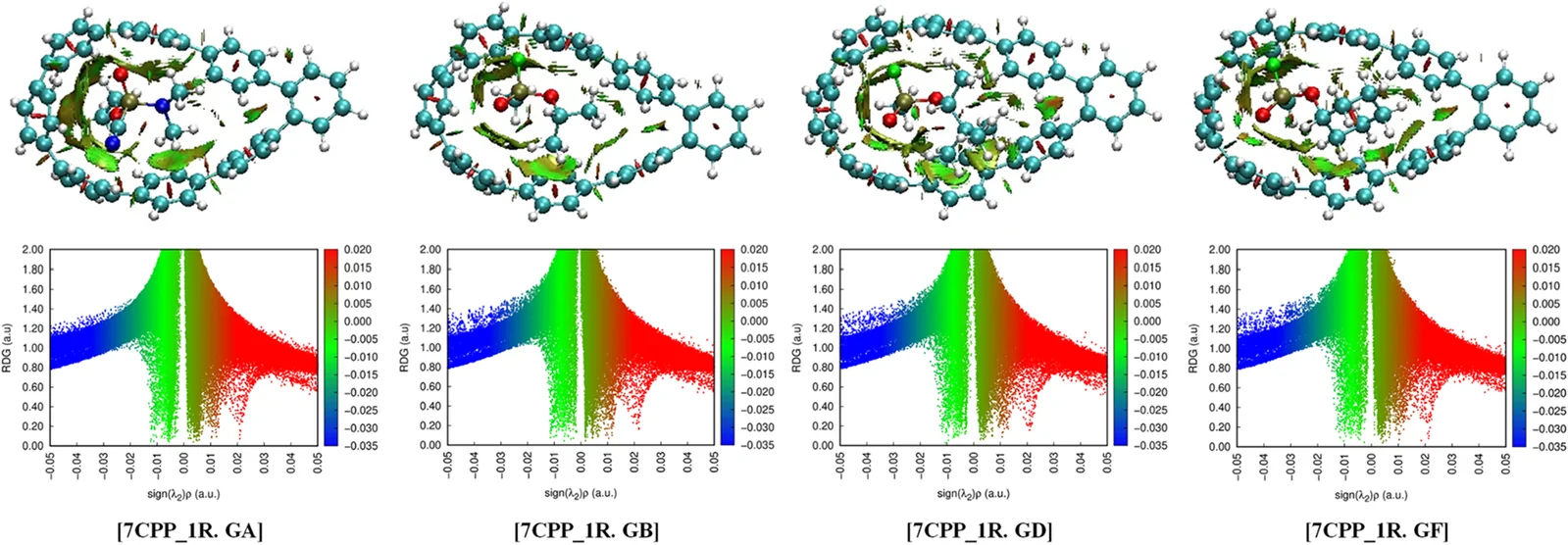
Reduced density gradient (RDG) isosurface analysis and NCI scatter diagram visualized the noncovalent interactions of these G-series nerve agent encapsulated complex systems with the supramolecular pinwheel fragment host.

In order to recognize these nerve agents by supramolecular host molecules, experimental identification is essential for real-world applications. Therefore, to explore the optoelectronic characteristics of these encapsulation complex systems, UV–vis absorption spectra (λ_max_) were investigated employing the time-dependent density functional theory at the M06-2X-D3/6-31+G­(d) level of theory in the gas phase (Figure 5). For the pristine supramolecular pinwheel **7CPP_1R** host, the computed UV–vis absorption spectrum shows a peak at 291.9 nm (*f* = 1.7643) corresponding to the H → L + 1 transition (Table S5 and Figure S4) with high intensity. Upon encapsulation of G-series nerve agent Tabun within the host, the prominent absorption peak of **7CPP_1R** host decreases in intensity and exhibits a red-shift to 298.6 nm (*f* = 1.5734), corresponding to the H → L + 1 transition, as shown in Figure 5. In contrast to Tabun, the remaining G-series nerve agent encapsulation complexes exhibit relatively smaller spectral shifts (Table S5).

**Figure 5:**
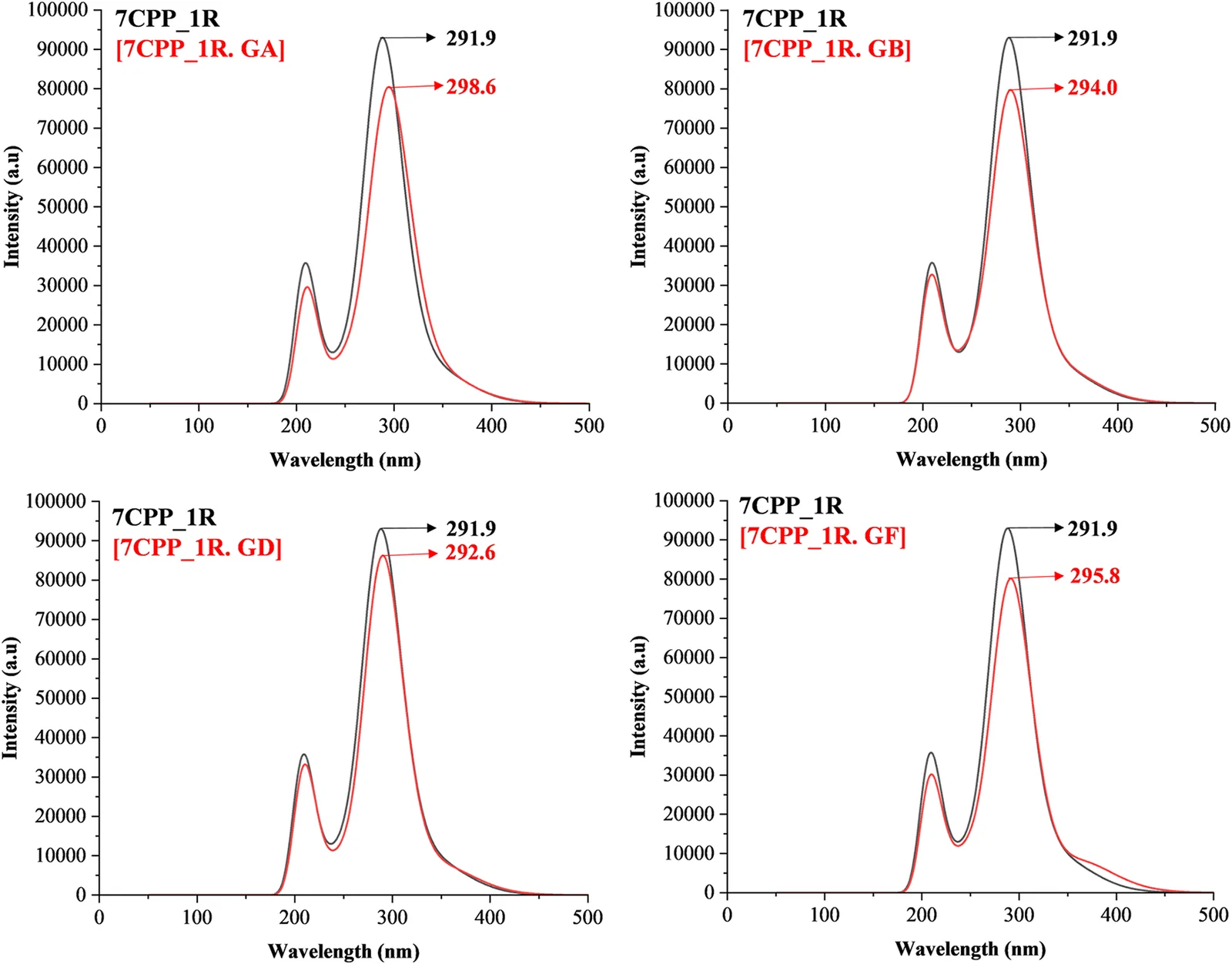
UV–vis spectra of the G-series nerve agents encapsulated complex systems with the supramolecular pinwheel fragment **7CPP_1R** host, calculated at the M06-2X-D3/6-31+G­(d) level of theory in the gas phase.

Furthermore, the characteristic emission spectra of these studied G-series nerve agents encapsulated in complex systems with the host molecule were investigated at the M06-2X-D3/6-31+G­(d) level of theory in the gas phase. The computed emission wavelengths are shown in Figure 6. The emission spectra (λ_emi_) of the pinwheel fragment **7CPP_1R** host exhibit peaks at 335.8 and 506.2 nm (*f* = 1.856 and 0.2927), primarily attributed to the H → L + 1 transition (Figure 6). Upon formation of an encapsulation complex between the G-series nerve agent Tabun and the pinwheel host, the emission spectrum (λ_emi_) of the complex shows noticeable quenching compared to that of the isolated host. For the Tabun embedded complex system, pronounced red-shifted emission peaks are observed at 343.6 nm and a slightly blue-shifted 505.6 nm, corresponding to the H → L + 1 and H → L transitions (Figure 6). In contrast, other G-series nerve agents exhibit comparatively smaller emission spectral shifts (Table S6 and Figure S5). Overall, upon complexation with the G-series nerve agents, the encapsulated complex systems exhibit dominant emission peaks in the range of approximately 335–344 nm. However, a unique diagnostic signature was observed for Tabun encapsulated within the supramolecular host cavity, highlighting its potential application for selective recognition of G-series nerve agents.

**Figure 6:**
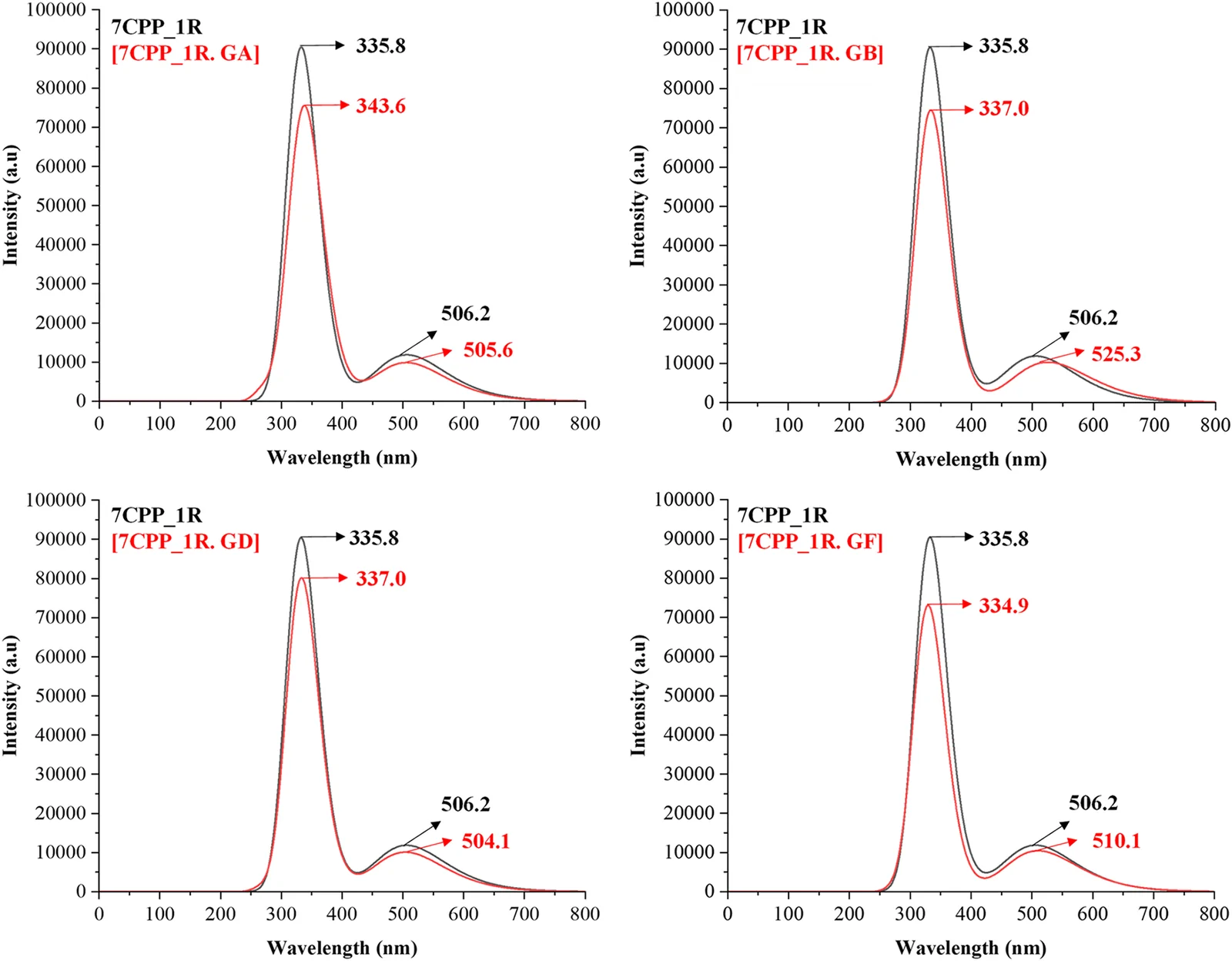
Emission spectra of the G-series nerve agents encapsulated complex system with supramolecular pinwheel fragment **7CPP_1R** host calculated at the M06-2X-D3/6-31+G­(d) level of theory in the gas phase.

Among the studied encapsulation complexes between the G-series nerve agents and the supramolecular pinwheel fragment **7CPP_1R** host, Tabun (GA) is more efficiently encapsulated within the cavity of the host, exhibiting the highest binding free energy, as well as the largest redshifts in both the UV–vis absorption and emission spectra. Therefore, for further investigation with the supramolecular **7CPP_2R** and the complete pinwheel **7CPP_3R** hosts, only Tabun (GA) was considered as the guest molecule for recognition. The binding free energies of the Tabun embedded complexes and their optimized geometries are presented in Table 2 and Figure 7, respectively.

**Figure 7:**
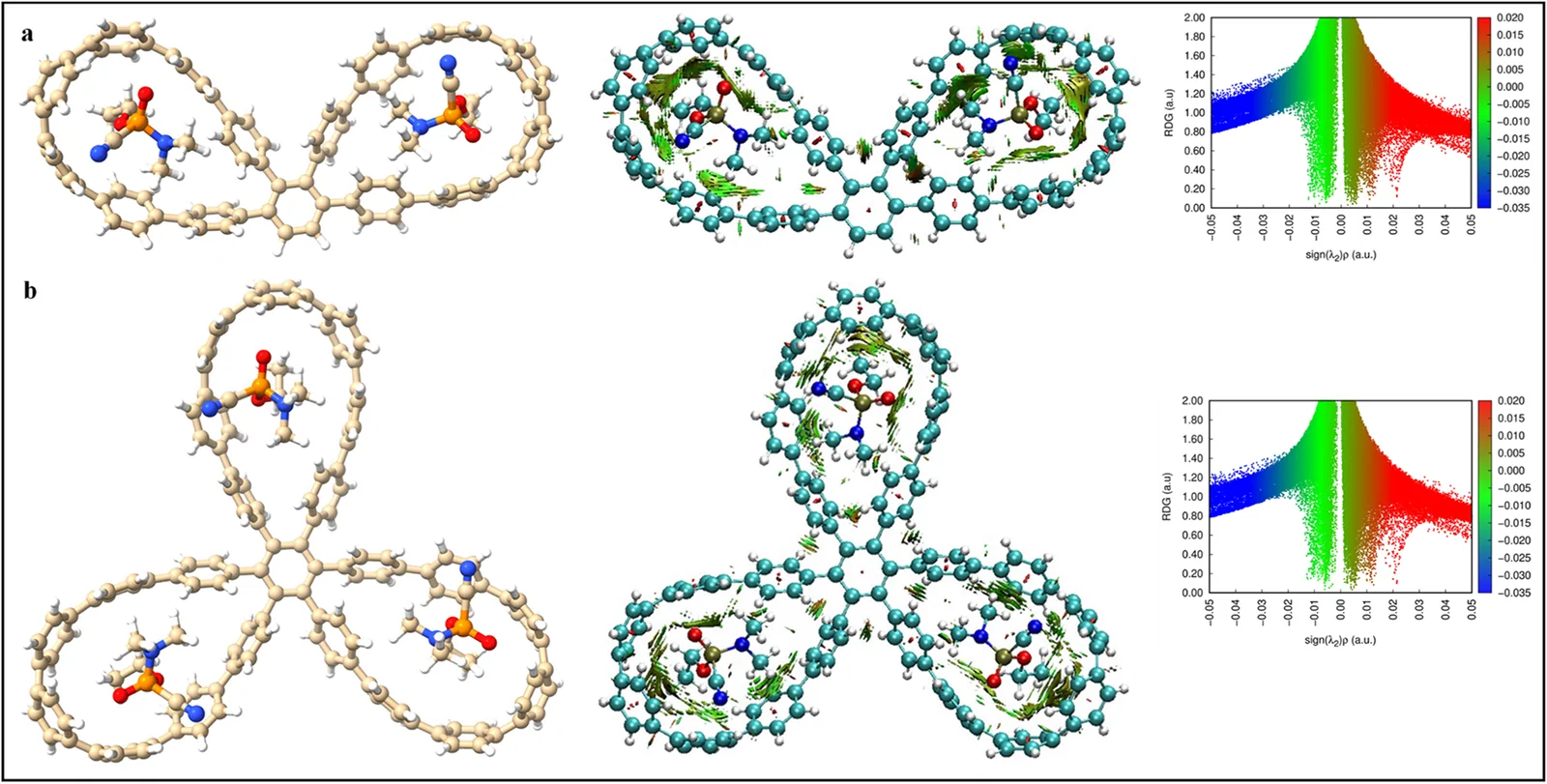
Optimized encapsulated complex structures of the **7CPP_2R** and complete pinwheel **7CPP_3R** hosts with the G-series nerve agent Tabun, employing the M06-2X-D3/6-31+G­(d) level of theory in the gas phase. (a) **[7CPP_2R. GA]** complex system and (b) **[7CPP_3R. GA]** complex system. In the host, carbons and hydrogens are depicted in brown and white, whereas in nerve agents, green, red, orange, brown, and white colors signify the fluorine, oxygen, phosphorus, carbon, and hydrogen atoms, respectively, as shown in a ball-and-stick model. The reduced density gradient (RDG) isosurface analysis and NCI scatter diagram visualized the noncovalent interactions of these G-series nerve agent encapsulated complex systems with the supramolecular pinwheel hosts.

**Table 2:** Computed Binding Energies, the Counterpoise-Corrected Interaction Energies (Δ*E*_CP_), Binding Free Energies, and the Equilibrium Association Constants (*K*_a_) of the G-Series Nerve Agents Encapsulated within the Supramolecular Pinwheel Fragment Host **7CPP_2R** and Complete Pinwheel **7CPP_3R** Host Complex Systems Using the M06-2X-D3/6-31+G­(d) Level of Theory in the Gas Phase^a^

system	Δ*E*	Δ*E*_CP_	Δ*G*	*K*_a_ (M^–1^)
**[7CPP_2R. GA]**	–52.0 (−26.0)	–51.7	–20.2 (−10.1)	**6.5 × 10** ^**14**^
**[7CPP_3R. GA]**	–77.8 (−25.9)	–77.7	–28.5 (−9.5)	**8.0 × 10** ^**20**^

a The average binding energies are given in parentheses. All energies are given in kcal/mol.

The optoelectronic properties of these complex systems were further investigated using TD-DFT at the M06-2X-D3/6-31+G­(d) level of theory in the gas phase. The UV–vis absorption and emission spectra of this Tabun-encapsulated complex system are shown in Figure 8, Supporting Information Figures S4, S5, and Tables S5, S6.

**Figure 8:**
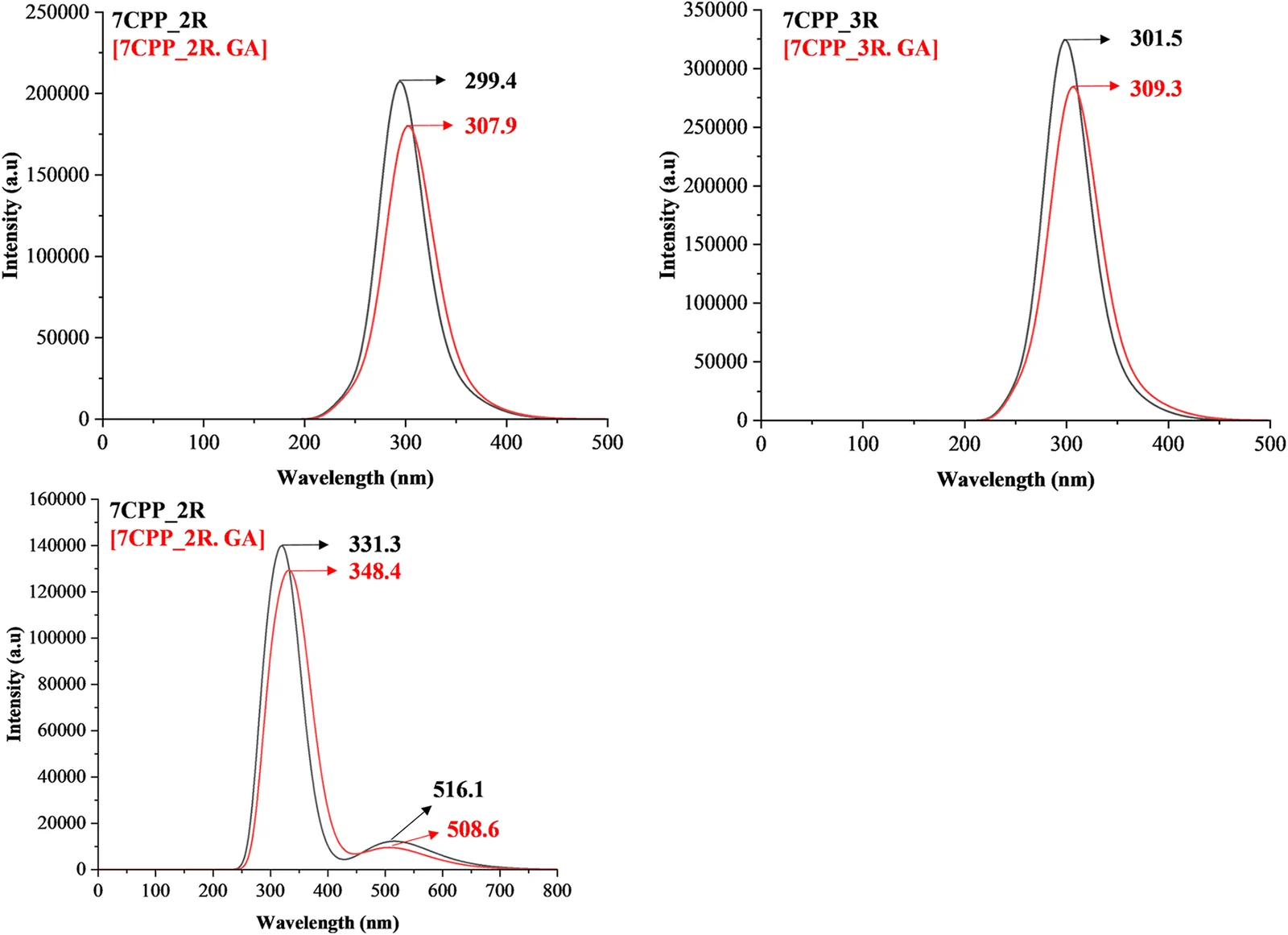
Above UV–vis spectra of the G-series nerve agents encapsulated complex systems with **7CPP_2R** and **7CPP_3R** hosts and below the emission spectrum of the G-series nerve agents encapsulated complex system with supramolecular pinwheel fragment **7CPP_2R** host employing the M06-2X-D3/6-31+G­(d) level of theory in the gas phase.

In the case of parent pinwheel host **7CPP_3R**, the computed gas-phase UV–vis absorption maximum was found to be 301.5 nm, which is in agreement with the experimentally reported value of the pinwheel host.^27^ Upon encapsulation within this host, a remarkable red shift is observed (Figure 8). The calculated spectral shifts demonstrate that guest binding significantly modulates the host’s electronic structure, yielding a distinct optical response. This electronic perturbation provides a viable mechanism for fluorescence-based sensors, where analyte recognition is monitored through changes in emission wavelength or fluorescence intensity. Furthermore, the predicted absorption shifts highlight the potential for colorimetric sensing platforms, where complexation induces an observable color change. Notably, in all of these cases, the encapsulated Tabun molecule exhibits a distinctive diagnostic pattern when encapsulated within the cavity of the supramolecular pinwheel hosts. These encapsulation events imply that the supramolecular pinwheel host can be effectively employed for the size-selective recognition of G-series nerve agents.

### 3.1 Recognition of G-Series Nerve Agents Using Tetracene-Based Supramolecular Single and Double Hooped Hosts

Furthermore, to advance our understanding, we extended the current study by modeling tetracene-based supramolecular hosts **TET_7R1** and **TET_7R2** to explore their potential for size-selective recognition of G-series nerve agents. The binding free energies of the encapsulation complexes between these hosts and the G-series nerve agents are presented in Table 3. Notably, the tetracene-based host exhibits efficient binding toward Soman (GD) and Cyclosarin (GF) within its cavity. The structural complementarity between Soman and Cyclosarin and the **TET_7R1** host cavity (Figure S2) enables effective accommodation, resulting in a higher binding energy than for the other G-series nerve agents. The equilibrium association constants indicate that the TET_7R1 host exhibits the strongest binding preference for the GF and GD nerve agents and shows greater selectivity than the other investigated nerve agents (Table 3). To evaluate the effect of solvent on the equilibrium association constants (*K*_a_), we investigated the representative complexes **[TET_7R1·GD]** and **[TET_7R1·GF]**. The calculated (*K*_a_) values in the solvent phase ((5.5 × 10^3^ M^–1^) and (7.7 × 10^3^ M^–1^), respectively) are lower than the corresponding gas-phase values. This reduction can be attributed to solvent screening and competitive solvation effects, which weaken the host–guest interactions in solution. In contrast, the absence of solvent effects in the gas phase results in stronger binding interactions, where hydrogen bonding, electrostatic interactions, dispersion forces, and CH···π interactions contribute more effectively to the stabilization of the complexes, leading to higher calculated (*K*_a_) values. This pronounced selectivity toward agents GF and GD may be due to preferential host–guest association, which provides a distinct advantage for sensing applications by enabling enhanced nerve agents recognition. Consequently, only Soman and Cyclosarin were considered for complexation with the double-hooped **TET_7R2** supramolecular host (Figure S6). The optimized complex geometries of the single-hooped host are shown in Figure 9. Additionally, the total binding energy was normalized by the number of host cores to yield an average interaction energy per core (Table 3). Since the present analysis was intended only to provide a normalized comparison of the interaction strengths between the two hosts, rather than to derive stepwise binding constants, cooperative effects were not explicitly considered.

**Figure 9:**
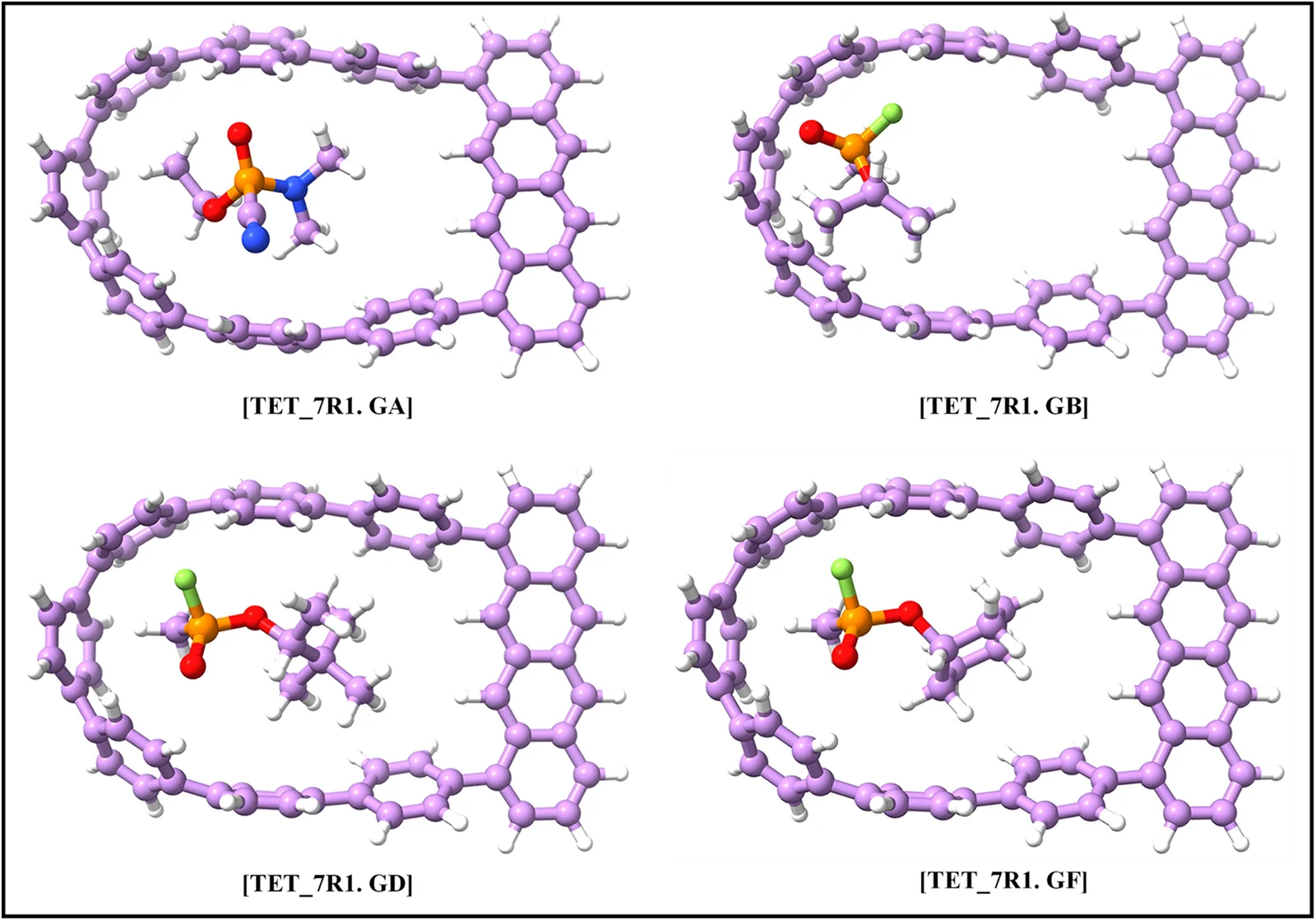
Optimized encapsulated complex structures of **TET_7R1** with G-series nerve agents, employing the M06-2X-D3/6-31+G­(d) level of theory in the gas phase. In the host, carbon is shown in magenta. In nerve agents, the colors green, blue, red, orange, and white represent the fluorine, nitrogen, oxygen, phosphorus, and hydrogen atoms, respectively, in a ball-and-stick model.

**Table 3:** Computed Binding Energies, the Counterpoise-Corrected Interaction Energies (Δ*E*_CP_), Binding Free Energies, and the Equilibrium Association Constants (*K*_a_) of the G-Series Nerve Agents Encapsulated Complex within the Supramolecular **TET_7R1** and **TET_7R2** Host Systems Using M06-2X-D3/6-31+G­(d) Level of Theory in the Gas Phase^a^

system	Δ*E*	*ΔE*_CP_	Δ*G*	*K*_a_ (M^–1^)
**[TET_7R1. GA]**	**–23.1**	**–23.0**	**–6.0**	**2.5 × 10** ^**4**^
**[TET_7R1. GD]**	**–25.3**	**–23.8**	**–8.5**	**1.7 × 10** ^**6**^
**[TET_7R1. GF]**	**–25.1**	**–23.5**	**–8.9**	**3.4 × 10** ^**6**^
**[TET_7R1. GB]**	**–16.8**	**–15.5**	**–1.9**	**2.5 × 10** ^**1**^
				
**[TET_7R2. GD]**	**–50.4 (−25.2)**	**–47.8**	**–17.0 (−8.5)**	**2.9 × 10** ^**12**^
**[TET_7R2. GF]**	**–49.9 (−24.95)**	**–47.1**	**–18.4 (−9.2)**	**3.1 × 10** ^**13**^

a All energies are given in kcal/mol. The average binding energies are given in parentheses.

In all of these G-series nerve agent encapsulated complexes, stabilization is primarily governed by the CH···π and other van der Waals noncovalent interactions, as supported by NCI analysis (Figure 10) and AIM data (Table S4). Additionally, the structural complementarity allows the guest molecules to fit effectively within the cavity of the host molecules.

**Figure 10:**
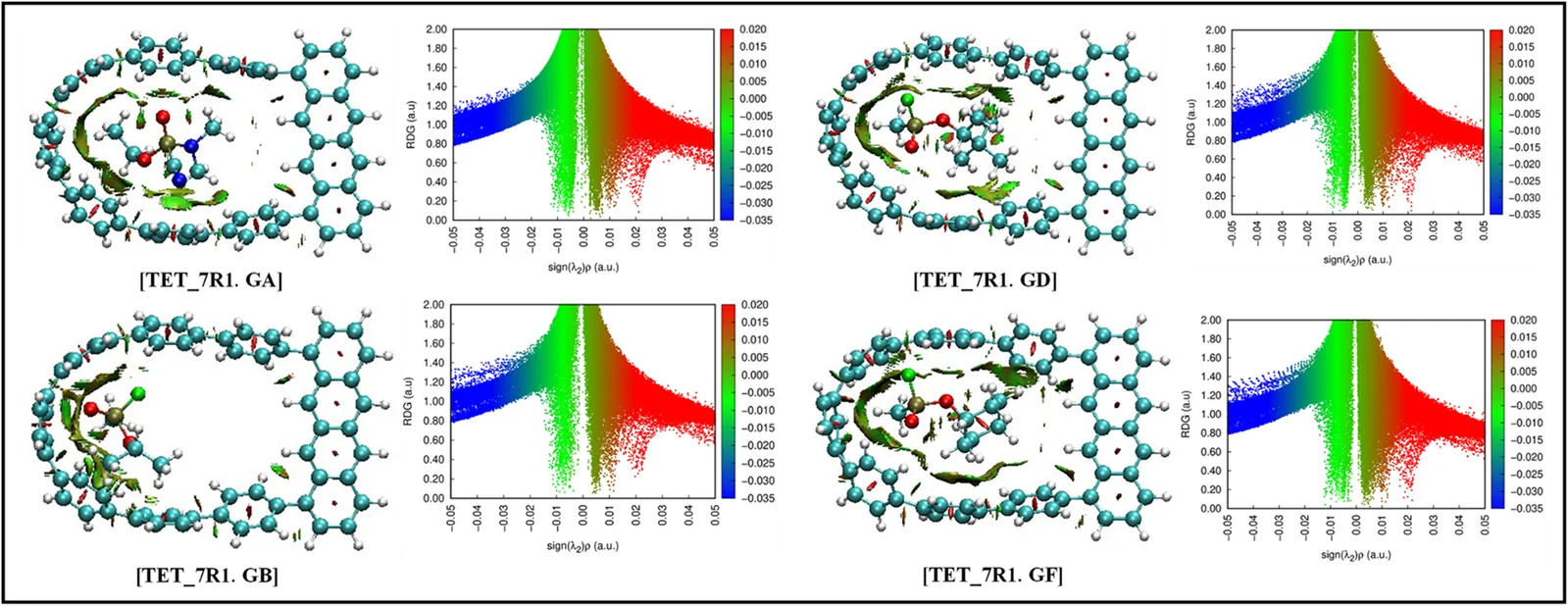
Reduced density gradient (RDG) isosurface analysis, along with the corresponding NCI scatter plot on the right, visualizes the noncovalent interactions in the G-series nerve agent encapsulated complexes with the supramolecular tetracene-based host.

In addition, the UV–vis and emission spectral analyses further reveal that Soman undergoes a substantial redshift with respect to the parent single-hooped host **TET_7R1** (Figures 11 and 12, and in Supporting Information Tables S5,S6, and Figures S7,S8). Interestingly, upon complexation with the double-hooped **TET_7R2** host, the wavelength shift is minimal near the parent host. In the case of absorption spectra for Soman and Cyclosarin, it was observed that 310.7 and 312.3 nm, respectively, correspond to the double-hooped **TET_7R2** host 309.2 nm. This was further lowered in the case of emission spectra upon encapsulation of these two G-series nerve agents with a double-hooped host. For Soman, it was observed in a hypsochromic shift of 330.7 nm, and for Cyclosarin, it was observed in 331.7 nm with respect to the parent double-hooped host at 331.2 nm (Figure S9). Upon encapsulation of Soman and Cyclosarin within the double-hooped host–guest complex, only weak perturbation of the electronic structure is observed. The interaction is primarily governed by van der Waals forces, accompanied by limited orbital overlap between the host and guest. The double-hoop geometry maintains the guest at a well-defined and controlled distance from the tetracene core, thereby preventing significant mixing between the host HOMO/LUMO orbitals and the guest orbitals (Figure S10). Furthermore, the rigid framework of the **TET_7R2** host inhibits structural distortion upon complexation, resulting in only minimal changes in the electronic energy levels (Table S7) and, consequently, negligible shifts in the absorption and emission wavelengths. The cooperative confinement effect imposed by the dual hoop cavities leads to significant destabilizations, and consequently, the absorption and emission bands of the newly formed double-hooped host–guest assembly exhibited negligible shifts, reflecting the distinct spectral signature of the encapsulated complex.^56^ These spectral changes can serve as diagnostic indicators for the size-selective encapsulation of G-series nerve agents.

**Figure 11:**
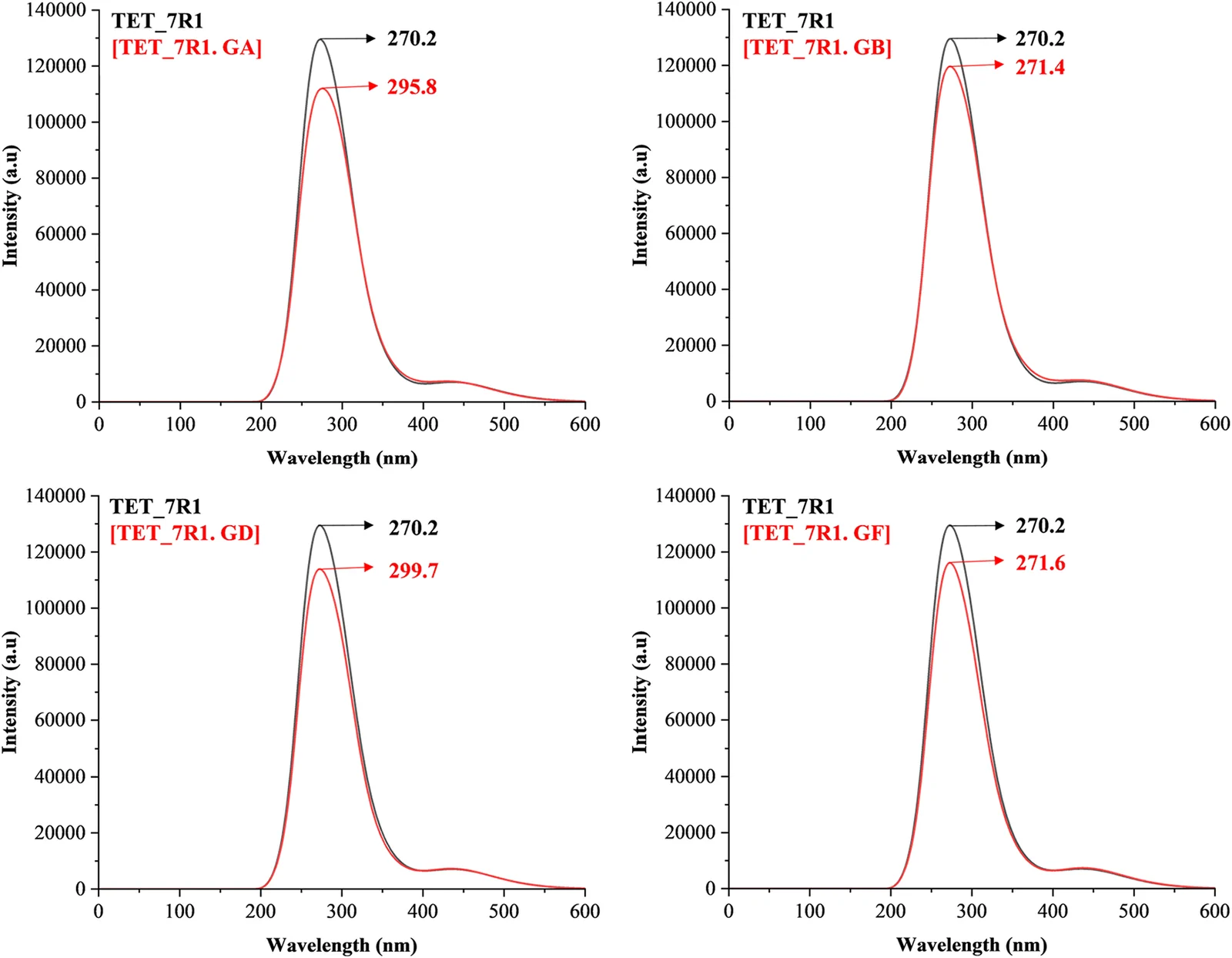
UV–vis spectra of the G-series nerve agents encapsulated complex system with supramolecular **TET_7R1** host employing the M06-2X-D3/6-31+G­(d) level of theory in the gas phase.

**Figure 12:**
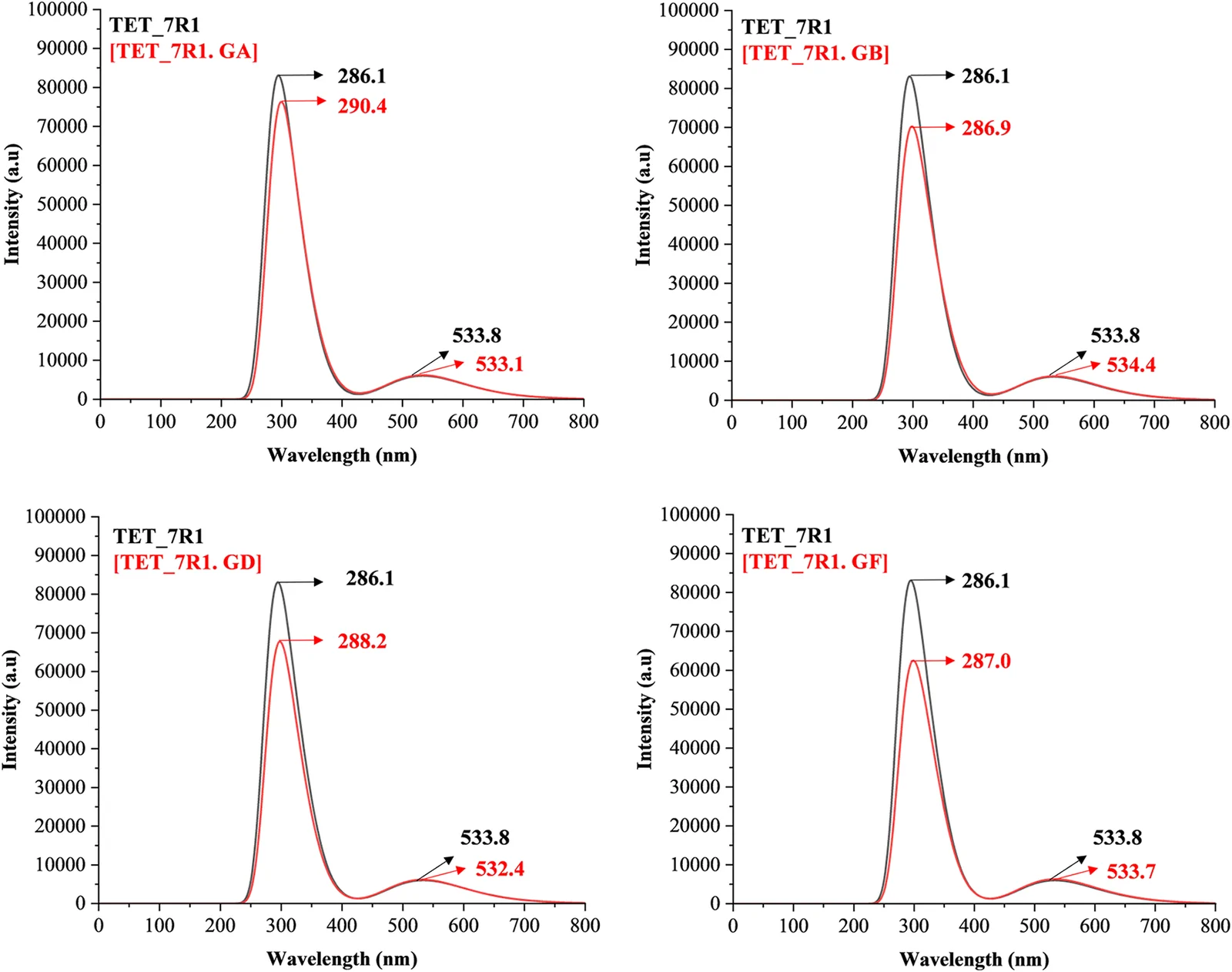
Emission spectra of the G-series nerve agent encapsulated complexes with the supramolecular **TET_7R1** host, employing the M06-2X-D3/6-31+G­(d) level of theory in the gas phase.

### 3.2 EDA Analysis

To gain a deeper understanding of the weak interactions in the studied complex systems involving the supramolecular pinwheel, tetracene-based hosts, and the G-series nerve agents, an energy decomposition analysis (EDA) was performed using symmetry-adapted perturbation theory (SAPT). The total SAPT interaction energy, as well as individual energy terms (including electrostatic and exchange-repulsion, induction, and dispersion), were determined for the nerve agents embedded complex systems, and all energy terms are shown in Table 4.

**Table 4:** Energy Decomposition Analysis (EDA) of the Studied G-Series Nerve Agents Encapsulated Complex Systems^a^

system	*E*_elect_	*E*_ind_	*E*_disp_	*E*_exch_	*E*_SAPT0_
**[7CPP_1R. GA]**	–16.0 (32.4%)	–4.0 (8.1%)	–29.4 (59.5%)	30.8	–18.6
**[7CPP_1R. GB]**	–13.9 (35.6%)	–3.5 (8.9%)	–21.6 (55.4%)	24.3	–14.7
**[7CPP_1R. GD]**	–15.0 (33.7%)	–3.8 (8.5%)	–25.7 (57.8%)	29.7	–14.8
**[7CPP_1R. GF]**	–17.0 (35.3%)	–4.6 (9.6%)	–26.5 (55.1%)	31.5	–16.6
					
**[TET_7R1. GA]**	–16.5 (34.6%)	–4.0(8.4%)	–27.2 (57.0%)	31.2	–16.5
**[TET_7R1. GB]**	–13.0 (38.9%)	–3.2(9.6%)	–17.2 (51.5%)	22.5	–10.9
**[TET_7R1. GD]**	–16.0 (33.6%)	–3.7 (7.8%)	–27.9 (58.6%)	30.0	–17.6
**[TET_7R1. GF]**	–16.1 (33.8%)	–3.8 (8.0%)	–27.7 (58.2%)	29.0	–18.6

a The interaction energy components were obtained by using the SAPT0/def2-SVP level of theory. All energies are given in kcal/mol.

In all of the encapsulation complexes, the SAPT0 analysis reveals that the exchange-repulsion (*E*_exch_) energy term, representing the steric repulsion, contributes positively, whereas the remaining components, such as electrostatic, induction, and dispersion energy terms, contribute negatively, reflecting their attractive roles in stabilizing the nerve agent encapsulated complex within the host cavities. The EDA results further indicate that, across all G-series nerve agent encapsulation systems, the attractive interaction energy is predominantly governed by dispersion, accounting for more than 55% of the total attractive energy. An exception is observed for the Sarin (GB) encapsulation complex within the tetracene host cavity, where the dispersion contribution is slightly lower at 51.5%.

## 4 Conclusions

In conclusion, the present study elucidates the noncovalent and size-selective recognition of the G-series nerve agents within the cavities of pinwheel- and tetracene-based supramolecular hosts and highlights the fundamental nature of the interactions between these supramolecular hosts and the G-series nerve agents, as investigated through DFT-based computational methods. The DFT calculations reveal that the guest nerve agent Tabun is energetically favored for encapsulation within the electron-rich deep cavity of the pinwheel supramolecular host. The higher affinity of Tabun demonstrates that the supramolecular pinwheel host offers superior structural complementarity for effective guest accommodation. This enhanced binding is primarily governed by multiple F···H, CH···π, and van der Waals noncovalent interactions. Similarly, a tetracene-based supramolecular host provides suitable accommodation for the Soman (GD) and Cyclosarin (GF) nerve agents within its electron-rich host cavity, where stabilization arises from comparable noncovalent interactions observed in the Pinwheel host systems. Furthermore, the host molecules studied here are capable of binding multiple nerve agent molecules, forming 1:2 and 1:3 host–guest complexes. The NCI analysis further reveals that a diverse range of weak interactions plays a crucial role in stabilizing the encapsulation of Tabun, Soman, and Cyclosarin within the pinwheel and tetracene-based host cavities. Complementary atoms-in-molecules (AIM) topological analysis distinguishes the nature of these interactions, identifying F···H, CH···π, and van der Waals interactions as the key contributors to the formation of these size-selective inclusion complexes.

TD-DFT results demonstrate that upon complexation, the UV–Vis absorption and emission spectra of these size-selective nerve agents exhibit pronounced red shifts relative to the parent supramolecular hosts. These optoelectronic responses provide distinct diagnostic signatures for the recognition of size-selective G-series nerve agents. Furthermore, the EDA analysis using SAPT0 reveals that dispersion interactions are the dominant stabilizing factors governing the formation of these host–guest complexes. In summary, this theoretical investigation of size-selective and complementary supramolecular host–guest chemistry may provide a valuable foundation for future experimental studies. The results suggest that the supramolecular pinwheel host enables efficient size-selective encapsulation of G-series nerve agents. Moreover, the designed tetracene-based host, or structurally analogous hosts, is anticipated to serve as a promising platform for developing advanced photoresponsive supramolecular hosts for the size-selective recognition of various nerve agents.

## Supplementary Material



## Data Availability

The data of this article have been provided in the SI.

## Notes

Additional Tables, figures, and coordinates of the geometries in xyz format. Binding energies, AIM analysis, calculated UV–visible optoelectronic properties, calculated emission optoelectronic properties, strain energy calculations, optimized complex geometries, UV–visible line spectra and emission line spectra, HOMO and LUMO isosurfaces (PDF)
