# Supplementary material for: Size Selective Recognition of G‑Series Nerve Agents via Aryl–C–H Interactions with the Supramolecular Pinwheel and Tetracene-Based Host: A Computational Study
Source: ACS Omega. 2026 Aug 25;11(35):52994–3009. doi: 10.1021/acsomega.6c05223 (PMC13563624; doi:10.1021/acsomega.6c05223)
Supplement: Supplementary file 1 [file ao6c05223_si_001.pdf]

Size Selective Recognition of G-series nerve agents *via* aryl-C-H interactions with the Supramolecular Pinwheel and Tetracene-based Host: A Computational Study

Dipankar Das[a,b], Rabindranath Lo[c*], Bishwajit Ganguly[a,b,d*]

[a] Computational and Simulation Unit (Marine Elements and Marine Environment Division and Centralized Instrument Facility), CSIR- Central Salt & Marine Chemicals Research Institute, Bhavnagar-364002, Gujarat, India.

[b] Academy of Scientific and Innovative Research (AcSIR), Ghaziabad 201002, Uttar Pradesh, India.

[c]Institute of Organic Chemistry and Biochemistry, Czech Academy of Sciences, Flemingovo nám. 542/2, Prague 6 160 00, Czech Republic

[d]CSIR-Central Glass and Ceramic Research Institute, Kolkata 700032, India

*Corresponding Author. Fax: (+91)-278-2567562, E-mail: gangulycsmcri@gmail.com, rabindranath.lo@uochb.cas.cz

Table S1. The calculated binding energies and binding energies of the encapsulated complex system between the host **6CPP_1R** and the tetracene-based host **TET_6R1** with G-series nerve agents employing M06-D3/6-31+G(d) level of theory in the gas phase. All energies are given in kcal/mol.

System	ΔE	ΔG
**[6CPP_1R. GA]**	**-23.8**	**-8.0**
**[6CPP_1R. GD]**	**-20.3**	**-4.5**
**[6CPP_1R. GF]**	**-21.3**	**-4.2**
**[6CPP_1R. GB]**	**-18.8**	**-3.8**
**[TET_6R1_GA]**	**-19.7**	**-4.2**
**[TET_6R1_GB]**	**-19.6**	**-4.2**
**[TET_6R1_GD]**	**-20.5**	**-2.0**
**[TET_6R1_GF]**	**-22.9**	**-6.0**

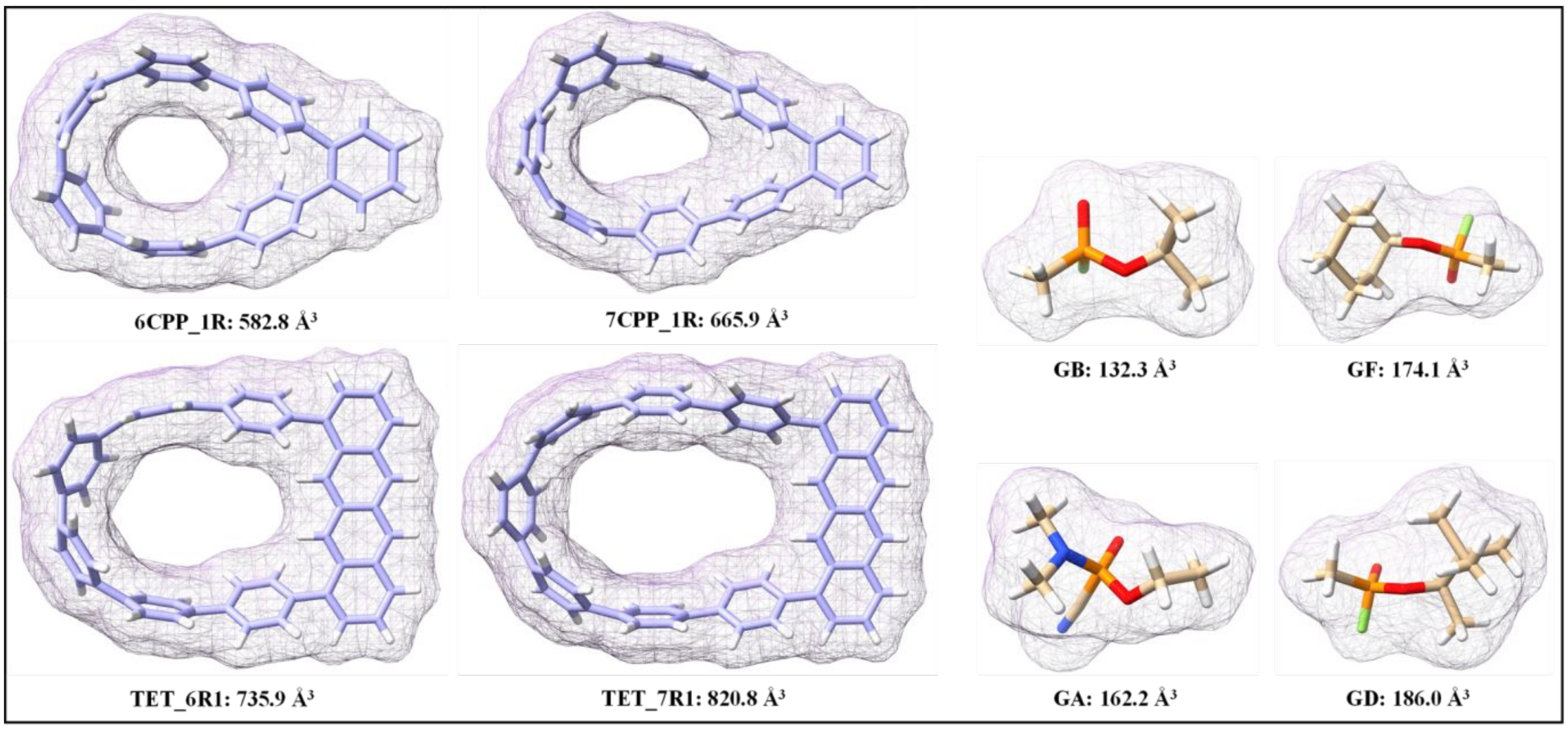

Figure S1. The van der Waals surface of the supramolecular pinwheel and tetracene-based hosts (Spartan 08, MMFF). The chemical structures of G-series nerve agents and their corresponding van der Waals surfaces are shown on the right. The size and shape increase according to the series. Values are given in Angstrom.

Table S2. The calculated single-point energies of the complex system between these supramolecular pinwheel and tetracene-based hosts and G-series nerve agents.

System	Single-point at M06-2X-D3/6-311++G(d,p) level of theory in gas phase **(ΔE)**	Single-point at M06-2X-D3/6-31+G(d) level of theory in DCM **(ΔE)**
[7CPP_1R. GA]	-28.8	-22.2
[7CPP_1R. GD]	-25.4	-18.0
[7CPP_1R. GF]	-25.8	-19.3
[7CPP_1R. GB]	-22.4	-15.6
**[7CPP_2R. GA]**	**-56.8**	**-43.8**
**[7CPP_3R. GA]**	**-84.4**	**-65.1**
**[TET_7R1. GF]**	**-29.5**	**-23.0**
**[TET_7R1. GD]**	**-27.8**	**-21.0**
**[TET_7R1. GA]**	**-25.7**	**-19.1**
**[TET_7R1. GB]**	**-19.3**	**-12.7**
**[TET_7R2. GD]**	**-56.0**	**-42.5**
**[TET_7R2. GF]**	**-58.9**	**-46.0**

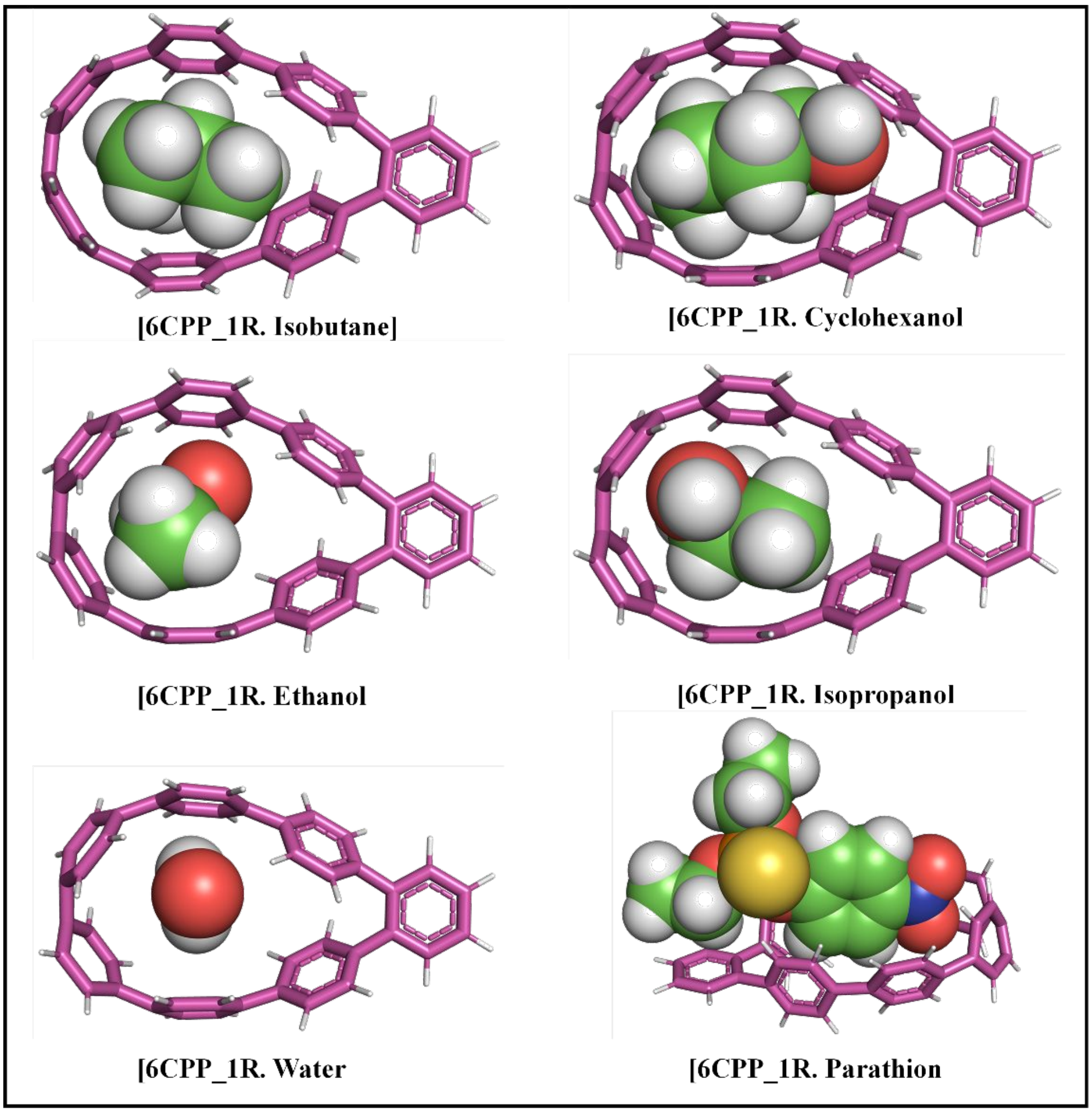

Figure S2. The optimized complex geometries between the smaller guest molecules and host **6CPP_1R**. The representation of these complex systems is shown in tube shape. The host's magenta and white colors signify the carbon and hydrogen atoms. For smaller guest carbon, oxygen, and hydrogen are represented in green, red, and white colors in space-filling model.

Table S3. The calculated energies of the complex system between the host **6CPP_1R** and four small molecules such as cyclohexanol, isobutene, isopropanol, and ethanol employing the M06-2X-D3/6-31+G(d) level of theory in the gas phase. All energies are given in kcal/mol.

System	ΔE	ΔG
**[6CPP_1R. Cyclohexanol]**	**-12.4**	**2.0**
**[6CPP_1R. Isobutane]**	**-12.4**	**1.6**
**[6CPP_1R. Isopropanol]**	**-12.1**	**0.7**
**[6CPP_1R. Ethanol]**	**-12.4**	**0.6**
**[6CPP_1R. Water]**	**-9.3**	**0.02**
**[6CPP_1R. Parathion]**	**-23.4**	**-5.9**

Table S4. The calculated atoms in molecules (AIM) analysis for the encapsulated complex system between the pinwheel and tetracene-based hosts and the nerve agent at the M06-2X-D3/6-31+G(d) level of theory in the gas phase. The topological parameters of this system are $G_{(r)}$ = Lagrangian kinetic energy, $K_{(r)}$ = Hamiltonian kinetic energy, $V_{(r)}$ = potential energy density, $H_{(r)}$ = energy density, and $\nabla^2_{\rho(r)}$ = Laplacian electron density calculated with AIM analysis.

	r	ρ (r)	G(r)	K(r)	V(r)	H(r)	$\nabla^2\rho(r)$	|V(r)|/G(r)
				[7CPP_1R. GA]				
**O…H-C**	**3.2**	0.00236	0.00157	-0.00056	-0.00100	0.00056	0.00854	0.63694
	**2.6**	0.00722	0.00575	-0.00102	-0.00472	0.00102	0.02710	0.82087
**C-H…π**	**2.9**	0.00531	0.00326	-0.00084	-0.00241	0.00084	0.01641	0.73926
	**3.5**	0.00294	0.00393	-0.00093	-0.00300	0.00093	0.01948	0.76336
	**2.9**	0.00523	0.00309	-0.00074	-0.00234	0.00074	0.01539	0.75728
	**2.6**	0.00836	0.00596	-0.00128	-0.00468	0.00128	0.02897	0.78523
	**2.6**	0.00591	0.00390	-0.00093	-0.00297	0.00093	0.01935	0.76154
	**2.7**	0.00728	0.00513	-0.00114	-0.00398	0.00114	0.02513	0.77583
	**2.9**	0.00523	0.00309	-0.00074	-0.00234	0.00074	0.01539	0.75728
**N…H-C**	**2.8**	0.00533	0.00358	.-0.00085	-0.00272	0.00085	0.01775	0.75978
				[7CPP_1R. GB]				
**O…H-C**	**2.4**	0.01130	0.00932	-0.00097	-0.00835	0.00097	0.04122	0.89592
**F…H-C**	**2.4**	0.00825	0.00774	-0.00152	-0.00622	0.00152	0.03700	0.80362
**C-H…π**	**2.9**	0.00206	0.00142	-0.00044	-0.00098	0.00044	0.00749	0.69014
	**2.7**	0.00728	0.00476	-0.00111	-0.00365	0.00111	0.02355	0.76681
	**2.8**	0.00666	0.00450	-0.00108	-0.00342	0.00108	0.02236	0.76
	**3.1**	0.00296	0.00179	-0.00060	-0.00118	0.00060	0.00889	0.65922
	**3.2**	0.00284	0.00552	-0.00043	-0.00432	0.00043	0.00784	0.83076
				[7CPP_1R. GD]				
**O…H-C**	**2.4**	0.01157	0.00946	-0.00100	-0.00846	0.00100	0.03140	0.89429
**F…H-C**	**2.4**	0.00504	0.00432	-0.00127	-0.00305	0.00127	0.02240	0.70602
	**2.7**	0.00814	0.00769	-0.00144	-0.00625	0.00144	0.03650	0.81274
**C-H…π**	**2.7**	0.00755	0.00512	-0.00145	-0.00366	0.00146	0.02635	0.71484
	**2.9**	0.00530	0.00347	-0.00085	-0.00262	0.00085	0.01732	0.75504
	**2.7**	0.00737	0.00477	-0.00113	-0.00364	0.00113	0.02361	0.7631
	**2.9**	0.00577	0.00345	-0.00082	-0.00263	0.00082	0.01711	0.76232
	**2.7**	0.00614	0.00418	-0.00120	-0.00297	0.00120	0.02154	0.71053
	**3.2**	0.00290	0.00173	-0.00050	-0.00123	0.00050	0.00895	0.71098
				[7CPP_1R. GF]				
**F…H-C**	**2.8**	0.00400	0.00335	-0.00112	-0.00222	0.00112	0.01792	0.66269
**O…H-C**	**2.6**	0.00827	0.00661	-0.00113	-0.00547	0.00113	0.03099	0.82753
	**2.3**	0.01335	0.01118	-0.00101	-0.01019	0.00101	0.04880	0.91145
**C-H…π**	**2.5**	0.10304	0.00742	-0.00139	-0.00603	0.00139	0.03525	0.81267
	**2.9**	0.00509	0.00303	-0.00071	-0.00231	0.00071	0.01497	0.76238
	**3.0**	0.00413	0.00243	-0.00065	-0.00177	0.00065	0.01236	0.7284
	**2.8**	0.00579	0.00365	-0.00098	-0.00267	0.00098	0.01856	0.73151
	**3.0**	0.00450	0.00286	-0.00067	-0.00219	0.00067	0.01413	0.76573
	**2.7**	0.00689	0.00486	-0.00113	-0.00372	0.00113	0.02401	0.76543

	**2.7**	0.00759	0.00501	-0.00114	-0.00387	0.00114	0.02462	0.77246
**[7CPP_2R. GA]**								
**O…H-C**	**2.6**	0.00729	0.00581	-0.00103	-0.00478	0.00130	0.02737	0.82272
	**3.2**	0.00222	0.00145	-0.00052	-0.00092	0.00052	0.00792	0.63448
	**3.2**	0.00229	0.00151	-0.00054	-0.00096	0.00054	0.0082	0.63576
	**2.7**	0.00620	0.00486	-0.00101	-0.00384	0.00101	0.02354	0.79012
**N…H-C**	**2.6**	0.00727	0.00555	-0.00082	-0.00473	0.00082	0.02549	0.85225
	**2.6**	0.00846	0.00635	-0.00074	-0.00560	0.00074	0.02838	0.88189
	**2.8**	0.00558	0.00379	-0.00091	-0.00287	0.00091	0.01884	0.75726
	**2.8**	0.00560	0.00380	-0.00092	-0.00288	0.00092	0.00189	0.75789
**C-H…π**	**2.9**	0.00626	0.00395	-0.00086	-0.00309	0.00086	0.01926	0.78228
	**2.6**	0.00839	0.00600	-0.00128	-0.00472	0.00128	0.02915	0.78667
	**2.7**	0.00752	0.00533	-0.00117	-0.00415	0.00117	0.02603	0.77861
	**2.9**	0.00546	0.00353	-0.00085	-0.00268	0.00085	0.01755	0.75921
	**3.0**	0.00505	0.00297	-0.00071	-0.00226	0.00071	0.01474	0.76094
	**2.9**	0.00551	0.00341	-0.00089	-0.00252	0.00089	0.01724	0.739
	**3.0**	0.00507	0.00299	-0.00071	-0.00227	0.00071	0.01480	0.7592
	**2.7**	0.00728	0.00514	-0.00114	-0.00399	0.00114	0.02513	0.77626
	**2.6**	0.00867	0.00625	-0.00131	-0.00494	0.00131	0.03028	0.7904
**[7CPP_3R. GA]**								
**O…H-C**	**2.6**	0.00671	0.00530	-0.001103	-0.00427	0.00103	0.02536	0.80566
	**3.2**	0.00225	0.00148	-0.00053	-0.00094	0.00053	0.00807	0.63514
	**2.7**	0.00631	0.00496	-0.00102	-0.00394	0.00102	0.00239	0.79435
	**2.6**	0.00653	0.00515	-0.00102	-0.00412	0.00102	0.02472	0.8
	**3.2**	0.00229	0.00151	-0.00054	-0.00096	0.00054	0.00823	0.63576
**N…H-C**	**2.8**	0.00566	0.00386	-0.00093	-0.00292	0.00093	0.01918	0.75648
	**2.8**	0.00563	0.00383	-0.00092	-0.00290	0.00092	0.01905	0.75718
	**2.8**	0.00561	0.00381	-0.00092	-0.00289	0.00092	0.01896	0.75853
**C-H…π**	**2.9**	0.00556	0.00346	-0.00090	-0.00256	0.00090	0.01745	0.73988
	**2.6**	0.00893	0.00547	-0.00133	-0.00513	0.00133	0.03120	0.93784
	**2.7**	0.00741	0.00524	-0.00115	-0.00508	0.00115	0.02562	0.96947
	**3.2**	0.00523	0.00310	-0.00074	-0.00236	0.00074	0.00154	0.76129
	**2.8**	0.00570	0.00356	-0.00092	-0.00263	0.00092	0.01795	0.73876
	**2.6**	0.00868	0.00626	-0.00130	-0.00495	0.00130	0.03028	0.79073
	**3.0**	0.00628	0.00395	-0.00086	-0.00308	0.00086	0.01928	0.77975
	**2.7**	0.00739	0.00523	-0.00115	-0.00407	0.00115	0.02554	0.7782
	**2.9**	0.00552	0.00355	-0.00085	-0.00269	0.00085	0.01765	0.75775
	**3.0**	0.00511	0.00302	-0.00072	-0.00230	0.00072	0.01500	0.76159
	**2.8**	0.00563	0.00350	-0.00091	-0.00259	0.00091	0.01767	0.74
	**3.5**	0.00603	0.00403	-0.00094	-0.00308	0.00094	0.01992	0.76427

	**2.7**	0.00748	0.00530	-0.00116	-0.00414	0.00116	0.02588	0.78113
	**2.9**	0.00555	0.00358	-0.00086	0.00271	0.00086	0.01777	0.756983
**[TET_7R1. GA]**								
**O…H-C**	**2.6**	0.00757	0.00623	-0.00113	-0.00509	0.00113	0.02949	0.81701
	**2.6**	0.00795	0.00643	-0.00108	-0.00535	0.00108	0.03011	0.83204
**C-H…π**	**2.8**	0.00742	0.00510	-0.00126	-0.00383	0.00126	0.02547	0.75098
	**2.7**	0.00755	0.00503	-0.00128	-0.00375	0.00128	0.02530	0.74553
	**2.8**	0.00677	0.00469	-0.00111	-0.00358	0.00111	0.02323	0.76333
	**2.8**	0.00401	0.00251	-0.00059	-0.00192	0.00059	0.01240	0.76494
	**3.0**	0.00494	0.00335	-0.00074	-0.00260	0.00074	0.01642	0.77612
	**2.8**	0.00697	0.00447	-0.00104	-0.00343	0.00104	0.02206	0.76734
	**2.9**	0.00555	0.00345	-0.00081	-0.00264	0.00081	0.01706	0.76522
	**2.9**	0.00517	0.00320	-0.00079	-0.00240	0.00079	0.01599	0.75
**[TET_7R1. GB]**								
**F…H-C**	**2.3**	0.00979	0.00948	-0.00118	-0.00829	0.00118	0.00426	0.87447
**O…H-C**	**2.5**	0.00884	0.00692	-0.00112	-0.00579	0.00112	0.03219	0.83671
**C-H…π**	**2.7**	0.00670	0.00441	-0.00109	-0.00331	0.00109	0.02203	0.75057
	**2.7**	0.00698	0.00496	-0.00111	-0.00385	0.00111	0.02433	0.77621
**[TET_7R1. GD]**								
**F…H-C**	**2.3**	0.01005	0.00955	-0.00112	-0.00843	0.00112	0.04273	0.88272
**O…H-C**	**2.4**	0.01111	0.00935	-0.00114	-0.00820	0.00114	0.04197	0.87701
**C-H…π**	**2.7**	0.00775	0.00555	-0.00122	-0.00433	0.00122	0.02712	0.780148
	**2.7**	0.00711	0.00448	-0.00106	-0.00341	0.00106	0.02218	0.76116
	**3.0**	0.00408	0.00244	-0.00066	-0.00178	0.00066	0.01243	0.72951
	**2.8**	0.00627	0.00407	-0.00095	-0.00311	0.00095	0.02009	0.76413
**[TET_7R1. GF]**								
**F…H-C**	**2.3**	0.00975	0.00933	-0.00117	-0.00816	0.00117	0.04203	0.8746
**O…H-C**	**2.4**	0.00749	0.00596	-0.00112	-0.00484	0.00112	0.02837	0.81208
	**2.7**	0.01058	0.00887	-0.00120	-0.00767	0.00120	0.04031	0.86471
**C-H…π**	**2.6**	0.00812	0.00560	-0.00137	-0.00422	0.00137	0.02790	0.75357
	**2.6**	0.00786	0.00569	-0.00123	-0.00445	0.00123	0.02393	0.78207
	**3.0**	0.00469	0.00294	-0.00071	-0.00223	0.00071	0.01463	0.7585
	**2.6**	0.00831	0.00555	-0.00130	-0.00424	0.00130	0.03013	0.76396
	**2.9**	0.00509	0.00298	-0.00078	-0.00220	0.00078	0.01509	0.73826
**[TET_7R2. GD]**								
**F…H-C**	**2.3**	0.01099	0.01046	-0.00100	-0.00945	0.00100	0.04588	0.90344
	**2.3**	0.00524	0.00328	-0.00072	-0.00256	0.00072	0.01603	0.78049
**O…H-C**	**2.6**	0.00764	0.00609	-0.00112	-0.00497	0.00112	0.02888	0.81609
	**2.4**	0.01132	0.00953	-0.00112	-0.00840	0.00112	0.04264	0.88143

**C-H…π**	**2.7**	0.00721	0.00455	-0.00108	-0.00346	0.00108	0.02255	0.76044
	**2.9**	0.00462	0.00280	-0.00078	-0.00202	0.00078	0.01437	0.72143
	**2.8**	0.00639	0.00405	-0.00105	-0.00299	0.00105	0.02047	0.73827
	**2.9**	0.00515	0.00338	-0.00084	-0.00254	0.00084	0.01658	0.75148
	**2.9**	0.00610	0.00394	-0.00093	-0.00300	0.00093	0.01950	0.76124
	**2.9**	0.00466	0.00283	-0.00078	-0.00204	0.00078	0.01695	0.72085
	**2.7**	0.00728	0.00460	-0.00109	-0.00350	0.00109	0.02279	0.76087
	**2.9**	0.00491	0.00316	-0.00101	-0.00214	0.00101	0.01672	0.67772
	**2.8**	0.00639	0.00405	-0.00105	-0.00299	0.00105	0.02044	0.73827
	**2.9**	0.00518	0.00341	-0.00085	-0.00256	0.00085	0.01708	0.75073
	**2.6**	0.00426	0.00332	-0.00075	-0.00240	0.00075	0.01698	0.72289
**[TET_7R2. GF]**								
**F…H-C**	**2.3**	0.00993	0.00949	-0.00115	-0.00834	0.00115	0.04261	0.87882
	**2.3**	0.00993	0.00950	-0.00115	-0.00834	0.00115	0.04262	0.87789
**O…H-C**	**2.4**	0.01023	0.00853	-0.00118	-0.00735	0.00118	0.03886	0.86166
	**2.6**	0.00758	0.00603	-0.00112	-0.00491	0.00112	0.02863	0.81426
	**2.4**	0.01023	0.00853	-0.00118	-0.00735	0.00118	0.03886	0.86166
	**2.6**	0.00757	0.00603	-0.00112	-0.00491	0.00112	0.01893	0.81426
**C-H…π**	**2.8**	0.00710	0.00465	-0.00109	-0.00356	0.00109	0.02300	0.76559
	**2.6**	0.00822	0.00568	-0.00138	-0.00429	0.00138	0.02827	0.75528
	**2.8**	0.00561	0.00333	-0.00086	-0.00247	0.00086	0.01678	0.74174
	**2.7**	0.00818	0.00540	-0.00131	-0.00409	0.00131	0.02688	0.75741
	**3.1**	0.00398	0.00228	-0.00054	-0.00174	0.00054	0.01134	0.76316
	**3.2**	0.00485	0.00304	-0.00072	-0.00231	0.00072	0.01507	0.75987
	**2.6**	0.00787	0.00570	-0.00124	-0.00446	0.00124	0.02779	0.78246
	**2.8**	0.00561	0.00333	-0.00086	-0.00247	0.00086	0.01678	0.74174
	**2.8**	0.00710	0.00465	-0.00109	-0.00356	0.00109	0.02300	0.76559
	**2.6**	0.00822	0.00568	-0.00138	-0.00429	0.00138	0.02826	0.75528
	**2.7**	0.00818	0.00540	-0.00131	-0.00408	0.00131	0.02687	0.75556
	**3.1**	0.00399	0.00229	-0.00054	-0.00174	0.00054	0.01136	0.75983
	**2.6**	0.00787	0.00570	-0.00124	-0.00446	0.00124	0.02395	0.78246

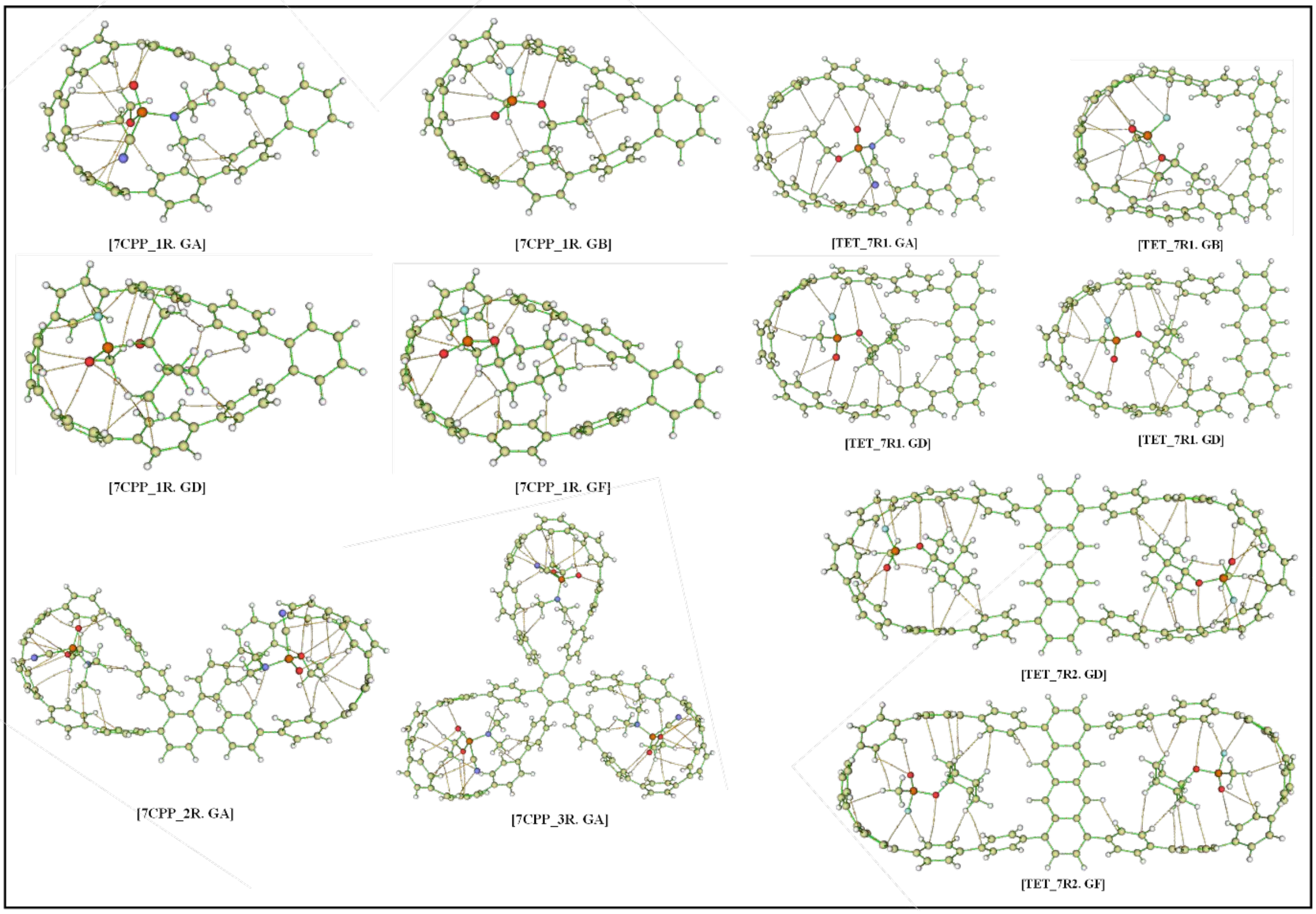

Figure S3. Atoms in molecule (AIM) analysis of these complex systems between host **7CPP_1R**, **7CPP_2R**, and **7CPP_3R**, single and double-hooped tetracene-based **TET_7R1** & **TET_7R2** hosts and G-series nerve agents.

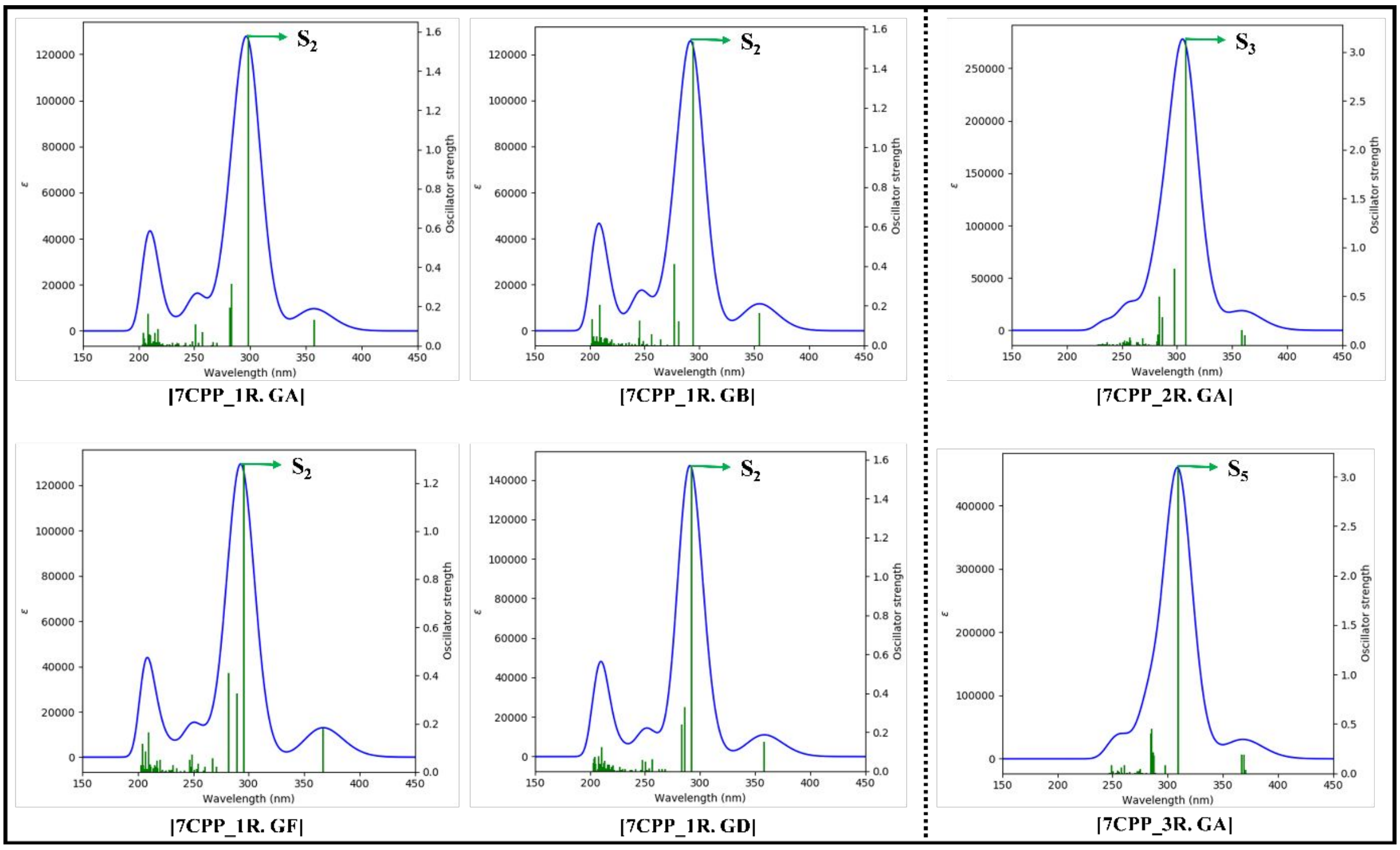

Figure S4. The UV-visible line spectra of the studied complex systems between the supramolecular pinwheel **7CPP_1R**, **7CPP_2R**, and **7CPP_3R** hosts with G-series nerve agents using the M06-2X-D3/6-31+G(d) level of theory in the gas phase. Only Tabun encapsulated on **7CPP_2R**, and **7CPP_3R** pinwheel supramolecular hosts are showed in right side of black the dotted line.

Table S5. The calculated UV-visible optoelectronic properties such as wavelength, oscillator strengths, coefficient and their corresponding transitions of the encapsulated complex system between the supramolecular pinwheel and tetracene-based hosts and G-series nerve agents employing the M062-X-D3/6-31+G(d) level of theory in the gas phase.

System	λmax	*f*	Assignment	Coefficient
**7CPP_1R**	291.9	1.7643	H → L+1	0.48088 (46.2%)
**[7CPP_1R. GA]**	298.6	1.5734	H → L+1	0.50660 (51.3%)
**[7CPP_1R. GB]**	294.0	1.5359	H → L+1	0.49884 (49.7%)
**[7CPP_1R. GD]**	292.6	1.5658	H → L+1	0.47855 (45.8%)
**[7CPP_1R. GF]**	295.8	1.2757	H → L+1	0.45584 (41.5%)
**7CPP_2R**	299.2	3.6165	H → L+2	0.33398 (22.3%)
**[7CPP_2R. GA]**	307.9	3.1268	H → L+2	0.35651 (25.4%)
**7CPP_3R**	301.4	3.4517	H → L+4	0.25350 (12.8%)
**[7CPP_3R. GA]**	309.3	3.0886	H-2 → L+5	0.25365 (12.8%)
**TET_7R1**	270.2	1.159	H-5 → L	0.26067 (13.6%)
**[TET_7R1. GA]**	295.8	0.9636	H-1 → L+2	0.32289 (20.8%)

[TET_7R1. GB]	271.4	0.9895	H-2 → L+2	0.26159 (13.7%)
[TET_7R1. GF]	271.6	0.9617	H-2 → L+2	0.25674 (13.1%)
[TET_7R1. GD]	299.7	0.9211	H-1 → L	0.48298 (46.6%)
TET_7R2	309.2	3.0122	H-2 → L	0.34350 (23.5%)
[TET_7R2. GD]	310.7	2.5767	H-2 → L	0.39491 (31.1%)
[TET_7R2. GF]	312.3	2.5966	H-2 → L	0.38355 (29.4%)

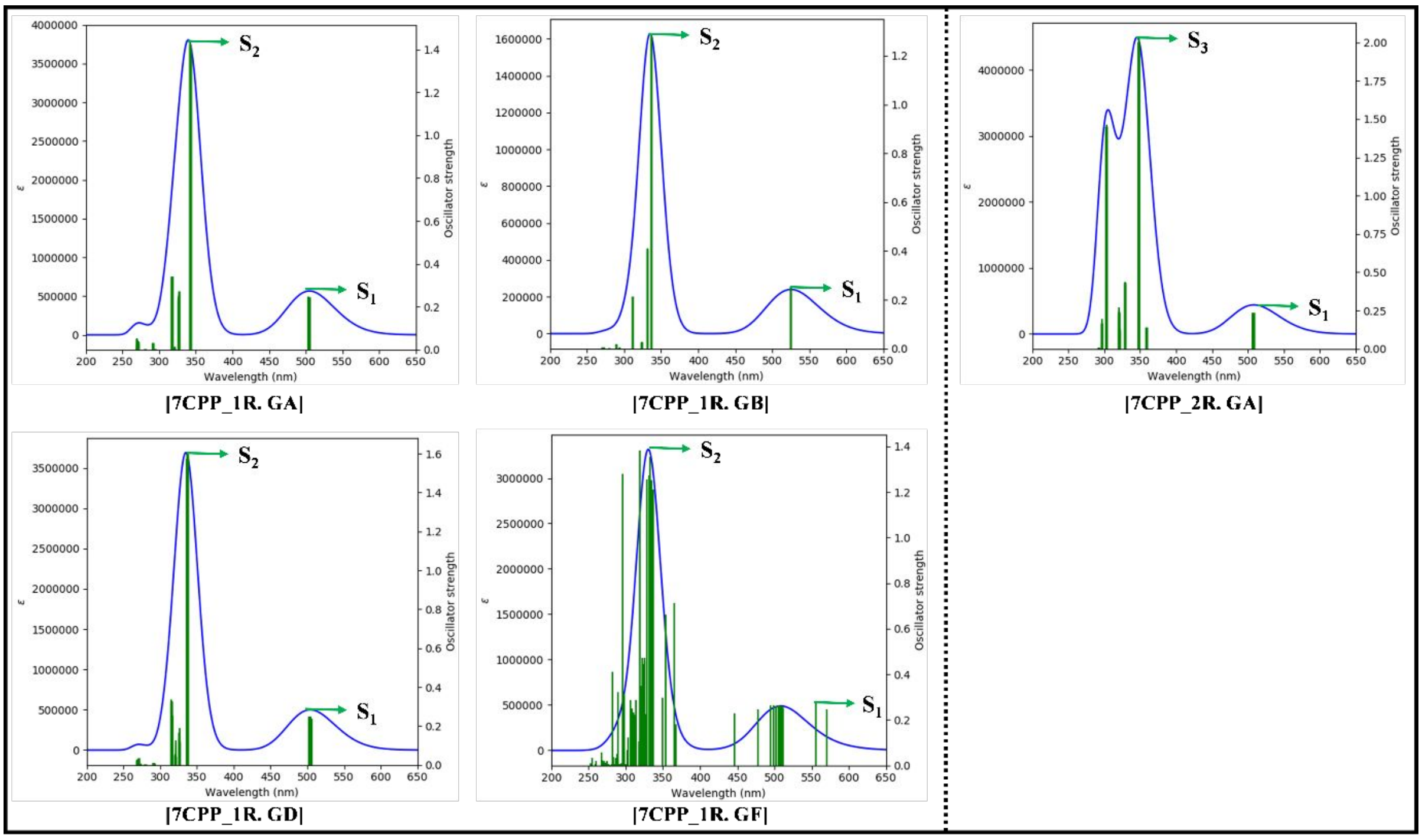

Figure S5. The emission line spectra of the studied complex systems between the supramolecular pinwheel **7CPP_1R**, **7CPP_2R**, and **7CPP_3R** host with G-series nerve agents using the M06-2X-D3/6-31+G(d) level of theory in the gas phase. Only Tabun encapsulated on **7CPP_2R** pinwheel supramolecular host.

Table S6. The calculated emission optoelectronic properties such as wavelength, oscillator strengths, coefficient and their corresponding transitions of the encapsulated complex system between the supramolecular pinwheel and tetracene-based hosts and G-series nerve agents employing the M062-X-D3/6-31+G(d) level of theory in the gas phase.

System	Emission spectra							
	λemi	*f*	Assignment	Coefficient	λemi	*f*	Assignment	Coefficient
7CPP_1R	335.8	1.8564	H → L+1	0.56824 (64.5%)	506.2	0.2927	H → L	0.68818 (94.7%)
[7CPP_1R. GA]	343.6	1.3936	H → L+1	0.62031 (76.9%)	505.6	0.2434	H → L	0.68626 (94.1%)
[7CPP_1R. GB]	337.0	1.2774	H → L+1	0.64329 (82.7%)	525.3	0.2544	H → L	0.69197 (95.7%)
[7CPP_1R. GD]	337.0	1.5986	H → 1-L	0.34214 (23.4%)	504.1	0.2494	H → L	0.68756 (94.5%)
[7CPP_1R. GF]	334.9	1.1472	H → 1-L	0.14932 (4.4%)	510.1	0.2578	H-1→ L+1	0.10103 (2.0%)
7CPP_2R	331.3	2.1849	H → L+2	0.56137 (63.0%)	516.2	0.3017	H → L	0.69261 (95.5%)
[7CPP_2R. GA]	348.4	1.9991	H → L+2	0.57313 (65.7%)	508.6	0.2343	H → L	0.68529 (93.9%)
7CPP_3R	301.4	3.4000	H-5 → L+1	0.27631 (15.2%)	517.9	0.2896	H → L	0.69222 (95.8%)

[7CPP_3R. GA]								
TET_7R1	286.1	0.905	H → L+2	0.18439 (6.8%)	533.8	0.1485	H → L	0.70733 (100.0%)
[TET_7R1. GF]	287.0	0.6796	H -1 → L+2	0.33326 (22.2%)	533.7	0.1559	H → L	0.70729 (100.0%)
[TET_7R1. GD]	288.2	0.7877	H-1 → L+2	0.37007 (27.4%)	532.4	0.152	H → L	0.70752 (100.1%)
[TET_7R1. GB]	286.9	0.9214	H-1 → L+2	0.36548 (26.7%)	534.4	0.1522	H → L	0.70705 (99.9%)
[TET_7R1. GA]	290.4	0.5673	H-2 → L+1	0.30655 (18.9%)	533.1	0.1524	H → L	0.70772 (100.1%)
TET_7R2	331.2	1.6152	H → L+4	0.28486 (16.2%)	556.3	0.3415	H → L	0.70334 (98.9%)
[TET_7R2. GD]	330.7	1.4119	H-6 → L	0.24776 (12.3%)	551.2	0.3478	H → L	0.70351 (98.9%)
[TET_7R2. GF]	331.7	1.437	H-2 → L	0.51780 (53.6%)	553.7	0.3583	H → L	0.70329 (98.9%)

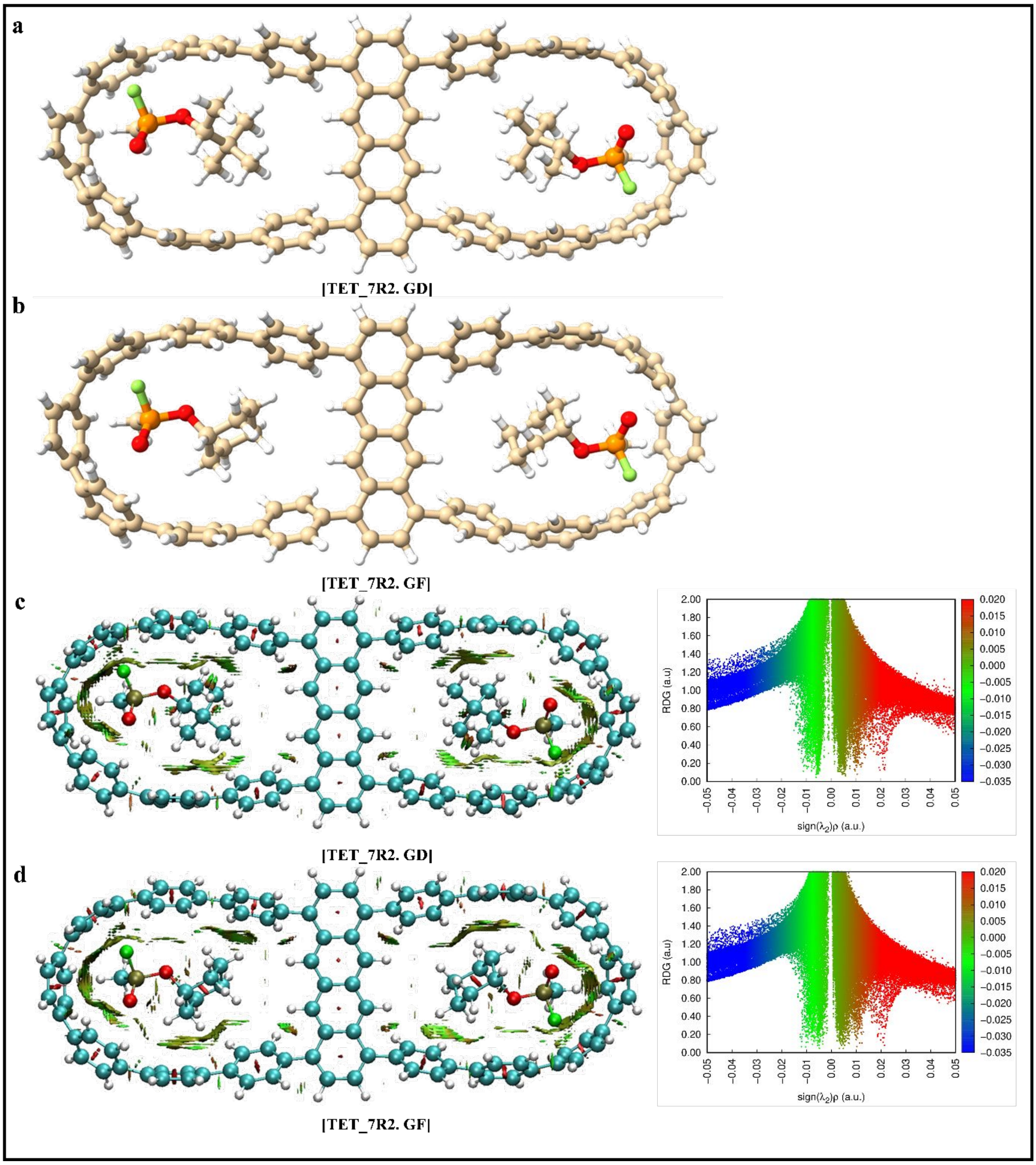

Figure S6. The optimized encapsulated complex structures between the double-hooped **TET_7R2** host with G-series nerve agent Soman & Cyclosarin employing the M06-2X-D3/6-31G+(d) level of theory in gas phase. **a & b**) Optimized complex geometries of [**TET_7R2.**

GD] & [**TET_7R2. GF]** complex systems. **c & d**) The reduced density gradient (RDG) isosurface analysis and NCI scatter diagram visualized the non-covalent interactions of these G-series nerve agent encapsulated complex system with the supramolecular double-hooped **TET_7R2** hosts. In the host, carbons and hydrogens are depicted in brown and white colors, whereas in nerve agents, green, red, orange, brown, and white colors signify the fluorine, oxygen, phosphorous, carbon, and hydrogen atoms, respectively, are shown in ball and stick model.

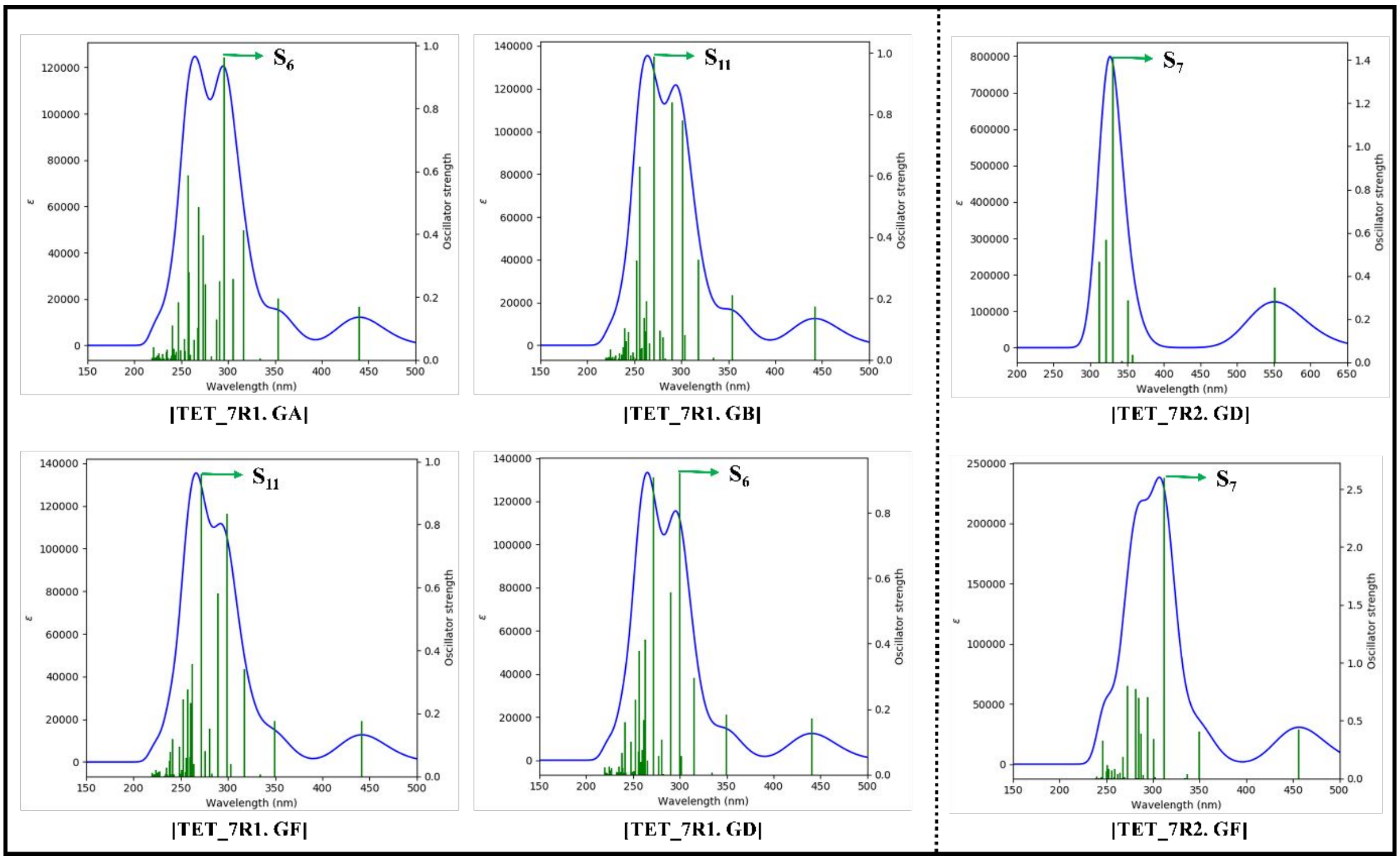

Figure S7. The UV-Vis line spectra of the studied complex systems between the supramolecular tetracene-based **TET_7R1**, and **TET_7R2** hosts with G-series nerve agents using the M06-2X-D3/6-31+G(d) level of theory in the gas phase. Only Soman and Cyclosarin encapsulated on **TET_7R2** tetracene-based supramolecular host is showed in right side of black the dotted line.

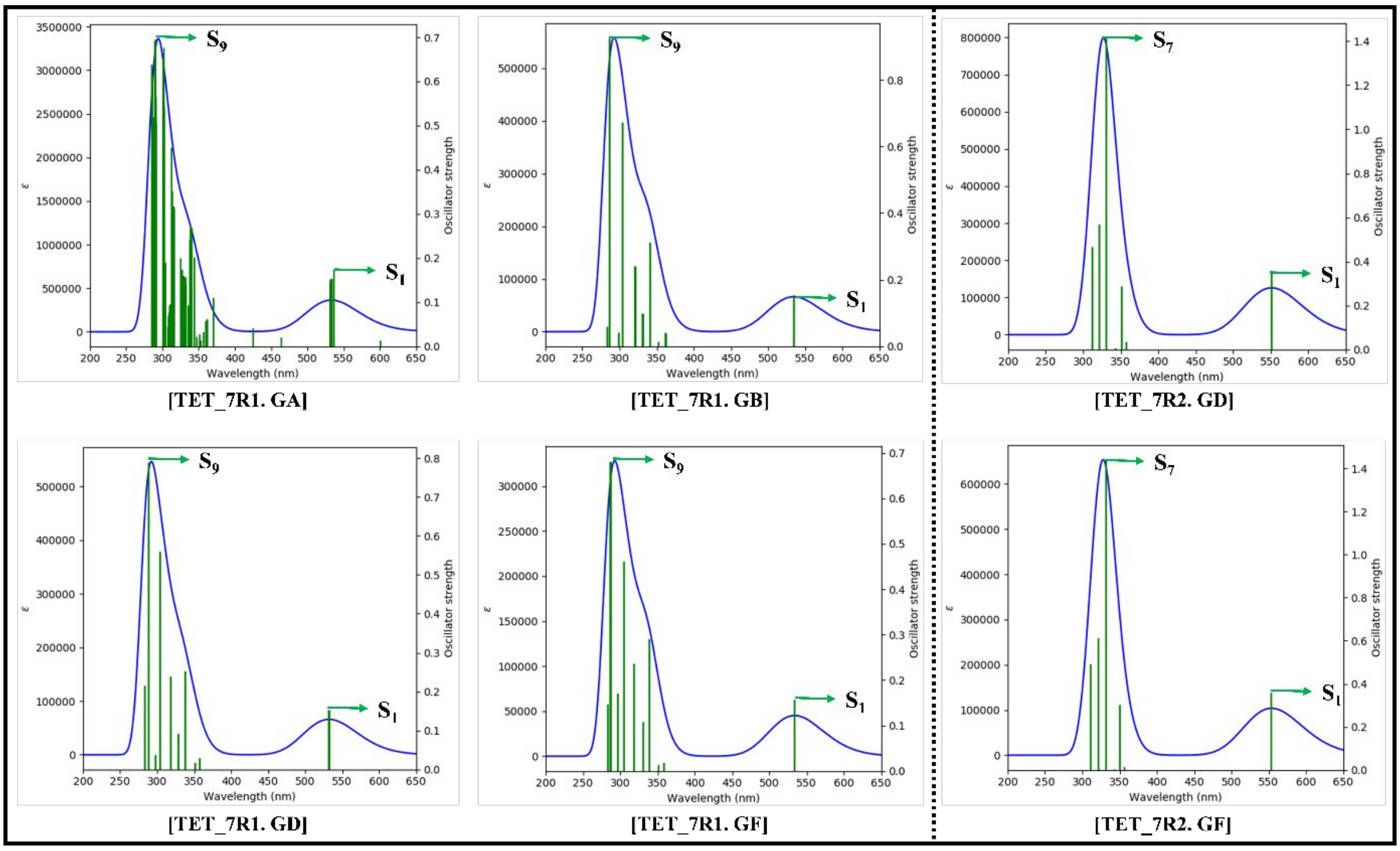

Figure S8. The emission line spectra of the studied complex systems between the supramolecular tetracene-based **TET_7R1**, and **TET_7R2** hosts with G-series nerve agents using the M06-2X-D3/6-31+G(d) level of theory in the gas phase. Only Soman and Cyclosarin encapsulated on **TET_7R2** tetracene-based supramolecular host is showed in right side of black the dotted line.

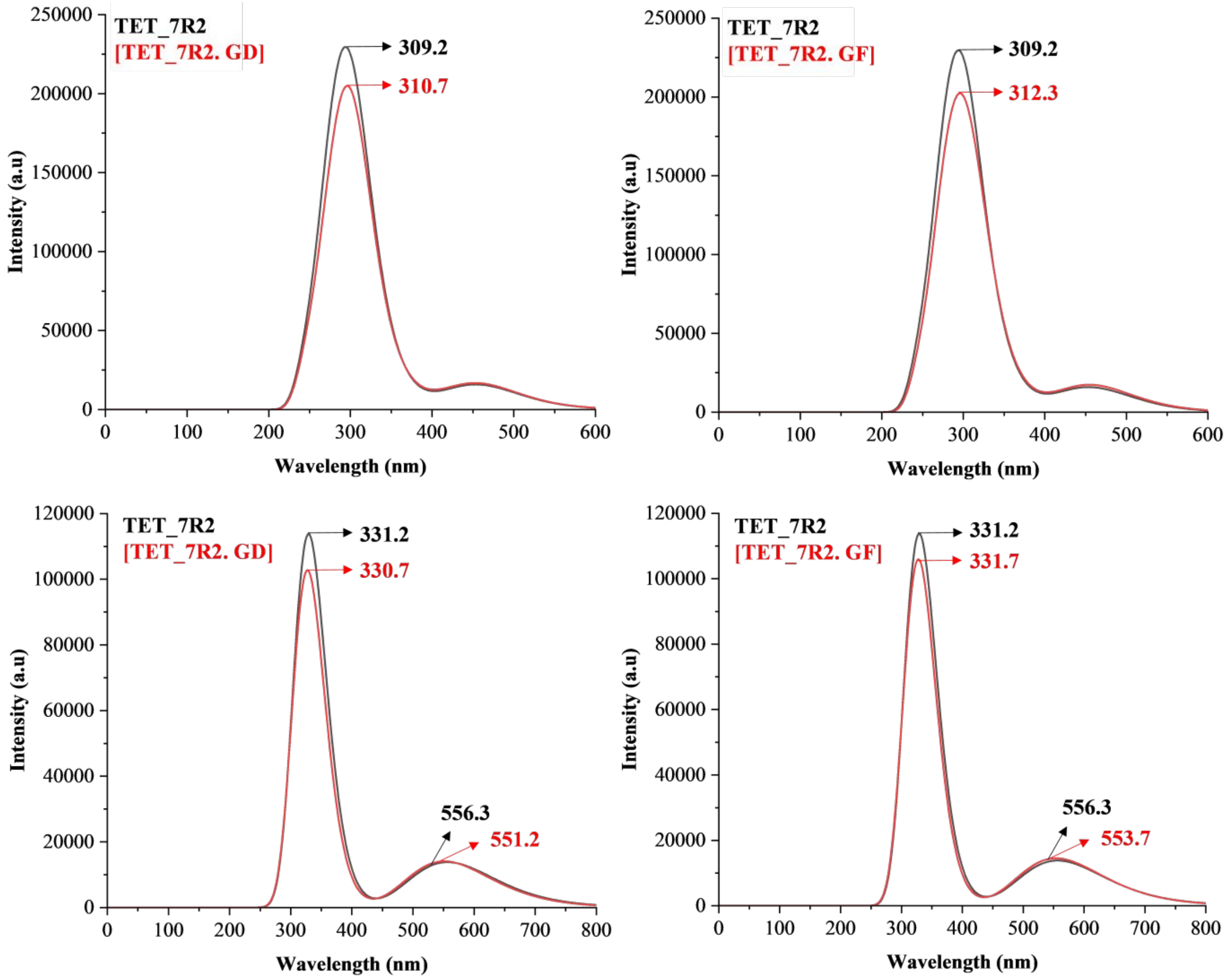

Figure S9. The above UV-Vis and the below emission spectrum of the G-series nerve agents Soman & Cyclosarin encapsulated complex system with supramolecular double-hooped **TET_7R2** host employing the M06-2X-D3/6-31G+(d) level of theory in gas phase.

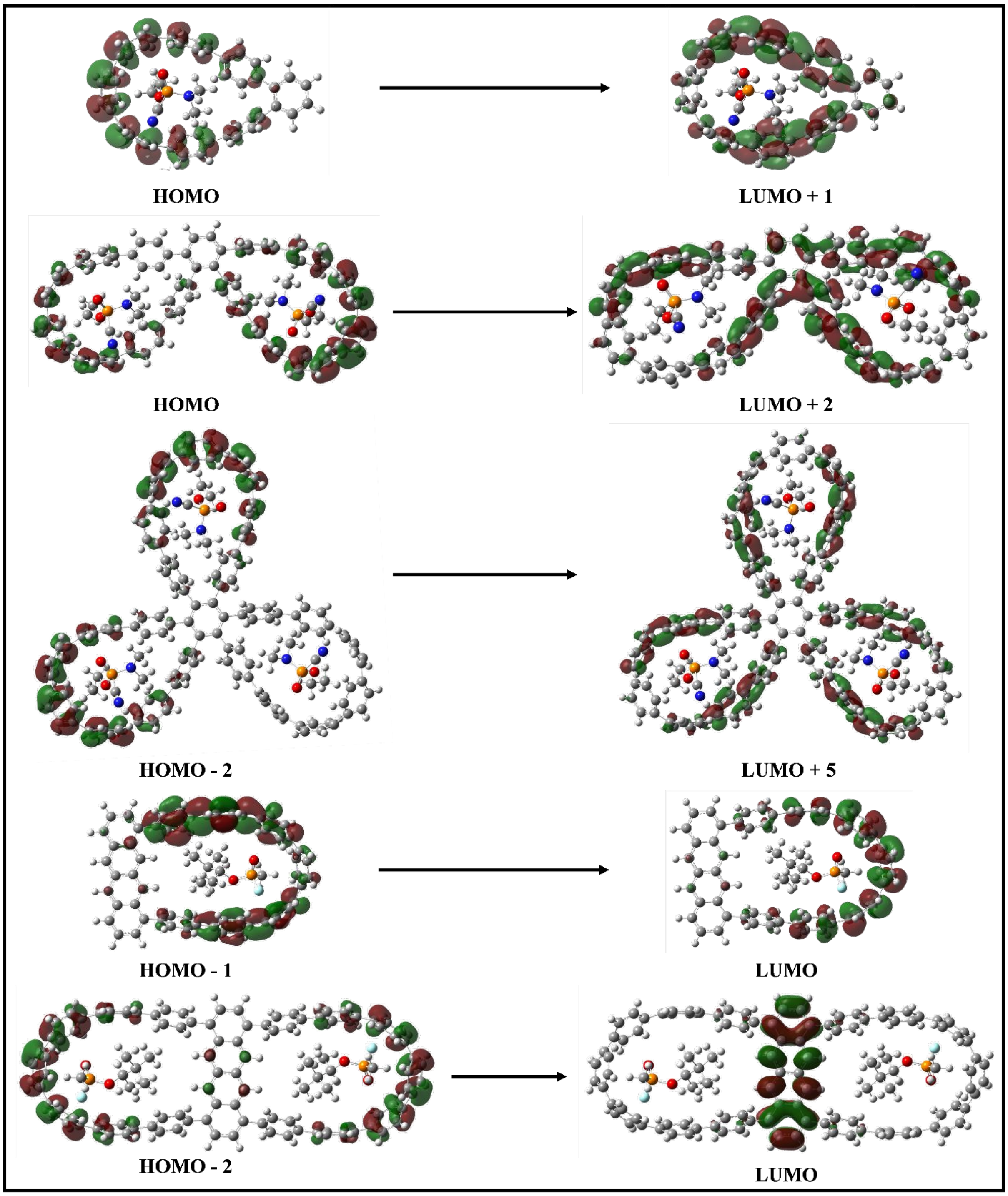

Figure S10. HOMO and LUMO isosurfaces and their corresponding transitions of these **[7CPP_1R. GA]**, **[7CPP_2R. GA], [7CPP_3R. GA], [TET_7R1. GD],** and **[TET_7R2. GD]** complex systems.

Table S7. The strain energy of the encapsulated complex system. All energy is given in kcal/mol.

[7CPP_1R. GA]	2.3	[TET_7R1. GD]	1.1
[7CPP_2R. GA]	5.3	[TET_7R2. GD]	2.4
[7CPP_3R. GA]	8.0	[TET_7R2. GF]	2.5

The Cartesian coordinates of the optimized individual nerve agents, supramolecular hosts, and their encapsulated complex systems at the M06-2X-D3/6-31+G(d) level of theory in the gas phase. The corresponding electronic energies are given in Hartree.

Sarin
E = -749.988110

P	-1.03587200	0.10873600	-0.09370800
O	-1.06495500	1.37157700	-0.84931200
O	0.36595700	-0.32086300	0.52787800
C	-2.18133400	-0.10497300	1.26606600
H	-3.19965400	-0.00064800	0.88489200
H	-2.05293600	-1.09184400	1.71502200
H	-1.99609300	0.66821100	2.01445000
F	-1.38692500	-1.14234700	-1.01805700
C	1.59322900	-0.03641200	-0.20797600
H	1.35506800	-0.02941300	-1.27837600
C	2.54662000	-1.17237800	0.10620800
H	3.49314800	-1.02289300	-0.42237000
H	2.74922700	-1.20505900	1.18156200
H	2.12078100	-2.13164300	-0.19968700
C	2.11032500	1.32585100	0.21597000
H	1.37598700	2.10198900	-0.01478900
H	2.31358500	1.32899900	1.29211000
H	3.04024100	1.55414100	-0.31482100

Soman
E = -867.873978

P	1.77244000	-0.07951100	-0.17269900
O	1.76021800	-0.30561900	-1.62713000
O	0.35861700	0.14200700	0.52362800
C	2.57045400	-1.29443100	0.87398000
H	2.08582300	-2.26230700	0.72821600
H	3.62215000	-1.37224100	0.58902800
H	2.49210200	-0.99596900	1.92159100
C	-0.78159400	0.64225600	-0.23310400
C	-1.95276000	-0.33022900	-0.00218500
F	2.55823700	1.24857600	0.22409800
C	-2.36310900	-0.37090000	1.47344300
H	-1.50454400	-0.60362100	2.11232200
H	-2.79211700	0.58128400	1.80388200
H	-3.12245800	-1.14591700	1.62751000
C	-1.51560600	-1.73115800	-0.45131100
H	-1.12419700	-1.71626100	-1.47566400
H	-0.73627600	-2.13116700	0.20557200
H	-2.36612400	-2.42115000	-0.41935600
C	-3.14098300	0.11879800	-0.86264000
H	-3.95188200	-0.61385600	-0.78645400
H	-3.53940100	1.08540900	-0.53772400
H	-2.86030700	0.19831600	-1.92006200
C	-1.00081300	2.08383100	0.19417700
H	-1.19969500	2.14736500	1.26783400
H	-0.10445400	2.67238900	-0.02245500
H	-1.83785800	2.53015700	-0.34968600
H	-0.51570900	0.60294300	-1.29709600

7CPP_7R1

Tabun
E = -798.265008

P	-0.07503600	0.25439600	0.36054500
O	1.05126500	0.33224100	-0.76336100
O	0.33033500	0.21948300	1.77667600
C	2.13838100	-0.62685700	-0.71785300
H	1.75589000	-1.58688400	-0.35294900
H	2.44280200	-0.74772500	-1.75945700
C	3.26772900	-0.10434500	0.14648500
H	2.93233200	0.02040300	1.17925000
H	4.10340500	-0.81123200	0.12763600
H	3.61867800	0.85979700	-0.23108900
N	-1.06163200	-0.99271500	-0.09070200
C	-1.57359300	-1.04617200	-1.45800500
H	-2.50804400	-0.47605000	-1.55693500
H	-0.83602600	-0.64152100	-2.15398900
H	-1.76724400	-2.09035100	-1.72223500
C	-1.95997400	-1.56537300	0.91180700
H	-2.11166100	-2.62486200	0.68304000
H	-1.50871200	-1.47550800	1.90049900
H	-2.93545500	-1.05977600	0.90754300
C	-0.94989800	1.77055100	-0.09521200
N	-1.60252500	2.68373600	-0.37491800

Cyclosarin
E = -866.677921

P	1.81795900	-0.10718500	0.00264600
O	2.05093400	-1.20982900	-0.94405000
O	0.30484800	0.31874200	0.25142200
F	2.46720900	1.25725300	-0.50597300
C	2.48832600	-0.24446200	1.65826000
H	2.24689000	0.64830800	2.23856000
H	2.06193400	-1.12590400	2.14179800
H	3.57238000	-0.36094700	1.59021200
C	-2.12729500	-1.37316200	0.37108400
C	-3.16494000	-0.26743700	0.58560700
C	-2.49252000	1.10656900	0.65597900
C	-1.66052200	1.37734000	-0.60077300
C	-0.64861100	0.26757500	-0.85437900
C	-1.29560800	-1.11289700	-0.88781000
H	-3.24198200	1.89617000	0.77974500
H	-3.88345900	-0.27815800	-0.24741300
H	-3.73801700	-0.45632300	1.50057100
H	-1.46037900	-1.41672600	1.24236400
H	-2.61550600	-2.35094100	0.29456100
H	-2.31987300	1.42955100	-1.47800500
H	-1.13593600	2.33623000	-0.52852400
H	-0.10163700	0.45473700	-1.78576200
H	-1.94379000	-1.15178800	-1.77448100
H	-0.51659800	-1.87036300	-1.02220700
H	-1.83751600	1.14618500	1.53587000

E = -1847.653862

C	7.57667900	1.38684200	-0.34091300
C	8.72240700	-0.72875000	-0.42284600
C	6.30112300	-0.66978300	-0.17838600
C	8.76037800	0.66295900	-0.46097300
C	7.50110000	-1.38275900	-0.28736700
C	6.34560900	0.74225800	-0.18617200
C	5.01622000	-1.42342100	-0.11789400
C	4.82609100	-2.48072100	0.77950700
H	5.62575300	-2.74538700	1.46782600
C	3.62218200	-3.18425400	0.82092200
C	2.56859700	-2.85479900	-0.03931000
C	2.77122500	-1.80685100	-0.95021500
C	3.96950700	-1.10961100	-0.99268500
H	4.09120600	-0.29843500	-1.70464800
C	1.22733100	-3.48891300	0.02730900
C	0.45657300	-3.63587200	-1.13306100
H	0.90985700	-3.45747000	-2.10450700
C	-0.89959000	-3.92471600	-1.06084700
H	-1.48405700	-3.91367700	-1.97603000
C	-1.54233000	-4.09946700	0.17338200
C	-0.73400800	-4.12009100	1.31994900
H	-1.18087100	-4.30550400	2.29320500
C	0.62168100	-3.81137700	1.24910400
H	1.19573500	-3.73753200	2.16960800
C	-3.02031100	-3.94370900	0.23751100
C	-3.58045500	-3.24328200	1.31291100
H	-2.99054700	-3.05578000	2.20509500
C	-4.80025400	-2.59095600	1.18223800
H	-5.11248500	-1.91187200	1.97045300
C	-5.50160900	-2.60632500	-0.03177400
C	-5.06684700	-3.51509700	-1.00936900
H	-5.62991300	-3.62537500	-1.93334300
C	-3.84958200	-4.17494800	-0.87316800
H	-3.49817200	-4.80265300	-1.68812900
C	-6.28705300	-1.38295500	-0.35020500
C	-5.95634600	-0.72686900	-1.54318500
H	-5.48937200	-1.28357200	-2.35084900
C	-5.95178600	0.66103700	-1.61069800
H	-5.48288800	1.12279300	-2.47343500
C	-6.28470600	1.43662400	-0.49084000
C	-6.94497600	0.76990400	0.55812800
H	-7.34434000	1.33464800	1.39661900
C	-6.93729300	-0.61874700	0.63296300
H	-7.31942200	-1.10192600	1.52933300
C	-5.51278900	2.69355500	-0.26400800
C	-5.08846600	2.97272700	1.04525900
H	-5.62481000	2.55362400	1.89090400
C	-3.87257400	3.59757600	1.28535600
H	-3.51295000	3.68384100	2.30765400
C	-3.02818600	3.94898100	0.22371500
C	-3.58165800	3.93559000	-1.06380700
H	-2.98080300	4.25863200	-1.91074800
C	-4.81480800	3.33320000	-1.30176800
H	-5.15430300	3.23299800	-2.32894900
C	-1.55450500	3.94810700	0.41596800
C	-0.63950800	4.72846700	-0.30164200
H	-0.99643700	5.55704000	-0.90914100
C	0.72538600	4.44046900	-0.27032500

Parathion
E = -1559.506363

S	-1.53431100	0.04679600	2.10351500
P	-1.74157300	-0.00100700	0.19290100
O	-0.53543700	-0.69558000	-0.64509100
O	-2.98563500	-0.80586900	-0.39340800
O	-1.86353900	1.39237200	-0.57355500
O	5.35638600	1.12170200	0.39735500
O	5.67273600	-0.93040600	-0.19697000
N	4.95727800	0.02819400	0.03692000
C	0.80381200	-0.47099100	-0.43411600
C	-3.32103100	-2.11175100	0.12141200
C	-2.86655700	2.34760000	-0.16246500
C	1.29990300	0.77327900	-0.04557200
C	1.65354800	-1.54975800	-0.67494100
C	3.50803700	-0.14745300	-0.12886600
C	2.67045900	0.93400200	0.11410800
C	3.02345900	-1.38925600	-0.52474400
C	-4.55684400	-2.58162400	-0.61316000
C	-2.67914200	3.59139200	-1.00194700
H	-2.47028200	-2.78027200	-0.05098600
H	-3.49154700	-2.02602900	1.19913800
H	-3.85283200	1.89849200	-0.32001800
H	-2.73364400	2.55229800	0.90508400
H	0.62361200	1.60328600	0.12703900
H	1.22683500	-2.49946000	-0.97891900
H	3.09295000	1.88371700	0.42060300
H	3.71336000	-2.20551300	-0.70394000
H	-4.85128600	-3.57003600	-0.24821500
H	-5.38529200	-1.88777700	-0.44689800
H	-4.36496100	-2.64708700	-1.68730300
H	-3.42507900	4.34112000	-0.72163700
H	-1.68391300	4.01535500	-0.84311100
H	-2.79625600	3.36053300	-2.06392600

Water
E = -76.383748

O	0.00000000	0.00000000	0.11646300
H	0.00000000	0.77057700	-0.46585200
H	0.00000000	-0.77057700	-0.46585200

[6CPP. Water]
E = -1693.0779

C	5.39189200	-0.69732600	0.00249800
C	6.57286200	1.41548500	-0.07543900
C	7.82330000	-0.63904300	0.04259600
C	5.36255700	0.71367700	-0.04619200
C	7.79573800	0.75144700	-0.03466300
C	6.62703900	-1.35149200	0.05834400
C	4.13901600	-1.50361900	-0.04278900
C	3.81022000	-2.39557500	0.98326500
H	4.49967500	-2.52770900	1.81420200
C	2.58849700	-3.06709800	0.98398100
C	1.67312300	-2.88335400	-0.05789700
C	2.03829800	-2.04501300	-1.12114500

H	1.40817500	5.06179300	-0.84442700
C	1.22510400	3.35356700	0.46258400
C	0.31319000	2.62045300	1.23160300
H	0.64763800	1.74709500	1.78301900
C	-1.04022900	2.91508700	1.21380900
H	-1.72562300	2.26447600	1.74869300
C	2.62574300	2.86743700	0.35759600
C	3.20299400	2.12475800	1.39879200
C	4.41207100	1.46534300	1.22580900
H	4.81541700	0.85363600	2.02926400
C	5.11058000	1.55308300	0.01742500
C	4.59324500	2.37603700	-0.98651700
H	5.12490300	2.46384800	-1.93164600
C	3.36171400	3.00986600	-0.82510600
H	2.68997600	2.04163200	2.35241700
H	2.94140800	3.56855800	-1.65729500
H	3.50508400	-4.00074200	1.52939900
H	1.96119100	-1.50549900	-1.60825000
H	7.46456500	-2.46941500	-0.29846700
H	9.63861100	-1.30526100	-0.51250000
H	9.70678100	1.18358800	-0.57419700
H	7.59953400	2.47396100	-0.33949000

[7CPP_7R1. GB]
E = -2597.673556

C	-7.86395000	1.04451400	0.19402700
C	-8.86814600	-1.13867200	0.33764400
C	-6.47728000	-0.92867900	-0.06585900
C	-8.98975400	0.24780100	0.38768700
C	-7.62037600	-1.71567200	0.11718600
C	-6.60928400	0.47697300	-0.04610700
C	-5.15278300	-1.59741200	-0.21056200
C	-4.94447600	-2.62551300	-1.13705100
H	-5.75877500	-2.92312100	-1.79399100
C	-3.70797100	-3.26409900	-1.23976300
C	-2.64165400	-2.89601100	-0.41233800
C	-2.85591200	-1.86917600	0.51962200
C	-4.08605800	-1.23681700	0.62202900
H	-4.22523200	-0.44724300	1.35572200
C	-1.28346300	-3.48680600	-0.50853200
C	-0.50449700	-3.63847900	0.64551200
H	-0.95035900	-3.47154600	1.62229300
C	0.85129200	-3.92241900	0.56366500
H	1.43387300	-3.92304500	1.47834300
C	1.49077800	-4.07455300	-0.67591000
C	0.67857900	-4.07486800	-1.82073100
H	1.12058200	-4.23993800	-2.79970800
C	-0.68048700	-3.78101100	-1.73870300
H	-1.26124700	-3.70172400	-2.65471500
C	2.96855500	-3.91305900	-0.73529800
C	3.54470900	-3.24953800	-1.82627900
H	2.97734700	-3.11058200	-2.74188200
C	4.74774100	-2.56617800	-1.69077600
H	5.06415800	-1.91182700	-2.49812400
C	5.41497200	-2.51473000	-0.45715100
C	4.97962200	-3.40325100	0.53859400
H	5.51657500	-3.46408200	1.48206200
C	3.77988600	-4.09137400	0.39854300
H	3.42253000	-4.68921700	1.23238700
C	3.24595800	-1.36014100	-1.10998100
H	3.48547000	-0.67167100	-1.91670400
C	0.27326400	-3.37654600	-0.00218800
C	-0.38826500	-3.86589900	-1.13795000
H	0.18985200	-4.12219500	-2.02257100
C	-1.77612300	-3.95351900	-1.17683500
H	-2.26249800	-4.27301000	-2.09536100
C	-2.55347900	-3.53086600	-0.08743200
C	-1.87954600	-3.21623000	1.10296700
H	-2.44582800	-2.87894200	1.96678700
C	-0.49062800	-3.14661300	1.14758600
H	-0.00650800	-2.77557800	2.04800400
C	-3.93888400	-3.01168200	-0.24616300
C	-4.80143700	-2.80838300	0.84453600
H	-4.70526700	-3.42641800	1.73413900
C	-5.66025300	-1.71471500	0.87217700
H	-6.20675900	-1.49830800	1.78679400
C	-5.66106600	-0.78456500	-0.18220600
C	-5.07324700	-1.19919600	-1.38649500
H	-5.08701800	-0.54043200	-2.24943000
C	-4.22320600	-2.29779600	-1.41761400
H	-3.62096700	-2.45581900	-2.30773000
C	-5.67266400	0.68688900	0.06380600
C	-5.08681600	1.10142000	1.26798700
H	-5.07294200	0.43272800	2.12265400
C	-4.25688700	2.21461800	1.30691000
H	-3.64270800	2.35745000	2.19089400
C	-3.99264500	2.94622700	0.14265200
C	-4.85285700	2.73462200	-0.94979500
H	-4.77368600	3.36430800	-1.83295000
C	-5.68817000	1.62301900	-0.98623800
H	-6.23358100	1.40416000	-1.90114600
C	-2.61602700	3.49256200	-0.00864700
C	-1.93723200	3.22638900	-1.20906000
H	-2.50100100	2.92108000	-2.08606700
C	-0.54707700	3.16331900	-1.25224600
H	-0.05701500	2.83104700	-2.16431300
C	0.21160600	3.34496300	-0.09074900
C	-0.45370300	3.79476200	1.05888100
H	0.11951500	4.00258500	1.95878600
C	-1.84115200	3.88258400	1.09596900
H	-2.32944400	4.15838000	2.02692300
C	1.60477200	2.83355400	-0.03362300
C	1.92566200	1.92869500	0.98920800
C	3.14247500	1.25971400	0.98961400
H	3.35526800	0.53227100	1.76889900
C	4.08103900	1.47515800	-0.02620900
C	3.78133500	2.41464600	-1.01906800
H	4.50265100	2.59925400	-1.81214700
C	2.55521800	3.07889800	-1.02986300
H	1.17794100	1.69281600	1.74250900
H	2.33083000	3.78511500	-1.82614800
H	2.33474900	-3.72496200	1.81185700
H	1.33325700	-1.87657000	-1.93138400
H	6.54492000	2.50222100	-0.10075900
H	8.72146100	1.31924600	-0.05042300
H	8.77072500	-1.16882200	0.08009700
H	6.64106100	-2.43848000	0.08620600
O	-1.23030900	0.52587500	1.29474400
H	-1.48310600	1.18977700	0.63432100

C	6.13203000	-1.24825600	-0.14481600
C	5.74527800	-0.59553500	1.03408500
H	5.30528900	-1.16320300	1.85086500
C	5.62999300	0.78986200	1.07486600
H	5.09603200	1.21600200	1.91769200
C	5.90831600	1.55853600	-0.06289100
C	6.63117300	0.92730600	-1.09340200
H	6.99349800	1.50559800	-1.93950500
C	6.73485600	-0.45777600	-1.13887200
H	7.16204800	-0.92809000	-2.02158200
C	5.04861900	2.74835200	-0.32206000
C	4.62424300	2.97990500	-1.64159800
H	5.18470400	2.56300900	-2.47263500
C	3.39532100	3.56661800	-1.90753800
H	3.04547000	3.62569500	-2.93527900
C	2.53927000	3.93764700	-0.86185500
C	3.07350300	3.94571100	0.43378600
H	2.46018700	4.27644900	1.26866500
C	4.31262900	3.36843600	0.69962000
H	4.62809100	3.27663900	1.73450800
C	1.06830400	3.95040900	-1.06797500
C	0.16217100	4.74743400	-0.35699100
H	0.52973200	5.57919700	0.23989400
C	-1.20253000	4.45925600	-0.36498000
H	-1.87786900	5.07855300	0.22069600
C	-1.70716900	3.35392600	-1.06491400
C	-0.81154100	2.62390300	-1.85620900
H	-1.15662600	1.74510200	-2.39304600
C	0.54216000	2.92030200	-1.86226400
H	1.21894800	2.27231500	-2.41218700
C	-3.07489000	2.81268300	-0.86405600
C	-3.70310900	2.06241000	-1.86887700
C	-4.85562300	1.33621100	-1.60388300
H	-5.29983400	0.72028700	-2.38208100
C	-5.44208800	1.35930200	-0.33420000
C	-4.87775200	2.18348800	0.64253900
H	-5.32320600	2.21872600	1.63465800
C	-3.70111800	2.88660200	0.38654800
H	-3.26912500	2.02508000	-2.86418000
H	-3.22916800	3.44512900	1.19173400
H	-3.57774600	-4.06459900	-1.96402100
H	-2.03217000	-1.54464900	1.15005400
H	-7.51557600	-2.79790300	0.11805200
H	-9.73869600	-1.77150600	0.48449700
H	-9.95692600	0.70864200	0.56579800
H	-7.95421300	2.12808800	0.20113000
P	2.02349800	-0.26504300	1.82985200
O	2.82622900	0.72779800	2.57090900
O	0.44308700	-0.18283900	2.05678300
C	2.25958500	-0.38759000	0.06071600
H	3.27976100	-0.71918700	-0.15634700
H	1.54071500	-1.09777200	-0.36036000
H	2.10264600	0.60808000	-0.37015600
F	2.30444900	-1.75234600	2.31576200
C	-0.13738300	0.96248400	2.74352500
H	0.41587500	1.09991900	3.67849000
C	-1.58345900	0.60148200	3.02345700
H	-2.04508600	1.37943100	3.64016300
H	-2.14101800	0.53646000	2.08228400
H	-1.65639500	-0.35361200	3.55185800
H	-1.25163200	-0.32439100	0.83116000

[6CPP. Parathion]
E = -3176.222939

C	4.14111100	-1.45064300	2.09182700
C	5.32595800	-3.04897400	0.70390700
C	6.55665800	-1.72776900	2.29350800
C	4.12315800	-2.43835600	1.07968500
C	6.53557900	-2.70562600	1.30170900
C	5.36958800	-1.11122300	2.67697000
C	2.92805300	-0.69450900	2.52649200
C	2.88795600	0.70011500	2.39260600
H	3.74853100	1.21884400	1.97540900
C	1.73874800	1.42317800	2.70558800
C	0.58507100	0.77878100	3.17137200
C	0.65906100	-0.60575100	3.38930800
C	1.80705200	-1.32799400	3.07492100
H	1.81876400	-2.40289900	3.22602000
C	-0.74273200	1.44594100	3.23143200
C	-1.73093700	1.05841600	4.14994500
H	-1.45451700	0.45291500	5.01001400
C	-3.06893300	1.37342700	3.94315900
H	-3.80886400	1.00347000	4.64757200
C	-3.48335900	2.06324300	2.79002100
C	-2.47316800	2.61436600	1.98749200
H	-2.73952100	3.16603100	1.08859500
C	-1.13016300	2.31760600	2.20654600
H	-0.39490900	2.65341900	1.47707800
C	-4.85252500	1.90697500	2.22509300
C	-5.32497800	2.67934600	1.15191500
H	-4.90749500	3.66591100	0.96587400
C	-6.19240300	2.13727300	0.20720400
H	-6.40322400	2.71500100	-0.68845400
C	-6.60334200	0.79714100	0.29619400
C	-6.39950100	0.16987000	1.53721400
H	-6.73750100	-0.84919400	1.69268800
C	-5.53801700	0.71014800	2.47986800
H	-5.25605400	0.08805700	3.32367700
C	-6.66405400	-0.09961800	-0.89827000
C	-5.91296700	0.27360400	-2.02154600
H	-5.74288500	1.32160900	-2.23867200
C	-5.09023600	-0.65333200	-2.65943800
H	-4.33250900	-0.27747300	-3.34372800
C	-5.02786100	-1.97276700	-2.20038700
C	-6.09287600	-2.40383500	-1.38605200
H	-6.18409300	-3.45638800	-1.12768700
C	-6.90885000	-1.48003900	-0.75016800
H	-7.61686900	-1.83541000	-0.00593700
C	-3.70710500	-2.65496900	-2.15231600
C	-3.34600600	-3.20514900	-0.91284800
H	-4.09538200	-3.30040800	-0.13347900
C	-2.02396300	-3.47225400	-0.59571100
H	-1.79676200	-3.78173600	0.41951200
C	-0.98645400	-3.21609100	-1.50215200
C	-1.35622400	-2.78154800	-2.78505000
H	-0.59280000	-2.61772500	-3.54114400
C	-2.68596400	-2.50367300	-3.10335500
H	-2.92228200	-2.11633000	-4.09174300
C	0.41794300	-3.23611500	-1.00929200

C	-0.01468600	2.19177400	1.86277700
H	1.03415700	2.45707400	1.69604300
H	-0.49285600	2.00241600	0.89519300
H	-0.51400900	3.04693000	2.33160600

[7CPP_7R1. GD]
E = -2715.563834

C	7.99253100	-0.96781700	-0.33851700
C	8.96487700	1.19015900	0.09288200
C	6.56436200	0.99468200	-0.27272100
C	9.11146000	-0.18562200	-0.06547400
C	7.70178600	1.76720100	-0.00621400
C	6.72136500	-0.39800800	-0.45774400
C	5.22966900	1.66097500	-0.30283500
C	5.02429700	2.84518600	-1.02144100
H	5.84765300	3.27353500	-1.58876400
C	3.77912800	3.47418800	-1.04133500
C	2.69513700	2.93865400	-0.33807100
C	2.90669600	1.75768100	0.38908000
C	4.14557500	1.13518800	0.41263800
H	4.26810500	0.21505100	0.97817900
C	1.32686500	3.51333800	-0.37721600
C	0.49930800	3.42554900	0.74903800
H	0.90976500	3.07193600	1.69054200
C	-0.85733400	3.70777700	0.66832300
H	-1.48576300	3.52003900	1.53392700
C	-1.44396200	4.10289500	-0.54286100
C	-0.59090900	4.32418200	-1.63423700
H	-1.00258500	4.67166900	-2.57859400
C	0.76809400	4.03035500	-1.55417400
H	1.38651600	4.13067600	-2.44310900
C	-2.91728400	3.96919600	-0.68462900
C	-3.42683700	3.37782700	-1.84757900
H	-2.79555600	3.28442800	-2.72725000
C	-4.64618100	2.71289500	-1.83087800
H	-4.91830100	2.11211800	-2.69374100
C	-5.39949600	2.61353900	-0.64987300
C	-5.01426500	3.43170300	0.42338000
H	-5.61229600	3.45003400	1.33102000
C	-3.79239300	4.09516500	0.40617500
H	-3.46964300	4.62779200	1.29726100
C	-6.17956700	1.35870600	-0.46597900
C	-5.95440000	0.64015000	0.71635600
H	-5.61150300	1.15562500	1.60980900
C	-5.87239900	-0.74927800	0.69795400
H	-5.46501500	-1.23075700	1.57949300
C	-6.00479300	-1.45341500	-0.50657400
C	-6.60521300	-0.75644400	-1.57381200
H	-6.87750600	-1.27863700	-2.48670500
C	-6.68717100	0.63021600	-1.55641500
H	-7.00785600	1.15144400	-2.45541500
C	-5.10295000	-2.61892400	-0.74992900
C	-4.63591800	-2.84057800	-2.05791100
H	-5.18949500	-2.45562700	-2.90814600
C	-3.38054400	-3.38292000	-2.29061400
H	-3.00231100	-3.43225100	-3.30886800
C	-2.53884600	-3.72680000	-1.22354500
C	-3.10207600	-3.74488600	0.05840000
H	-2.49551300	-4.03974900	0.91148400
C	1.40666200	-2.41729800	-1.57025500
C	2.62438600	-2.21292000	-0.92239400
H	3.34712200	-1.52942700	-1.36289400
C	2.88438300	-2.79592700	0.32127900
C	1.95679400	-3.71745400	0.81746900
H	2.17165400	-4.24976500	1.74110500
C	0.75483600	-3.94235400	0.15790600
H	1.20088300	-1.85010100	-2.47487200
H	0.05643900	-4.65946700	0.57809200
H	1.72616700	2.49815500	2.54106900
H	-0.22088100	-1.13820700	3.74149900
H	5.30079300	-3.79680200	-0.08506300
H	7.45377900	-3.19638300	0.99123400
H	7.49205100	-1.44456800	2.76818900
H	5.38081700	-0.34908300	3.45273400
S	3.58786600	0.74824700	-3.40955100
P	3.65117000	1.61820300	-1.69132700
O	2.58338600	1.09767900	-0.59393900
O	5.00512400	1.52070000	-0.86196500
O	3.37990500	3.19174300	-1.65549100
O	-3.53476800	1.90890100	-1.37013900
O	-3.55005600	0.20008000	-0.04674500
N	-2.97728500	1.01576900	-0.74455900
C	1.21559800	1.04851900	-0.75364500
C	5.77258700	0.29937700	-0.76140500
C	4.15454800	4.07181800	-2.49816700
C	0.52862400	1.94170700	-1.57563700
C	0.54343500	0.09308200	0.00777600
C	-1.51818000	0.96002400	-0.81751900
C	-0.85968600	1.89750700	-1.60231700
C	-0.84081100	0.04476900	-0.02091800
C	7.15376100	0.67329100	-0.27245700
C	3.58517700	5.46468600	-2.34232200
H	5.26322800	-0.36250100	-0.05479300
H	5.80510300	-0.17724300	-1.74728500
H	5.19965400	4.02332700	-2.17436300
H	4.08372400	3.71651800	-3.53175500
H	1.06510400	2.68043100	-2.15943400
H	1.11198300	-0.58458100	0.63423900
H	-1.43133300	2.59472200	-2.20279100
H	-1.39265700	-0.66449700	0.58753400
H	7.76660700	-0.22903900	-0.18921600
H	7.63444400	1.36541000	-0.96989900
H	7.09626300	1.14380200	0.71343000
H	4.15276500	6.16716100	-2.95978700
H	2.53922200	5.48892000	-2.66041100
H	3.64173000	5.78919500	-1.30000200

[7CPP_7R1. GA]
E = -2645.960945

C	7.97933000	-0.95822500	0.16085200
C	8.87073700	1.25932300	0.42482200
C	6.49498100	0.95443000	-0.03196800
C	9.06296900	-0.11972400	0.40689700
C	7.59883100	1.78281200	0.21281300
C	6.70060400	-0.44321800	-0.07122100
C	5.15050600	1.58542200	-0.18037600
C	4.97883800	2.75116000	-0.93866500
H	5.82773400	3.16578400	-1.47746900

C	-4.36733900	-3.20861700	0.29053500
H	-4.69670100	-3.11533700	1.32007800
C	-1.06426200	-3.69837300	-1.40266300
C	-0.14486400	-4.51296900	-0.73212200
H	-0.49920500	-5.37071600	-0.16470400
C	1.21988400	-4.22178000	-0.75372600
H	1.90149900	-4.87767500	-0.21792200
C	1.71720900	-3.09238300	-1.42164000
C	0.79668300	-2.31492100	-2.13733900
H	1.12055600	-1.40877900	-2.63830600
C	-0.55379300	-2.62049500	-2.14167700
H	-1.24188500	-1.95505400	-2.65651500
C	3.11862400	-2.61048000	-1.29753300
C	3.64023200	-1.68818000	-2.21922200
C	4.82607700	-1.01415400	-1.96922100
H	5.17579000	-0.25726300	-2.66699400
C	5.55734400	-1.25986100	-0.80324400
C	5.11926000	-2.27759700	0.04792200
H	5.68419700	-2.50094900	0.95035900
C	3.91409100	-2.93511500	-0.19157200
H	3.09916000	-1.46784600	-3.13379400
H	3.55976100	-3.65984700	0.53611300
H	3.65455700	4.39608800	-1.60464000
H	2.07083900	1.29891000	0.91052400
H	7.58216800	2.83530800	0.15808300
H	9.82834600	1.81263100	0.30888500
H	10.09103100	-0.64734200	0.01682000
H	8.09960500	-2.03932000	-0.49000900
P	-2.41224100	0.05115500	1.54490400
O	-3.17401600	-0.95218200	2.31517100
O	-0.85039000	0.12770600	1.85911100
C	-2.48144100	0.04167900	-0.24047600
H	-2.11205300	-0.92703300	-0.59343100
H	-3.51680000	0.17715900	-0.56783000
H	-1.85658600	0.85093400	-0.63215200
C	-0.33747800	-0.30294300	3.15128400
C	0.51545100	-1.56770700	2.93932400
F	-2.90045000	1.52717200	1.90912500
C	1.71210900	-1.30386500	2.02256700
H	1.39062400	-0.94118600	1.03916100
H	2.41604900	-0.58269000	2.45495400
H	2.25988100	-2.23843400	1.86371600
C	-0.36231300	-2.65189300	2.30156300
H	-1.28548800	-2.80768900	2.87207200
H	-0.63841500	-2.38356600	1.27410200
H	0.18889200	-3.59837800	2.25029500
C	1.00663700	-2.05709300	4.30817900
H	1.51767100	-3.01953800	4.19686400
H	1.71649700	-1.35730400	4.76148500
H	0.17109200	-2.20082200	5.00437400
C	0.37096600	0.89420800	3.76251700
H	1.24535800	1.18499700	3.17226200
H	-0.31649600	1.74487100	3.80232700
H	0.69817800	0.67388900	4.78236400
H	-1.19403300	-0.58247500	3.77776700

[7CPP_2R. GA]
E = -5059.775341

C	-1.45280100	3.99647500	-0.74371200
C	3.74271300	3.39320100	-1.01764000
C	2.62798400	2.88357700	-0.34450600
C	2.79367200	1.70034200	0.38971700
C	4.02725600	1.07352600	0.48406800
H	4.11773100	0.17173700	1.08236000
C	1.28131500	3.50553400	-0.38185400
C	0.46439900	3.43991100	0.75459300
H	0.86703500	3.05329700	1.68735900
C	-0.87732000	3.78570400	0.69637300
H	-1.49705600	3.58856100	1.56675600
C	-1.45912600	4.22947300	-0.50167300
C	-0.61288600	4.42095200	-1.60376400
H	-1.02063100	4.79486100	-2.53989800
C	0.73243000	4.06072800	-1.54595700
H	1.34390700	4.14237300	-2.44166800
C	-2.93897200	4.13215000	-0.60947900
C	-3.49872900	3.55384800	-1.75602100
H	-2.91986300	3.50829200	-2.67509000
C	-4.67405900	2.81216400	-1.67319700
H	-4.96736900	2.20557100	-2.52622900
C	-5.31861300	2.62944900	-0.44160700
C	-4.93098700	3.47308800	0.61321200
H	-5.47063000	3.44237400	1.55628200
C	-3.75790100	4.21107700	0.53058900
H	-3.41012700	4.74757100	1.40938100
C	-5.94229400	1.30260900	-0.18690300
C	-5.50749900	0.63897900	0.97001500
H	-5.05546400	1.20171200	1.78258700
C	-5.39631700	-0.74383600	0.99775900
H	-4.87545200	-1.19118000	1.83828100
C	-5.70818300	-1.51624700	-0.12993000
C	-6.42464600	-0.87292700	-1.15564700
H	-6.79033700	-1.44442800	-2.00498600
C	-6.53468700	0.51520100	-1.18787100
H	-6.97819400	0.99027200	-2.05998100
C	-4.88844100	-2.73850000	-0.36578800
C	-4.46947200	-3.02575300	-1.67458600
H	-5.02134600	-2.62847900	-2.52175500
C	-3.25026000	-3.64511800	-1.91689100
H	-2.90963800	-3.75671100	-2.94328400
C	-2.39458400	-3.97499800	-0.85678600
C	-2.92915900	-3.92631800	0.43804700
H	-2.32066800	-4.21445200	1.29184400
C	-4.16743700	-3.34127400	0.67702700
H	-4.49228400	-3.23009200	1.70708600
C	-0.92352200	-4.02360600	-1.04723800
C	-0.04279100	-4.69090000	-0.18488500
H	-0.43117900	-5.41531200	0.52661600
C	1.32105000	-4.40620500	-0.19028500
H	1.96587000	-4.93316600	0.50791100
C	1.86068100	-3.42915600	-1.04163600
C	0.99460500	-2.85119100	-1.97902700
H	1.35438600	-2.07975300	-2.65194100
C	-0.35896600	-3.15009000	-1.98883700
H	-1.00441500	-2.61056500	-2.67487900
C	3.22972900	-2.87604300	-0.87060600
C	3.83703800	-2.12043100	-1.88519200
C	4.97852000	-1.36973600	-1.63744400
H	5.39203100	-0.73842200	-2.42013200
C	5.58291300	-1.37995300	-0.37781700

C	0.77645800	3.00106400	-0.96780300
C	0.53417600	5.36279300	-0.47882100
C	-0.62897800	2.87535900	-0.99295700
C	1.35809000	4.26104500	-0.71133600
C	-0.84745100	5.23282100	-0.49410300
C	-2.94223400	3.92120500	-0.66008700
C	1.63682600	1.78904000	-1.14529900
C	-3.74246500	4.89676100	-1.26922900
C	1.52461400	0.94822200	-2.25883600
H	-3.27159800	5.67270000	-1.86825800
H	0.81664300	1.19466400	-3.04542300
C	-5.12964800	4.89687800	-1.12057900
C	2.27735300	-0.22014900	-2.36149600
C	-5.76727000	3.91201400	-0.35948900
C	3.18754300	-0.58009300	-1.36056300
C	-4.96738500	2.91887500	0.22530500
C	3.33308400	0.28970900	-0.27006900
C	-3.58680200	2.92939800	0.09225200
C	2.56350400	1.43948600	-0.15548700
H	-3.00105000	2.15620500	0.58009500
H	2.68594200	2.08088700	0.71377400
C	-7.23567300	3.84701600	-0.15843200
C	4.01670900	-1.80957000	-1.41531700
C	-7.74329300	3.36877400	1.05673700
C	4.42196200	-2.43075400	-0.22636600
H	-7.06768400	3.16682200	1.88402400
H	4.03667400	-2.08157100	0.72777700
C	-9.08969400	3.07036900	1.20539500
C	5.37001000	-3.44361200	-0.22944800
H	-9.40779600	2.57589000	2.11863800
H	5.74830400	-3.79416500	0.72617100
C	-9.99325400	3.25136600	0.14615500
C	5.94083600	-3.90096200	-1.42760400
C	-9.50940600	3.85344000	-1.02499000
C	5.43776300	-3.37222700	-2.62613300
H	-10.18551400	4.04156700	-1.85541200
H	5.82597100	-3.72414200	-3.57856800
C	-8.15408700	4.14101700	-1.17610900
C	4.50183300	-2.34058700	-2.61931100
H	-7.79673500	4.53146500	-2.12599600
H	4.19669200	-1.89868700	-3.56449700
C	-11.27205700	2.49553400	0.21155000
C	7.23437800	-4.62888100	-1.33824000
C	-11.69845500	1.78433000	-0.91754500
C	8.26161100	-4.30497900	-2.23409500
H	-11.32170900	2.05458000	-1.90067600
H	8.02563600	-3.81573000	-3.17531000
C	-12.38836400	0.58283200	-0.77903500
C	9.59400500	-4.36142200	-1.83539200
H	-12.51625800	-0.04617400	-1.65620300
H	10.34553600	-3.90400700	-2.47354200
C	-12.66667100	0.06134300	0.49235100
C	9.93364400	-4.74104100	-0.52915800
C	-12.52836100	0.93525700	1.58446500
C	8.93345400	-5.37135400	0.23112900
H	-12.83189900	0.61265600	2.57701300
H	9.17385500	-5.78031600	1.20903200
C	-11.84025900	2.13339800	1.44558500
C	7.60457500	-5.31554300	-0.16805600
H	-11.62813300	2.72443400	2.33230700

C	5.06077000	-2.23203100	0.59896600
H	5.52620300	-2.26616100	1.58173900
C	3.89074900	-2.95012300	0.36373700
H	3.39721200	-2.08700800	-2.87698200
H	3.45465100	-3.51960900	1.18013500
H	3.65299500	4.31123900	-1.59376500
H	1.92570900	1.25897800	0.87447700
H	7.44368700	2.85707500	0.27180900
H	9.70434900	1.92784500	0.61945000
H	10.04912200	-0.54147800	0.57727100
H	8.12055200	-2.03569300	0.12316800
P	-1.54869800	0.03081700	2.10360100
O	-2.10849400	-0.33478600	0.66866100
O	-1.97233300	1.29011700	2.74633800
C	-2.08386600	0.69106600	-0.36192300
H	-2.66980000	1.54409400	-0.00448400
H	-1.04409400	1.00902000	-0.51297900
C	-2.67879800	0.07543400	-1.60538300
H	-3.74280900	-0.13397300	-1.45803500
H	-2.56709900	0.76607800	-2.44675700
H	-2.16788300	-0.86286600	-1.84078600
N	0.08966300	-0.19199800	2.03151700
C	0.61411300	-1.39045200	1.37028200
H	0.66531900	-2.24020300	2.06759800
H	-0.01414700	-1.66910200	0.52026700
H	1.62327300	-1.17765600	0.99826000
C	0.85962500	0.21594200	3.20871900
H	1.89873000	0.38940500	2.91011300
H	0.44794700	1.14064700	3.61683700
H	0.84698600	-0.56344500	3.98448400
C	-2.19346000	-1.42385500	2.98100400
N	-2.52082700	-2.35729900	3.58186400

[7CPP_7R1. GF]
E = -2714.366175

C	8.05655900	-1.10961900	-0.29497100
C	9.12339300	1.04073200	-0.10247100
C	6.70514200	0.90434600	-0.33102400
C	9.21313300	-0.34848200	-0.14147400
C	7.87749700	1.65579400	-0.19200400
C	6.80193200	-0.50342800	-0.40461400
C	5.38774800	1.60088300	-0.33790400
C	5.11760500	2.67883000	-1.18766900
H	5.88641700	3.02275200	-1.87608800
C	3.86946200	3.30172700	-1.18502200
C	2.84570400	2.86671300	-0.33387400
C	3.13675600	1.81148000	0.54291900
C	4.38002600	1.19831600	0.54562200
H	4.57492900	0.38029400	1.23294900
C	1.46193700	3.40481100	-0.39292600
C	0.61243000	3.33805200	0.72044700
H	1.00574100	3.03381400	1.68651100
C	-0.74915800	3.59160000	0.60458800
H	-1.38733900	3.42719900	1.46951100
C	-1.32014200	3.94700100	-0.62665100
C	-0.44780800	4.14985900	-1.70578000
H	-0.84268700	4.47191800	-2.66575100
C	0.91085500	3.87365000	-1.59399200

H	6.83866000	-5.69212100	0.50410900	H	1.53776000	3.95556800	-2.47798400
C	-12.57221000	-1.41423500	0.66043900	C	-2.78964100	3.82401100	-0.80689500
C	11.06743400	-4.02351100	0.11398300	C	-3.28178500	3.27714800	-1.99925100
C	-11.69771500	-1.85561600	1.66477900	H	-2.62720400	3.17761500	-2.86040700
C	10.77810000	-3.40409200	1.33901300	C	-4.52872900	2.66809600	-2.04354100
H	-11.42356700	-1.18841700	2.47760600	H	-4.79140600	2.10376600	-2.93270600
H	9.93581000	-3.74582300	1.93468300	C	-5.33614300	2.57814900	-0.89737800
C	-10.96764000	-3.02535500	1.50910100	C	-4.94717300	3.34590200	0.21281300
C	11.37312900	-2.19883900	1.68416100	H	-5.57336700	3.37318300	1.10062700
H	-10.16711000	-3.21740800	2.21607300	C	-3.69521900	3.95030300	0.25841700
H	10.97232000	-1.66589600	2.54033200	H	-3.38504200	4.44452900	1.17550800
C	-11.08153200	-3.79868500	0.34509300	C	-6.19375100	1.36625600	-0.77219800
C	12.27752400	-1.56589300	0.81934400	C	-6.14794500	0.66441200	0.44022300
C	-12.17213600	-3.51349300	-0.49673800	H	-5.91082600	1.18361100	1.36555700
C	12.78519300	-2.34142400	-0.23912000	C	-6.10311300	-0.72652800	0.45575000
H	-12.37804300	-4.14799200	-1.35485600	H	-5.84093800	-1.19764300	1.39534200
H	13.57209800	-1.94527300	-0.87567000	C	-6.07839300	-1.44732200	-0.74583800
C	-12.90156600	-2.33633800	-0.34695900	C	-6.53573400	-0.75915500	-1.88774200
C	12.18561300	-3.54806700	-0.59128600	H	-6.70093000	-1.28723200	-2.82230600
H	-13.65056400	-2.08010800	-1.09266500	C	-6.59542100	0.62799300	-1.90102600
H	12.51416200	-4.05768000	-1.49408200	H	-6.79884100	1.13458000	-2.84116200
C	-9.85211200	-4.48971900	-0.13606500	C	-5.15048300	-2.61257300	-0.86726800
C	12.26242500	-0.07622600	0.79014600	C	-4.61676600	-2.93062300	-2.12879600
C	-9.57321900	-4.48959300	-1.51248700	H	-5.13853000	-2.63727500	-3.03376300
C	12.35212500	0.57455600	-0.45115800	C	-3.33887100	-3.45667600	-2.25598700
H	-10.37744500	-4.35347800	-2.22973700	H	-2.90948800	-3.57965900	-3.24734100
H	12.80213500	0.06686400	-1.29940000	C	-2.54483000	-3.69758600	-1.12647600
C	-8.26478400	-4.46376200	-1.97801700	C	-3.17190400	-3.62943200	0.12367300
C	11.70213900	1.78143500	-0.67556400	H	-2.60703800	-3.85139600	1.02706600
H	-8.08571500	-4.35665600	-3.04491600	C	-4.45642200	-3.10390900	0.25107500
H	11.70376600	2.20388400	-1.67725000	H	-4.83159800	-2.93226300	1.25387200
C	-7.18933900	-4.41176700	-1.08052700	C	-1.06390200	-3.67819800	-1.24266500
C	10.91880400	2.36708000	0.32842500	C	-0.17841200	-4.47077600	-0.50208600
C	-7.46519200	-4.67629400	0.26835200	H	-0.56519500	-5.29007400	0.09990700
C	11.04958500	1.85355200	1.62652400	C	1.19312600	-4.21173600	-0.51168000
H	-6.65894900	-4.69101300	0.99781900	H	1.85266000	-4.84433100	0.07746600
H	10.49673300	2.30083600	2.44906300	C	1.72820600	-3.13963400	-1.24164400
C	-8.77521300	-4.73970900	0.72917600	C	0.84249300	-2.38056500	-2.01734000
C	11.73182100	0.66471100	1.85787800	H	1.20384500	-1.51092700	-2.55765300
H	-8.93824200	-4.83767800	1.79810000	C	-0.51705200	-2.64992900	-2.02497800
H	11.71365400	0.25011900	2.86118600	H	-1.18305100	-1.99705700	-2.58311000
C	-5.90826600	-3.76486500	-1.45757700	C	3.13190500	-2.67066900	-1.09918900
C	9.75882200	3.22843000	-0.00970800	C	3.73681200	-1.91053800	-2.11091300
C	-4.69021500	-3.99432900	-0.80248300	C	4.93431700	-1.24413900	-1.88886300
C	9.17141400	4.13686500	0.88200900	H	5.35733600	-0.61618600	-2.66924100
H	-4.58426600	-4.85571800	-0.14781400	C	5.59241900	-1.34027100	-0.65871800
H	9.70642000	4.43187700	1.78120000	C	5.05224900	-2.18407200	0.31554900
C	-3.62469100	-3.10661100	-0.93683200	H	5.55143800	-2.28020900	1.27759200
C	7.88776000	4.63087300	0.66086000	C	3.83271300	-2.82588600	0.10409800
H	-2.70485900	-3.31073800	-0.39559700	H	3.25265100	-1.81461900	-3.07831100
H	7.46094300	5.31643800	1.38787500	H	3.38979600	-3.39771600	0.91646100
C	-3.73634900	-1.94242000	-1.71347900	H	3.69418000	4.13652200	-1.85877800
C	7.12835400	4.22385800	-0.44756900	H	2.36547200	1.43750600	1.20909200
C	-4.91261500	-1.78261400	-2.45805100	H	7.79996400	2.73814400	-0.12168100
C	7.76660100	3.41295300	-1.39529700	H	10.01876800	1.64490900	0.01214400
H	-5.05937300	-0.89469500	-3.06399800	H	10.17913700	-0.83857500	-0.06250400
H	7.22340600	3.05822600	-2.26502000	H	8.12037500	-2.19329500	-0.35767300
C	-5.96485300	-2.67748600	-2.34240300	P	-2.84040400	0.17009000	1.98076000
C	9.05268100	2.93879400	-1.18698600	O	-3.77795100	-0.86633000	2.45594100
H	-6.88871000	-2.46441000	-2.87080100	O	-1.41009300	0.15038900	2.68930700

H	9.47287700	2.24279100	-1.90608200
C	-2.75953500	-0.82502300	-1.63867000
C	5.66645800	4.46803700	-0.55588200
C	-2.73829900	0.18327500	-2.61350900
C	4.99261800	4.31057500	-1.77711500
C	-2.02422300	1.35864700	-2.41694000
C	3.60567400	4.27310700	-1.83488200
H	-2.07473000	2.15207100	-3.15929100
H	3.10830200	4.08915800	-2.78388700
C	-1.26556700	1.55116600	-1.26108500
C	2.83726100	4.43612500	-0.67911000
C	-1.18753100	0.50422800	-0.33866400
C	3.49763700	4.70970100	0.52145900
H	-0.57831600	0.62337400	0.55473700
H	2.91588300	4.85244000	1.42956700
C	-1.93834200	-0.65372800	-0.51346000
C	4.88853100	4.70480200	0.58613000
H	-3.31051900	0.06858400	-3.52855500
H	-1.92482600	-1.40785500	0.26854800
H	-5.71672800	5.68564400	-1.58465200
H	-5.44998700	2.11415200	0.77500000
H	5.55573900	4.18629000	-2.69659200
H	5.36740800	4.82413300	1.55408800
H	2.13041000	-0.87112900	-3.21947400
H	4.08080600	0.06574200	0.48585700
H	-1.47306800	6.09386600	-0.27523600
H	0.98957700	6.33023500	-0.28338800
P	7.53544900	-0.92260000	2.16628600
O	8.49700100	-0.71930900	0.92627700
O	7.09520400	-2.28370100	2.52910500
C	8.19204500	-1.41797000	-0.31268000
H	8.19188700	-2.49204300	-0.10147100
H	7.19019900	-1.11824400	-0.64576400
C	9.26175000	-1.03095800	-1.30519600
H	10.23427000	-1.42782900	-0.99757500
H	9.01100700	-1.43306200	-2.29125200
H	9.33436000	0.05857900	-1.37076800
N	6.31011300	0.17506600	1.98544000
C	6.63081000	1.53465000	1.54172100
H	6.89623600	2.17991800	2.39262100
H	7.46506400	1.52086600	0.83574500
H	5.75464700	1.95655200	1.03586400
C	5.23534100	0.11804600	2.97819700
H	4.35041900	0.61699200	2.57023600
H	4.98980100	-0.92245300	3.19794300
H	5.52047300	0.63080500	3.90847900
C	8.63520100	-0.21235400	3.42738800
N	9.26562700	0.28603000	4.26029400
P	-7.75433200	-0.62315500	2.24352200
O	-8.33000500	-1.12869100	0.85871400
O	-8.56525400	0.28484800	3.07808500
C	-8.94818500	-0.16040000	-0.03333700
H	-9.79352500	0.29213000	0.49495400
H	-8.21265500	0.61995200	-0.26656000
C	-9.38426200	-0.91739000	-1.26454100
H	-10.18535300	-1.62166100	-1.01972700
H	-9.74984500	-0.21344700	-2.01810600
H	-8.54075200	-1.47815000	-1.67787600
N	-6.21715700	-0.09232500	1.93804200
C	-5.34996900	-0.89003100	1.06634600

F	-3.28496200	1.64449800	2.36749000
C	-2.57884800	0.30332300	0.21174700
H	-1.78687400	1.03004700	0.00377400
H	-2.29476000	-0.68248800	-0.17365200
H	-3.50613200	0.62085200	-0.27589900
C	1.02215800	-1.09038200	1.58205100
C	1.99415200	-0.85481200	2.73940500
C	1.31808000	-0.04727800	3.84935100
C	0.05724100	-0.76054200	4.34648300
C	-0.89721700	-1.10917100	3.21134500
C	-0.20494200	-1.85774900	2.07529000
H	2.00480500	0.11856000	4.68680200
H	2.31927800	-1.82765800	3.14017700
H	2.90056200	-0.34928900	2.38979500
H	0.69854700	-0.12494000	1.16530200
H	1.50736400	-1.63371500	0.76913700
H	0.33959500	-1.70241300	4.83714600
H	-0.47558400	-0.15469600	5.08713400
H	-1.75077300	-1.67901200	3.59250500
H	0.09760800	-2.84788300	2.44635200
H	-0.91554800	-2.03816100	1.25742000
H	1.04252600	0.94278400	3.46008200

7CPP_2R
E = -3463.162530

C	1.44286500	-3.86246600	-0.14494600
C	-0.70700200	-2.70300600	-0.27732500
C	-0.63068600	-5.11235300	-0.02697400
C	0.70188000	-2.66793500	-0.28407200
C	-1.37678600	-3.94028100	-0.15680100
C	0.75730700	-5.07406200	-0.02032800
C	2.93282100	-3.88055900	-0.06573500
C	-1.49570700	-1.43329700	-0.34267800
C	3.68909600	-4.75031100	-0.86064100
C	-1.38512100	-0.52791900	-1.40378400
H	3.18158300	-5.40063600	-1.56983100
H	-0.68845500	-0.73243100	-2.21286500
C	5.08078500	-4.78288400	-0.77331200
C	-2.14964800	0.63599000	-1.43616100
C	5.76384700	-3.94724900	0.11739400
C	-3.05748500	0.93425800	-0.41156700
C	4.99983400	-3.09154600	0.92599100
C	-3.15496100	0.02960800	0.65549400
C	3.61559300	-3.06176100	0.84094200
C	-2.38492000	-1.12550000	0.69165900
H	3.05702800	-2.38077400	1.47615300
H	-2.49269900	-1.81457700	1.52547500
C	7.24493800	-3.85631000	0.17965000
C	-3.97165700	2.10350400	-0.46405600
C	7.88405400	-3.54158200	1.38569300
C	-4.45329400	2.69095200	0.71285200
H	7.31344300	-3.54034900	2.31041400
H	-4.03236900	2.39914800	1.67113300
C	9.21556300	-3.14905700	1.41073700
C	-5.51476900	3.58648100	0.68696900
H	9.63918100	-2.79827500	2.34730300
H	-5.93041800	3.92941600	1.62995200
C	9.97422000	-3.07446700	0.23348300
C	-6.12730600	3.95605900	-0.51902800

H	-4.82291100	-1.67106800	1.63450400
H	-5.93439700	-1.36294700	0.27287100
H	-4.60835400	-0.22879700	0.60316100
C	-5.50956700	0.54773700	3.04893700
H	-4.71550400	1.18353700	2.64394200
H	-6.20324300	1.16603700	3.62127900
H	-5.05068000	-0.19820100	3.71374500
C	-7.55857800	-2.25677600	3.01557400
N	-7.35547200	-3.27301100	3.53085900

[7CPP_3R. GA]
E = -7473.589432

C	0.58951700	1.25838500	-1.66686900
C	-1.40733000	-0.15091500	-1.66773100
C	0.81164400	-1.17602100	-1.65581900
C	-0.81391000	1.12508400	-1.67210800
C	-0.59025400	-1.29985900	-1.66113900
C	1.39787000	0.10631600	-1.66366700
C	1.23554600	2.60719600	-1.58692600
C	-2.89897400	-0.26382900	-1.59479300
C	1.65811900	-2.40843700	-1.56824300
C	2.18440400	3.02790000	-2.52565600
C	-3.73037800	0.34437800	-2.54210800
C	1.55100600	-3.44529200	-2.50202100
H	2.42918100	2.38091400	-3.36388000
H	-3.28584400	0.87482200	-3.38004500
H	0.86473500	-3.34165400	-3.33831500
C	2.85044400	4.24362900	-2.38503300
C	-5.11721500	0.31695900	-2.41075800
C	2.28003900	-4.62428900	-2.36024000
C	2.57520600	5.09430700	-1.30767600
C	-5.72476300	-0.33976500	-1.33407100
C	3.16115700	-4.80088100	-1.28670600
C	1.58991000	4.69056300	-0.39475300
C	-4.88983300	-0.98859100	-0.41244600
C	3.29903800	-3.74157500	-0.37780500
C	0.94600000	3.46688000	-0.52097700
C	-3.50721900	-0.93784900	-0.52946000
C	2.55170300	-2.57839500	-0.50427600
H	0.20258400	3.17475500	0.21653100
H	-2.88886100	-1.43341900	0.21476000
H	2.66838800	-1.78532400	0.22998900
C	3.25051500	6.40101300	-1.11108800
C	-7.19535100	-0.40242700	-1.14541600
C	3.96710100	-6.03141900	-1.08975700
C	3.40940100	6.91919400	0.18110800
C	-7.73048500	-0.51304300	0.14482400
C	4.35204000	-6.41650000	0.20143500
H	3.14201900	6.31704500	1.04514700
H	-7.07917100	-0.44039700	1.01155600
H	3.97020700	-5.88016500	1.06585100
C	3.83464300	8.22377400	0.38711000
C	-9.07507100	-0.79018600	0.34599700
C	5.27771700	-7.42961500	0.40559400
H	3.80702500	8.61756600	1.39876900
H	-9.40783500	-1.00313300	1.35751200
H	5.64390100	-7.59258600	1.41492500
C	4.14580900	9.06571200	-0.69222800
C	-9.95509800	-0.94523900	-0.73674300
C	9.38039500	-3.55132700	-0.94446200
C	-5.53753700	3.49296800	-1.70455900
H	9.95007900	-3.56128600	-1.87003800
H	-5.94374600	3.79811200	-2.66526400
C	8.03957800	-3.92472800	-0.97305600
C	-4.49100700	2.57681600	-1.67738500
H	7.58455200	-4.19203000	-1.92375200
H	-4.12564400	2.16644200	-2.61518000
C	11.20090000	-2.23359700	0.22775900
C	-7.49398500	4.54207900	-0.48667300
C	11.43778300	-1.39824900	-0.87090200
C	-8.42710000	4.13113000	-1.44693200
H	10.89983600	-1.56036700	-1.80023900
H	-8.09407900	3.60768900	-2.33806500
C	12.18160400	-0.23261300	-0.73438200
C	-9.79047500	4.18373100	-1.18674400
H	12.18862400	0.47777500	-1.55613300
H	-10.47014700	3.69089100	-1.87605500
C	12.71052600	0.14435700	0.50805000
C	-10.27284600	4.64058800	0.04831400
C	12.69276000	-0.81849500	1.52936900
C	-9.35627400	5.28482800	0.89391600
H	13.17034900	-0.60355400	2.48255600
H	-9.70178500	5.73024200	1.82406600
C	11.95237600	-1.98808100	1.38954500
C	-7.99122000	5.23829900	0.62805100
H	11.88167000	-2.67107300	2.23247100
H	-7.29950900	5.66749800	1.34839100
C	12.78646200	1.60675300	0.77472700
C	-11.51823100	3.99142400	0.54234000
C	12.09423100	2.06498300	1.90340400
C	-11.42306700	3.34239800	1.78053300
H	11.89415600	1.38209600	2.72418200
H	-10.66985600	3.66007500	2.49618900
C	11.41720700	3.27782000	1.87565600
C	-12.06780900	2.13018100	1.99528000
H	10.72070400	3.48543900	2.68138800
H	-11.78820200	1.55755600	2.87363200
C	11.41816700	4.07521700	0.72209600
C	-12.83333500	1.53538800	0.98227500
C	12.39391600	3.77383300	-0.24608700
C	-13.20612500	2.36623200	-0.09050400
H	12.53356400	4.43029300	-1.10102500
H	-13.90591300	2.00591200	-0.84008800
C	13.05920000	2.55261400	-0.22761000
C	-12.54956300	3.57168600	-0.31454000
H	13.68957300	2.28064400	-1.07114000
H	-12.74323800	4.11618900	-1.23594700
C	10.15488500	4.78825600	0.37105100
C	-12.75213400	0.05220600	0.83747800
C	9.75083300	4.77696000	-0.97392900
C	-12.61212300	-0.46591200	-0.46052900
H	10.48639300	4.64195100	-1.76054700
H	-12.96376700	0.10528100	-1.31402200
C	8.40686400	4.71893900	-1.31619500
C	-11.83948600	-1.59351200	-0.70034300
H	8.13017600	4.57871900	-2.35819800
H	-11.64203000	-1.89319300	-1.72658900
C	7.41959000	4.65299700	-0.32430300
C	-11.16517900	-2.23350500	0.34791300

C	5.84506900	-8.12428500	-0.67436700
C	4.11540400	8.50527500	-1.97835400
C	-9.44686300	-0.70299600	-2.02212500
C	5.36218400	-7.82837600	-1.95817700
H	4.38389400	9.11123000	-2.83992900
H	-10.10193500	-0.77689500	-2.88647100
H	5.74888800	-8.36674300	-2.81974700
C	3.66685900	7.20287000	-2.18387900
C	-8.09249600	-0.44710000	-2.22259000
C	4.44957500	-6.79611900	-2.16204500
H	3.57726900	6.83071300	-3.20129500
H	-7.71966700	-0.34706800	-3.23881000
H	4.16113700	-6.54099200	-3.17861700
C	4.14371800	10.53180100	-0.44416700
C	-11.22850000	-1.67217500	-0.48905200
C	7.12068800	-8.84859600	-0.43128600
C	3.51417800	11.37686600	-1.36747800
C	-11.64610500	-2.64530800	-1.40638400
C	8.16179700	-8.72476900	-1.36064400
H	3.35274800	11.04011200	-2.38798600
H	-11.26946800	-2.62637400	-2.42556500
H	7.94398400	-8.42118700	-2.38114900
C	2.86263800	12.53122700	-0.94283900
C	-12.32656700	-3.78049700	-0.97527000
C	9.48959900	-8.73027200	-0.94280700
H	2.20876200	13.04449000	-1.64306900
H	-12.44523500	-4.60842200	-1.66937600
H	10.25606700	-8.41992000	-1.64821500
C	2.82153800	12.86915400	0.41723000
C	-12.60431100	-3.97328100	0.38537700
C	9.81046500	-8.85689600	0.41616500
C	3.72490000	12.20569900	1.26559900
C	-12.48021100	-2.85302400	1.22539500
C	8.79063600	-9.30889700	1.27162000
H	3.80891700	12.50520100	2.30704100
H	-12.78562400	-2.92064900	2.26629500
H	9.01437400	-9.52618000	2.31282600
C	4.37520100	11.05455600	0.84072800
C	-11.80233300	-1.72036900	0.79399200
C	7.46634100	-9.30414300	0.85349600
H	4.96039400	10.48724300	1.55908400
H	-11.60284900	-0.92506700	1.50670800
H	6.68692600	-9.52785400	1.57657500
C	1.55691800	13.45988000	0.93299400
C	-12.49070500	-5.36019800	0.91263400
C	10.95367700	-8.05023700	0.92311600
C	0.96917800	12.78663300	2.01443900
C	-11.61918800	-5.52675600	1.99941300
C	10.66619700	-7.20100300	2.00219800
H	1.57528600	12.14591200	2.64944800
H	-11.36713300	-4.67717500	2.62855300
H	9.81150700	-7.40566300	2.64155000
C	-0.41084800	12.70539900	2.13651000
C	-10.86378200	-6.68299900	2.13463300
C	11.28234700	-5.96270600	2.11634200
H	-0.81177700	12.01382400	2.87041100
H	-10.06705700	-6.68059000	2.87150700
H	10.88401800	-5.26751500	2.84825900
C	-1.25592300	13.29143600	1.18323100
C	-10.94849600	-7.71580700	1.18991400
C	7.80941700	4.95940800	0.98677800
C	-11.54295500	-1.89264200	1.65396300
H	7.06335200	4.98141300	1.77794000
H	-11.08854800	-2.40871000	2.49661500
C	9.15740300	5.04357000	1.32631400
C	-12.34048700	-0.77611500	1.89441400
H	9.42264500	5.15868000	2.37363200
H	-12.50953500	-0.47010600	2.92326500
C	6.15191200	3.92345600	-0.58814400
C	-9.87613600	-2.92387500	0.08169700
C	4.91600200	4.19127900	0.01360600
C	-9.37394100	-4.02183000	0.79078400
H	4.77534500	5.11637100	0.56806000
H	-10.02358100	-4.56771400	1.47118200
C	3.86676200	3.27487700	-0.06952900
C	-8.04215300	-4.41448900	0.65046400
H	2.92002900	3.51006600	0.41060600
H	-7.68272600	-5.27117600	1.21535000
C	4.01884300	2.05219800	-0.74028100
C	-7.15970000	-3.71532700	-0.18632500
C	5.23662800	1.81818300	-1.39051000
C	-7.68308000	-2.65426100	-0.93502800
H	5.41245700	0.86685600	-1.88314700
H	-7.02739900	-2.05776200	-1.56169500
C	6.27312300	2.73402800	-1.32248800
C	-9.00888300	-2.27413100	-0.80948000
H	7.23230800	2.47574600	-1.76123800
H	-9.35440200	-1.39161500	-1.33968500
C	3.01585400	0.95689000	-0.69368200
C	-5.69484600	-3.96197400	-0.22620000
C	2.95540400	0.00764000	-1.72417700
C	-4.94909600	-3.62834400	-1.36762000
C	2.19062400	-1.14436800	-1.59509500
C	-3.56172800	-3.63984800	-1.34846200
H	2.19878800	-1.89141900	-2.38604400
H	-3.00452200	-3.33086600	-2.22950700
C	1.42575600	-1.37049500	-0.44855700
C	-2.86508800	-4.02290500	-0.19794000
C	1.40509000	-0.38323200	0.54005600
C	-3.60142300	-4.45042700	0.91015900
H	0.79579400	-0.53090000	1.42935100
H	-3.07648000	-4.75861000	1.81193600
C	2.20024200	0.75437400	0.42757600
C	-4.99480700	-4.40522300	0.90234100
H	3.53077800	0.15992300	-2.63271200
H	2.22657900	1.46606300	1.24861000
H	5.63967600	-5.47077200	-1.40338300
H	5.50004300	-2.40714800	1.60529600
H	-5.46141200	-3.33718900	-2.27988500
H	-5.53893000	-4.66030900	1.80792000
H	-2.02858600	1.32576600	-2.26756600
H	-3.87621300	0.20537100	1.44820000
H	1.32486300	-5.99234100	0.10777600
H	-1.15127900	-6.06274800	0.06053300

7CPP_3R
E = -5078.670489

C	-0.91107600	-1.06956700	-0.69691000

C	12.20742500	-5.52477700	1.15765800	C	1.38920700	-0.25203800	-0.69671000
C	-0.66488400	14.21292500	0.29941700	C	-0.46907500	1.33135800	-0.69562500
C	-12.03767000	-7.66789900	0.30042100	C	0.47631800	-1.32191500	-0.70163700
C	12.70895700	-6.49925600	0.27538700	C	0.91389000	1.07572200	-0.70026600
H	-1.28504000	14.78381900	-0.38675300	C	-1.38099400	0.25578800	-0.69970700
H	-12.22189700	-8.49534400	-0.37987000	C	-1.88398000	-2.20405200	-0.62281600
H	13.50956700	-6.24788600	-0.41544300	C	2.85824200	-0.52743500	-0.62224600
C	0.71961000	14.29134000	0.17036100	C	-0.96596900	2.74095600	-0.62202600
C	-12.79280500	-6.50624900	0.15809800	C	-2.86374700	-2.41333100	-1.59939600
C	12.08784200	-7.73999100	0.15407100	C	3.52908900	-1.26892100	-1.60074100
H	1.14266500	14.91404700	-0.61446300	C	-0.66092000	3.69216500	-1.60166300
H	-13.53966800	-6.45489700	-0.63080400	H	-2.93201700	-1.73159600	-2.44342800
H	12.41428500	-8.41985800	-0.62945400	H	2.97267800	-1.66589500	-2.44608100
C	-2.52817800	12.58126600	0.87048900	H	-0.03839000	3.40901200	-2.44670800
C	-9.69794500	-8.46901000	0.89034400	C	-3.76133000	-3.47389600	-1.50067500
C	12.22172300	-4.06877900	0.84019100	C	4.89621700	-1.51692700	-1.50267200
C	-2.93940900	12.49343400	-0.46954400	C	-1.13302500	4.99911900	-1.50573400
C	-9.40789200	-8.78950900	-0.44597700	C	-3.70631800	-4.36907000	-0.42408900
C	12.33926300	-3.67230000	-0.50208700	C	5.64427800	-1.02498600	-0.42478900
H	-2.58748800	13.22151200	-1.19461600	C	-1.93426300	5.40073300	-0.42862600
H	-10.20964900	-8.85117200	-1.17615900	C	-2.72733000	-4.14897700	0.55584900
H	12.79192100	-4.34109300	-1.22843100	C	4.96441700	-0.28944100	0.55704000
C	-3.63086700	11.38234000	-0.93528800	C	-2.22883100	4.44503300	0.55478900
C	-8.09681200	-8.83742500	-0.90275700	C	-1.84074600	-3.08434100	0.46230800
C	11.71283900	-2.52285600	-0.96684700	C	3.59914400	-0.05299200	0.46420800
H	-3.83974700	11.29762400	-1.99878700	C	-1.74831200	3.14532500	0.46368700
H	-7.91215800	-8.98211500	-1.96430700	H	-1.08653000	-2.94693700	1.23266200
H	11.73475700	-2.30291100	-2.03131200	H	3.10331400	0.53021700	1.23573100
C	-3.91353000	10.30840900	-0.08016700	H	-2.00386100	2.42515600	1.23649600
C	-7.03061800	-8.54184200	-0.04215300	C	-4.56754100	-5.57542900	-0.33414000
C	10.92567500	-1.74309100	-0.10831200	C	7.11975200	-1.16858800	-0.33529000
C	-3.73601300	10.51835300	1.29480000	C	-2.55127200	6.74882300	-0.34170300
C	-7.31026800	-8.48524900	1.33068000	C	-4.89119400	-6.13280300	0.90987700
C	11.03147300	-1.99622100	1.26673400	C	7.76518700	-1.16858800	0.90830100
H	-3.98253900	9.73142600	2.00361600	C	-2.87341200	7.31028200	0.90090900
H	-6.51000800	-8.30237900	2.04383700	H	-4.65610200	-5.58935300	1.82067600
H	10.47511400	-1.39039200	1.97792400	H	7.17765700	-1.23462000	1.81965500
C	-3.07677000	11.64840800	1.76462600	H	-2.51930000	6.83835900	1.81307400
C	-8.62169700	-8.47450500	1.79165400	C	-5.43771500	-7.40595500	1.00695900
C	11.69061500	-3.12681800	1.73529800	C	9.14116700	-1.00588700	1.00440300
H	-2.86655200	11.71416200	2.82775800	C	-3.70490500	8.41878000	0.99535500
H	-8.79074500	-8.31887800	2.85281300	H	-5.56328700	-7.84483300	1.99222900
H	11.65203600	-3.33829300	2.79954900	H	9.58466700	-0.89345700	1.98922200
C	-4.04174300	8.92567400	-0.60290400	H	-4.02265200	8.74885900	1.97986500
C	-5.76434700	-7.96661900	-0.55951900	C	-5.71213700	-8.17072000	-0.13624300
C	9.78228600	-0.95055300	-0.62460200	C	9.94017000	-0.86364400	-0.13937300
C	-4.68399900	7.88526100	0.08299900	C	-4.23147400	9.03489200	-0.14919200
C	-4.54713500	-8.00035200	0.13537800	C	-5.54423000	-7.54363800	-1.38006000
C	9.19724500	0.12099100	0.06462200	C	9.31232200	-1.03379900	-1.38251700
H	-5.34226300	8.11513300	0.91709900	C	-3.77175700	8.57370200	-1.39190400
H	-4.42480300	-8.67957100	0.97532000	H	-5.79715400	-8.07590300	-2.29292000
H	9.72646000	0.58053600	0.89565900	H	9.89932400	-0.98908700	-2.29580400
C	-4.45246700	6.55367100	-0.25444300	H	-4.10769900	9.05578300	-2.30592900
C	-3.50458200	-7.14002800	-0.20166400	C	-4.97194100	-6.27919300	-1.47729400
C	7.92269400	0.57618700	-0.26572000	C	7.93097300	-1.16963000	-1.47880800
H	-4.95446900	5.77656900	0.31546600	C	-2.96016700	7.44750100	-1.48642700
H	-2.58452100	-7.18493700	0.37469400	H	-4.76528800	-5.86980600	-2.46263100
H	7.49751500	1.39616600	0.30657300	H	7.47279400	-1.19704500	-2.46395400
C	-3.55460500	6.20362600	-1.27552600	H	-2.70811200	7.06246500	-2.47100700
C	-3.63997400	-6.19565400	-1.23170200	C	-5.86456800	-9.64406100	-0.00247400

C	7.16978800	-0.03213100	-1.28280200	C	11.29219400	-0.25836700	-0.00756900
C	-3.01318700	7.24868500	-2.03554600	C	-5.43285500	9.90140400	-0.01683500
C	-4.81151200	-6.25053000	-1.99827100	C	-5.29847200	-10.47355800	-0.97891000
C	7.80767400	-1.01922700	-2.04558600	C	11.72443900	0.64808900	-0.98397200
H	-2.30477500	7.03676800	-2.82970700	C	-6.43516200	9.82088100	-0.99199200
H	-4.97448700	-5.53626400	-2.79874100	H	-4.99097500	-10.05677100	-1.93295100
H	7.26960600	-1.53154300	-2.83641300	H	11.20757500	0.70632300	-1.93682200
C	-3.26149900	8.57443800	-1.71496000	H	-6.22767000	9.34449700	-1.94516900
C	-5.84048600	-7.12279000	-1.67796100	C	-4.92130000	-11.77618800	-0.67899100
C	9.08538100	-1.45669200	-1.73223500	C	12.66317400	1.62720000	-0.68506000
H	-2.75055100	9.34974600	-2.27707100	C	-7.75240800	10.14327600	-0.69164700
H	-6.76344000	-7.06919800	-2.24669700	H	-4.32412400	-12.32280000	-1.40297200
H	9.50493100	-2.28322300	-2.29693400	H	12.83528800	2.41865300	-1.40860800
C	-3.04177300	4.82091600	-1.45626600	H	-8.52440200	9.89632900	-1.41456500
C	-2.69183900	-5.06702100	-1.41826200	C	-5.08668800	-12.29739300	0.61250300
C	5.71351200	0.20553800	-1.45783100	C	13.19960400	1.74403100	0.60551400
C	-2.38411500	4.43808500	-2.63464600	C	-8.12067900	10.54875500	0.59936600
C	-2.67670200	-4.31983100	-2.60530200	C	-5.88190100	-11.55801600	1.50252000
C	5.05091300	-0.17782200	-2.63337400	C	12.96022200	0.68443000	1.49490200
C	-1.68823300	3.23798500	-2.71322300	C	-7.08258800	10.87300100	1.48733100
C	-1.97940200	-3.12115200	-2.69245500	H	-6.11064600	-11.96226600	2.48579700
C	3.66340200	-0.18815600	-2.70692800	H	13.42667900	0.68824800	2.47723900
H	-1.13965000	2.98983800	-3.61917900	H	-7.31830400	11.27545300	2.46970200
H	-2.03081200	-2.53145300	-3.60505300	C	-6.26665400	-10.25610000	1.19726700
H	3.17295100	-0.54268000	-3.61086400	C	12.02553400	-0.30046500	1.19088200
C	-1.66187800	2.35425000	-1.63314100	C	-5.76219300	10.55807000	1.18151700
C	-1.23265600	-2.64763500	-1.61248200	H	-6.80617000	-9.67914000	1.94384500
C	2.88631100	0.22851600	-1.62498100	H	11.79759300	-1.05666700	1.93760400
C	-2.39799600	2.68135500	-0.49160200	H	-4.99245100	10.74081500	1.92683400
C	-1.15848700	-3.43682900	-0.46197300	C	-4.05560000	-13.27333800	1.05889800
C	3.53911200	0.70719100	-0.48616800	C	13.52815400	3.12460800	1.05376500
H	-2.41106900	1.99311200	0.35093100	C	-9.47960300	10.14151700	1.04917000
H	-0.56000900	-3.09757200	0.38088800	C	-3.34197900	-12.93374100	2.21594400
H	2.95077900	1.06180300	0.35756800	C	12.87930900	3.56883300	2.21361000
C	-3.05895100	3.90112400	-0.39692200	C	-9.53853000	9.35724400	2.20885300
C	-1.89118500	-4.61430400	-0.35875400	H	-3.79049700	-12.26811000	2.94790100
C	4.92650300	0.67739500	-0.39633900	H	12.52949900	2.84513000	2.94425500
H	-2.38966800	5.09429100	-3.49916400	H	-8.73640500	9.41625200	2.93894300
H	-3.54964400	4.15850200	0.53762300	C	-1.98000200	-13.19326100	2.30841400
H	3.61404900	4.51590300	-3.10897900	C	12.42243500	4.87744100	2.31111600
H	1.31217600	5.36294900	0.41234200	C	-10.44306200	8.30705200	2.30708600
H	5.62041400	-0.49882800	-3.49974600	H	-1.42941800	-12.71799800	3.11353100
H	5.39638400	0.97801000	0.53599900	H	11.73747800	5.11375900	3.11868600
H	2.13669100	-5.42511000	-3.08096700	H	-10.30440200	7.59561000	3.11443300
H	4.02435500	-3.82841400	0.42642600	C	-1.29162700	-13.80540200	1.25106200
H	-3.23758200	-4.66009500	-3.46998400	C	12.60616300	5.78343900	1.25656500
H	-1.87784400	-5.15743100	0.58210700	C	-11.31962500	8.01242200	1.25286800
H	-5.72880600	0.83976300	-3.14152800	C	-2.08105200	-14.45298500	0.28227900
H	-5.34007200	-1.55979500	0.39484500	C	13.55965300	5.42699200	0.28456800
P	-7.71829700	-4.09466300	2.29004000	C	-11.48895200	9.01625400	0.28100100
O	-8.25505800	-4.92963600	1.05824900	H	-1.61046000	-15.05628500	-0.48953900
O	-8.56178900	-3.03286400	2.87260800	H	13.84521700	6.13889500	-0.48535400
C	-8.88184600	-4.22214600	-0.04721900	H	-12.24846800	8.90717000	-0.48863300
H	-9.74281200	-3.67508000	0.34948400	C	-3.44154300	-14.18296200	0.18112800
H	-8.15949600	-3.50527400	-0.45795100	C	14.00613400	4.11417100	0.17791000
C	-9.28544400	-5.26726800	-1.05909500	C	-10.57619200	10.06012500	0.17417900
H	-10.07833900	-5.90605400	-0.65775300	H	-3.99594100	-14.57132800	-0.67005700
H	-9.64975000	-4.77859800	-1.96765600	H	14.61848900	3.83137000	-0.67517500
H	-8.42570800	-5.89517700	-1.31009300	H	-10.63846000	10.73172400	-0.67892000
N	-6.18400100	-3.61827500	1.89143300	C	0.08288600	-13.31063900	0.94668300

C	-5.29306200	-4.57294700	1.22554600
H	-4.76480700	-5.20325400	1.95671800
H	-5.85901800	-5.21717700	0.54788100
H	-4.55461300	-4.01520600	0.63804600
C	-5.50289900	-2.75249000	2.85596500
H	-4.68109000	-2.23675600	2.34883600
H	-6.20281600	-2.01192700	3.24710300
H	-5.08139600	-3.33324100	3.68950500
C	-7.50374500	-5.49068000	3.43452600
N	-7.28591900	-6.34762600	4.18139800
P	0.24862500	8.67692900	2.30369900
O	-0.18841700	9.56470000	1.06924500
O	1.58085900	8.87461700	2.90739900
C	0.75597500	9.76071200	-0.01969200
H	1.64797600	10.24281600	0.39255700
H	1.03299100	8.77858800	-0.42360600
C	0.06341300	10.62366100	-1.04682900
H	-0.10897600	11.62964300	-0.65203500
H	0.68511600	10.69711100	-1.94409900
H	-0.90182200	10.18416900	-1.31469900
N	-0.09788400	7.11162600	1.89206900
C	-1.35953500	6.81877700	1.20570700
H	-2.17955600	6.66962600	1.92422000
H	-1.62694800	7.63473500	0.52936700
H	-1.23467400	5.90459600	0.61398600
C	0.29875000	6.08393700	2.85665800
H	0.34306000	5.11730300	2.34458800
H	1.28398700	6.31869200	3.26368300
H	-0.42695700	6.00345600	3.67923300
C	-1.08487600	9.18343100	3.43048500
N	-1.94759900	9.41969600	4.16502100
P	7.46169200	-4.55490900	2.29960700
O	8.43792500	-4.60671900	1.05573200
O	6.98978000	-5.81462700	2.90706000
C	8.13682100	-5.52454800	-0.03166600
H	8.10901300	-6.53735800	0.38213900
H	7.14768000	-5.27484400	-0.43617000
C	9.23099800	-5.35689300	-1.05816700
H	10.18910800	-5.70432700	-0.65935200
H	8.98799600	-5.93773700	-1.95293500
H	9.32871500	-4.30230600	-1.33139700
N	6.26167900	-3.48715400	1.90053400
C	6.61822400	-2.24195400	1.21419300
H	6.89830000	-1.45634100	1.93213200
H	7.45341400	-2.40755600	0.52877000
H	5.75434900	-1.90014700	0.63246400
C	5.17993800	-3.33299300	2.87516600
H	4.30966100	-2.89989200	2.37160200
H	4.90822200	-4.30797800	3.28364600
H	5.47134700	-2.66120300	3.69586300
C	8.56610400	-3.63997900	3.41665400
N	9.20095900	-3.00409600	4.14624000

[TET_7R1.GB]
E = -3058.428903

C	4.37457500	-3.89398600	-0.32708900
C	3.74600700	-4.80004100	0.53719400
H	4.34604000	-5.33655700	1.26776700
C	2.37550900	-5.02648500	0.47332200
C	11.49017200	6.72784600	0.95753900
C	-11.57765500	6.57323700	0.95435400
C	0.41474100	-13.09951200	-0.40182700
C	11.14037800	6.91591100	-0.38975400
C	-11.56289000	6.17508300	-0.39266700
H	-0.10410400	-13.64935000	-1.18072900
H	11.87541900	6.74500600	-1.16996600
H	-11.78270400	6.89587200	-1.17394100
C	1.23932200	-12.04857700	-0.77901600
C	9.81761700	7.10600200	-0.76506100
C	-11.06336600	4.93500100	-0.76598700
H	1.37307900	-11.83190900	-1.83579900
H	9.56227100	7.11817800	-1.82158800
H	-10.94411100	4.70703000	-1.82212000
C	1.74753200	-11.16043300	0.17820200
C	8.79520300	7.09751000	0.19306100
C	-10.54431700	4.05580900	0.19370700
C	1.65618000	-11.54878800	1.52218100
C	9.17831000	7.20670100	1.53720800
C	-10.83337800	4.33342100	1.53715300
H	2.09320100	-10.91998000	2.29429500
H	8.41586400	7.26714800	2.31021300
H	-10.50444200	3.64427600	2.31137400
C	0.84998600	-12.62005900	1.89947600
C	10.50940600	7.04265500	1.91266000
C	-11.35985100	5.56763500	1.91059500
H	0.70499200	-12.81303300	2.95875500
H	10.74993900	7.00921500	2.97157700
H	-11.45316000	5.79367200	2.96915400
C	2.00453100	-9.74353100	-0.18762400
C	7.43949700	6.61274700	-0.17353600
C	-9.44378000	3.12683500	-0.17104900
C	2.94856700	-8.90556700	0.41932100
C	6.24253000	7.00740100	0.43749500
C	-9.18556200	1.89268100	0.43874800
H	3.70806000	-9.33175100	1.07062800
H	6.23251900	7.87474200	1.09344600
H	-9.93187400	1.44779700	1.09275700
C	2.91353500	-7.52543000	0.21767700
C	5.06501200	6.28709100	0.23446700
C	-7.97150200	1.23555800	0.23620700
H	3.65840600	-6.90091800	0.70442600
H	4.15247100	6.61670000	0.72478000
H	-7.79977900	0.27983400	0.72494600
C	1.92579100	-6.93053300	-0.58178000
C	5.04304100	5.13813000	-0.57059600
C	-6.96556200	1.79415300	-0.56675500
C	1.02524500	-7.77934400	-1.23670900
C	6.22750800	4.78689900	-1.22945900
C	-7.25462800	2.99637500	-1.22359400
H	0.22117900	-7.35808000	-1.83232600
H	6.26471600	3.88286000	-1.82948800
H	-6.49003700	3.48299700	-1.82139200
C	1.06825400	-9.15123300	-1.04904200
C	7.39389300	5.51005600	-1.04040300
C	-8.46564500	3.64210200	-1.03547100
H	0.29561500	-9.76737200	-1.49888800
H	8.31324000	5.15195700	-1.49371000
H	-8.61636800	4.61779600	-1.48740700
C	1.71869100	-5.46234100	-0.66768000

C	1.57674500	-4.34541800	-0.45251000
C	2.20462100	-3.42907100	-1.30853200
C	3.57659200	-3.21006100	-1.25221000
H	4.03764800	-2.51282300	-1.94729400
C	0.10614900	-4.53574300	-0.50481600
C	-0.59566200	-4.39957600	-1.70884100
H	-0.04906600	-4.26185400	-2.63794900
C	-1.98512600	-4.38733200	-1.73141600
H	-2.49651000	-4.18718400	-2.66898800
C	-2.73109600	-4.52785900	-0.55339000
C	-2.02851700	-4.78528900	0.63184100
H	-2.57524600	-4.94270900	1.55819300
C	-0.63873900	-4.77941900	0.65740600
H	-0.12737200	-4.90384200	1.60835600
C	-4.16556700	-4.14691800	-0.55045200
C	-4.65078600	-3.38700300	0.51970800
H	-4.04179400	-3.25516700	1.40920300
C	-5.79624900	-2.61424000	0.38638700
H	-6.04611600	-1.91471700	1.18101400
C	-6.49282500	-2.55944000	-0.82976900
C	-6.13257100	-3.48798900	-1.81926800
H	-6.69725700	-3.53432000	-2.74733800
C	-4.98852100	-4.26732500	-1.68180300
H	-4.68873900	-4.91797200	-2.49981000
C	-7.20614300	-1.28973800	-1.13358000
C	-7.72786100	-0.46916200	-0.12385600
H	-8.02981400	-0.89912700	0.82777100
C	-7.68420800	0.91595400	-0.24037100
H	-7.95422400	1.50370800	0.62999700
C	-7.10905700	1.52777900	-1.36610200
C	-6.89390600	0.70311400	-2.48278800
H	-6.51216900	1.11735000	-3.40944000
C	-6.93818300	-0.67982200	-2.36899500
H	-6.57509800	-1.27835200	-3.19980400
C	-6.35069200	2.80791300	-1.24778000
C	-5.59680400	3.30208900	-2.32479900
H	-5.84168600	3.01954900	-3.34359500
C	-4.42972600	4.02560500	-2.11297600
H	-3.80205700	4.25151200	-2.97004200
C	-3.98368600	4.32477200	-0.81679300
C	-4.87935300	4.06534600	0.23097900
H	-4.62915900	4.35481800	1.24783700
C	-6.03133700	3.31474400	0.02278700
H	-6.59324000	3.00490700	0.89708500
C	-2.55155300	4.63312100	-0.56493200
C	-1.96230800	4.20386700	0.63337700
C	-0.58282000	4.19397700	0.79610700
H	-0.15851500	3.78478400	1.70959500
C	0.26635000	4.59702800	-0.23916200
C	-0.31868000	5.10866300	-1.40492300
H	0.31934700	5.47849000	-2.20412400
C	-1.70095000	5.13694000	-1.56032600
H	-2.57809300	3.78198300	1.41977000
H	-2.11759500	5.53123300	-2.48314800
H	1.92554400	-5.75534500	1.14243900
H	1.60776500	-2.86305800	-2.01838500
C	8.16238900	4.77993600	0.46814000
C	8.80689300	3.59364500	0.31503400
C	8.06627800	2.37571400	0.14397300
C	6.61836700	2.41392300	0.12403400
C	3.87573000	4.22405600	-0.65854200
C	-5.58941400	1.24254600	-0.65513000
C	1.11728200	-4.89095300	-1.79834300
C	3.68167500	3.42088600	-1.79172700
C	-4.79774100	1.47761600	-1.78856400
C	0.73393600	-3.55563800	-1.81048700
C	2.71799700	2.42029900	-1.80651200
C	-3.44893500	1.14492400	-1.80417200
H	0.23114900	-3.14495100	-2.68331200
H	2.61402400	1.78191900	-2.68111700
H	-2.84502700	1.37413500	-2.67938700
C	0.96943000	-2.73335300	-0.70613300
C	1.88917600	2.20876800	-0.70207000
C	-2.85008300	0.53325400	-0.70035200
C	1.64042400	-3.27596800	0.39313300
C	2.02303100	3.05811500	0.39955500
C	-3.65129200	0.22402700	0.40200600
H	1.85113100	-2.64561900	1.25432800
H	1.37189300	2.92251700	1.26042300
H	-3.20680400	-0.27093600	1.26274700
C	1.99622600	-4.62149200	0.41843700
C	3.00949800	4.03978700	0.42741500
C	-4.99515200	0.58563400	0.43071300
H	0.93097600	-5.50184000	-2.67685800
H	2.45048100	-5.03302500	1.31573200
H	-4.52136900	-3.59695200	-2.26787300
H	-2.62684600	-4.84503800	1.38326900
H	-5.23742400	1.94155000	-2.66669300
H	-5.57547600	0.39862900	1.33022500
H	-0.86259100	5.71689900	-2.27580500
H	-2.88052200	4.70680700	1.38298600
H	4.30307000	3.56828800	-2.67022200
H	3.13868700	4.63611700	1.32661600
H	5.38239300	-2.11174900	-2.27154500
H	5.51696600	0.14334000	1.38557900

TET_7R1
E = -2308.414027

C	-3.62898500	3.91378200	-0.04332000
C	-3.08672800	4.79773200	0.89949100
H	-3.75180000	5.29254000	1.60272900
C	-1.72104700	5.05516900	0.94586000
C	-0.84107900	4.42913000	0.05562600
C	-1.38265200	3.53100800	-0.87625800
C	-2.74947900	3.28041100	-0.92923400
H	-3.14259600	2.59926100	-1.67988100
C	0.62504900	4.65066600	0.11650700
C	1.41342700	4.57320100	-1.03824400
H	0.93695000	4.46843900	-2.00922300
C	2.80044500	4.56980800	-0.95986400
H	3.37745600	4.40672000	-1.86579900
C	3.45892400	4.66256200	0.27421400
C	2.67022300	4.87074800	1.41406400
H	3.14522700	4.98968800	2.38440300
C	1.28193500	4.85531200	1.33779500
H	0.70262900	4.93446200	2.25402300
C	4.88844700	4.26866300	0.35975700
C	5.30226700	3.47270600	1.43477400
H	4.65179600	3.34859100	2.29571200

C	5.97213400	3.70933200	0.23773900
C	6.73392100	4.83084300	0.42183200
C	8.72114400	1.16063900	0.02201100
C	5.91614000	1.21503700	0.03483900
C	6.57690400	-0.03184500	-0.06376900
C	8.01661500	-0.05153200	-0.09029800
C	8.67962400	-1.28688400	-0.19471300
H	9.76808900	-1.30133100	-0.20291500
C	7.98662000	-2.48452600	-0.27078300
C	6.53692500	-2.48092100	-0.24460400
C	5.87727500	-1.25979700	-0.12917600
H	9.80948800	1.13853600	0.03021900
H	8.72141000	5.70057500	0.60679600
H	9.89262400	3.53837600	0.32887200
H	6.23788200	5.79489600	0.50156500
H	4.82873300	1.22762700	0.05464800
C	6.59554100	-4.91593200	-0.37256400
C	8.02413700	-4.90821100	-0.38926200
C	8.69715800	-3.72897000	-0.34861200
C	5.85585700	-3.76473000	-0.31123600
H	6.07689500	-5.86851500	-0.44326600
H	8.55953100	-5.85114500	-0.44846600
H	9.78378100	-3.70059100	-0.36979600
H	4.79222700	-1.23451200	-0.07167400
C	1.73079000	4.39386100	-0.11888000
C	2.41571500	4.72116600	1.05631300
C	2.45028800	3.79672100	-1.16294800
C	3.77932300	4.47064200	1.17860400
H	1.87794800	5.19362900	1.87475600
C	3.80954200	3.53053700	-1.03436900
H	1.92945600	3.51607600	-2.07517600
C	4.49659600	3.87200800	0.13688800
H	4.30178400	4.74083900	2.09286900
H	4.35187700	3.06782700	-1.85574300
P	-4.33750000	0.55970500	1.64870200
O	-5.68923900	0.90607000	2.13098000
O	-3.70513700	-0.77785500	2.24614800
C	-4.01019100	0.43749600	-0.10478500
H	-4.75760000	-0.21353300	-0.56824100
H	-4.07252400	1.43558700	-0.55005500
H	-3.01208400	0.01947700	-0.25646600
F	-3.27247500	1.65138000	2.13111500
C	-3.79891400	-1.04403300	3.67438900
H	-3.77253800	-0.08192400	4.20147000
C	-2.56958700	-1.85991500	4.02642300
H	-2.56842800	-2.09768000	5.09454500
H	-2.56901200	-2.79806800	3.46058400
H	-1.65642900	-1.31029800	3.78421000
C	-5.10397400	-1.76199000	3.97311900
H	-5.95789500	-1.14986400	3.67081000
H	-5.14189200	-2.72027800	3.44297300
H	-5.17892900	-1.96197400	5.04700000

[TET_7R1.GF]
E = -3175.131877

C	-4.25193200	3.86782200	-0.18031000
C	-3.69953200	4.75925600	0.74960700
H	-4.35033700	5.22478800	1.48539200
C	-2.34145100	5.05762900	0.74462000
C	6.43454500	2.67425400	1.33714300
H	6.62740300	1.94460300	2.11836900
C	7.19229500	2.64024000	0.15847500
C	6.90895300	3.60625700	-0.81912100
H	7.52561100	3.66479500	-1.71297400
C	5.77626100	4.40691100	-0.71939700
H	5.53563000	5.08847300	-1.53161600
C	7.89684100	1.36814900	-0.14913200
C	8.44455800	0.53976400	0.84268000
H	8.77614600	0.97027000	1.78476300
C	8.43102800	-0.84334500	0.69787400
H	8.76426000	-1.45539100	1.53130900
C	7.85174300	-1.44476400	-0.43355900
C	7.59272800	-0.60460200	-1.52553100
H	7.18532700	-1.01155000	-2.44448900
C	7.61782500	0.77662800	-1.38757200
H	7.22493100	1.38522900	-2.19707100
C	7.13658900	-2.75039900	-0.35740600
C	6.50504100	-3.30591800	-1.48116900
H	6.83700200	-3.04287600	-2.48096000
C	5.34692600	-4.06258900	-1.34857300
H	4.79993900	-4.33113200	-2.24776200
C	4.79973400	-4.33699900	-0.08697100
C	5.59670600	-4.03092100	1.02498100
H	5.27288800	-4.31756400	2.02138000
C	6.72958900	-3.23873800	0.89389900
H	7.21816600	-2.89327600	1.79950100
C	3.35471500	-4.65035800	0.06217100
C	2.67223000	-4.17238800	1.19090300
C	1.28505900	-4.16695200	1.25003200
H	0.78832100	-3.72370100	2.10953000
C	0.51958900	-4.61631200	0.16989300
C	1.19337600	-5.16789000	-0.92737200
H	0.61913400	-5.56776500	-1.75993700
C	2.58405700	-5.19692200	-0.97434700
H	3.22579700	-3.70894000	2.00113500
H	3.07224800	-5.62300800	-1.84677000
H	-1.33763600	5.76637000	1.67280600
H	-0.72186400	3.00419300	-1.55926400
C	-7.40015100	-4.81165800	0.22875500
C	-8.03681400	-3.62468500	0.05156100
C	-7.29179300	-2.40036200	-0.03183100
C	-5.84640300	-2.43095300	0.06048000
C	-5.20492600	-3.72634800	0.19791200
C	-5.97229500	-4.85471500	0.29702500
C	-7.94304400	-1.18603100	-0.17464200
C	-5.14667800	-1.22680300	0.05124800
C	-5.80617700	0.01972500	-0.06694000
C	-7.24019300	0.03168600	-0.20140800
C	-7.90436400	1.26452100	-0.32243900
H	-8.98918400	1.27137800	-0.41285800
C	-7.21884500	2.46842000	-0.31158600
C	-5.77516400	2.47415200	-0.17512100
C	-5.11443700	1.25441400	-0.04646600
H	-9.02893200	-1.16897000	-0.24973600
H	-7.96325100	-5.73742600	0.30104600
H	-9.12052900	-3.57347000	-0.01987500
H	-5.47793000	-5.81767100	0.39749700
H	-4.06363900	-1.23558800	0.15248600
C	-5.85049800	4.91169000	-0.23297000

C	-1.47926400	4.46662800	-0.18673100
C	-2.03177900	3.56315900	-1.10716200
C	-3.39123400	3.27260900	-1.10968600
H	-3.79245200	2.58623000	-1.85133000
C	-0.01643200	4.71191300	-0.17342400
C	0.73937000	4.60879100	-1.34762800
H	0.23722700	4.49159500	-2.30430500
C	2.12824300	4.59901200	-1.30580800
H	2.68318500	4.42560800	-2.22388900
C	2.81784500	4.70331000	-0.09020200
C	2.06285400	4.92613900	1.06943500
H	2.56642200	5.04099000	2.02559800
C	0.67338100	4.92628900	1.02805800
H	0.11976700	5.01546700	1.95932900
C	4.25156000	4.32513900	-0.03809600
C	4.68543100	3.50795400	1.01121100
H	4.04078500	3.30517100	1.86122000
C	5.85193200	2.76187600	0.89308200
H	6.05530000	2.01638300	1.65719100
C	6.61817900	2.80304800	-0.27920200
C	6.28915500	3.77149500	-1.24087800
H	6.90386700	3.87784600	-2.13187000
C	5.12400700	4.52034300	-1.12155900
H	4.85244600	5.21251500	-1.91515300
C	7.40372300	1.58481300	-0.60455100
C	8.01317800	0.78737500	0.37672600
H	8.31276800	1.23088000	1.32328500
C	8.09443400	-0.59283500	0.21858400
H	8.46689000	-1.18772000	1.04775300
C	7.54610600	-1.22166800	-0.91374900
C	7.22884000	-0.39114300	-1.99852700
H	6.84186900	-0.81624500	-2.91824200
C	7.16276800	0.98787200	-1.84879200
H	6.72724300	1.57676600	-2.65142700
C	6.87800500	-2.55179400	-0.82657700
C	6.23643900	-3.11868800	-1.93961800
H	6.54818900	-2.85351500	-2.94532100
C	5.09097100	-3.89008600	-1.78617300
H	4.53552900	-4.17187600	-2.67613900
C	4.56751200	-4.16806600	-0.51428400
C	5.37607600	-3.84639000	0.58455800
H	5.06726800	-4.12148100	1.58844200
C	6.49695400	-3.04096500	0.43234500
H	6.98676700	-2.68004300	1.33145900
C	3.13180100	-4.50661400	-0.33832100
C	2.46896700	-4.05408800	0.81231300
C	1.08475600	-4.08856200	0.90664400
H	0.60335500	-3.66361200	1.78457700
C	0.30321700	-4.54715900	-0.15807800
C	0.96101900	-5.06626300	-1.28074300
H	0.37547200	-5.47183000	-2.10260800
C	2.34993200	-5.05955300	-1.36332000
H	3.02596200	-3.58051300	1.61343000
H	2.82573300	-5.46504300	-2.25218700
H	-1.95052300	5.77106300	1.46520000
H	-1.38510100	3.06239200	-1.82299100
C	-7.57055400	-5.02104500	0.12324600
C	-8.27231400	-3.86729700	-0.02578900
C	-7.59385300	-2.60545400	-0.11824600
C	-6.14659500	-2.56245600	-0.06804200
C	-7.27328800	4.89374100	-0.36127900
C	-7.93478800	3.70843000	-0.40736600
C	-5.10498700	3.76546300	-0.14875200
H	-5.33776900	5.87005800	-0.23263300
H	-7.81196800	5.83390200	-0.43331700
H	-9.01627300	3.67115700	-0.51254800
H	-4.03634500	1.23617800	0.09118000
C	-0.95014600	-4.41253900	0.17392600
C	-1.72355100	-4.73441000	1.29433400
C	-1.58454900	-3.81649200	-0.92423600
C	-3.09236200	-4.48256300	1.31019900
H	-1.24984600	-5.20370100	2.15321400
C	-2.94919200	-3.54649700	-0.90064700
H	-0.99417800	-3.53950300	-1.79416000
C	-3.72520300	-3.88536900	0.21448400
H	-3.68420100	-4.75151900	2.18146000
H	-3.42661700	-3.08418600	-1.76154700

[TET_7R1.GA]
E = -3106.715923

C	4.45128000	-3.72167200	-0.20929800
C	3.86506100	-4.59145700	0.71857900
H	4.47186800	-4.98733000	1.52899200
C	2.52254400	-4.94229600	0.62811300
C	1.71250200	-4.43218700	-0.39334500
C	2.30805100	-3.57868300	-1.33473100
C	3.64983700	-3.22745300	-1.24540800
H	4.08556500	-2.56584200	-1.99026500
C	0.24696200	-4.66352400	-0.41592700
C	-0.48883400	-4.60379500	-1.60673000
H	0.02932300	-4.55041600	-2.56072900
C	-1.87841300	-4.55115900	-1.58853100
H	-2.40637100	-4.41447600	-2.52791700
C	-2.59431800	-4.58361000	-0.38183500
C	-1.85791300	-4.76379200	0.79760000
H	-2.36419800	-4.82608400	1.75607200
C	-0.46911000	-4.78947000	0.78188200
H	0.05859200	-4.82373200	1.73108200
C	-4.03549800	-4.22368500	-0.35222100
C	-4.55702500	-3.56643700	0.76856600
H	-3.96152800	-3.47283200	1.67104000
C	-5.74984600	-2.85929700	0.69222300
H	-6.03830400	-2.23780300	1.53554100
C	-6.47006900	-2.77977500	-0.50771800
C	-6.05025900	-3.59799700	-1.56798200
H	-6.62555900	-3.62374300	-2.49029700
C	-4.85711200	-4.30784100	-1.48939400
H	-4.53411100	-4.88833500	-2.34953100
C	-7.34683300	-1.59659300	-0.69908200
C	-8.01834000	-0.97407900	0.36582200
H	-8.27670900	-1.54922900	1.25160300
C	-8.22058700	0.40132200	0.36829700
H	-8.63706700	0.86327600	1.25907500
C	-7.73794100	1.20121600	-0.68325400
C	-7.37253700	0.53110500	-1.85986700
H	-7.03924400	1.09353500	-2.72618000
C	-7.17715800	-0.84449500	-1.86778600
H	-6.70373800	-1.29650800	-2.73536400
C	-7.16111400	2.55410600	-0.43660100

C	-5.43632800	-3.82284700	0.04227100	C	-6.59613400	3.31259600	-1.47480100
C	-6.14115500	-4.99021300	0.15291700	H	-6.93611700	3.17788200	-2.49775600
C	-8.31027700	-1.42490900	-0.22862100	C	-5.47948400	4.10982300	-1.24658400
C	-5.50897200	-1.32512700	-0.08818000	H	-4.96626300	4.54670300	-2.09949600
C	-6.23515300	-0.11372800	-0.17066900	C	-4.91408400	4.21082700	0.03349700
C	-7.67137200	-0.17259100	-0.25998400	C	-5.66441500	3.70152900	1.10086300
C	-8.40030900	1.02689100	-0.34034400	H	-5.31578200	3.83848300	2.12069300
H	-9.48656400	0.98006000	-0.39428100	C	-6.75722400	2.87718600	0.86873500
C	-7.77564700	2.26365500	-0.33179400	H	-7.19594700	2.36270900	1.71792000
C	-6.33099400	2.33896100	-0.24483200	C	-3.47499200	4.53251800	0.20278600
C	-5.60619000	1.15294100	-0.15528500	C	-2.74238200	3.78936100	1.13978600
H	-9.39734600	-1.46304500	-0.27182100	C	-1.35512800	3.79897100	1.14207800
H	-8.08261800	-5.97523800	0.20431000	H	-0.84973200	3.14287900	1.84267600
H	-9.35877000	-3.87165400	-0.06628000	C	-0.63540000	4.54566300	0.20408100
H	-5.59508800	-5.92678200	0.23319500	C	-1.36580400	5.36630700	-0.67016600
H	-4.42326300	-1.27902300	-0.02462200	H	-0.83851500	6.01093300	-1.36885700
C	-6.52373100	4.77062800	-0.25695000	C	-2.75717700	5.36041800	-0.67224800
C	-7.94782100	4.68471300	-0.33206000	H	-3.24001500	3.10803000	1.82351800
C	-8.55296800	3.46929300	-0.37865600	H	-3.29016200	5.99053500	-1.38032600
C	-5.72293100	3.66004300	-0.22339300	H	2.10015300	-5.62077000	1.36418600
H	-6.05789400	5.75275400	-0.25957500	H	1.70321900	-3.15027000	-2.12999800
H	-8.53419000	5.59805600	-0.36475500	C	7.16373800	5.36218100	-0.49932800
H	-9.63456900	3.38187400	-0.44625300	C	7.94533400	4.25274000	-0.44143100
H	-4.52442500	1.18628300	-0.05309600	C	7.35695000	2.94921000	-0.31596300
C	-1.16878700	-4.37972200	-0.11096600	C	5.91725900	2.81544500	-0.23363500
C	-1.89278600	-4.69134200	1.04426300	C	5.12310200	4.02794600	-0.27035000
C	-1.85309500	-3.81142900	-1.19372900	C	5.74108700	5.23958400	-0.41244500
C	-3.26535800	-4.46930400	1.10440600	C	8.15186400	1.81488300	-0.29044700
H	-1.37871100	-5.13257700	1.89474800	C	5.36179900	1.54084100	-0.15751500
C	-3.22148000	-3.56855600	-1.12595200	C	6.16566700	0.37571400	-0.15967500
H	-1.29834700	-3.53274500	-2.08681100	C	7.59742800	0.52473000	-0.21381600
C	-3.95061500	-3.91179300	0.01947600	C	8.40726300	-0.62476000	-0.19179600
H	-3.82122500	-4.73797600	1.99907400	H	9.48927600	-0.50615900	-0.20355900
H	-3.73905900	-3.13026600	-1.97637300	C	7.86569300	-1.89937400	-0.15212600
P	3.45389900	-0.20678100	1.73788200	C	6.42681500	-2.06691400	-0.14685400
O	3.73124400	0.98222900	2.56591700	C	5.62158300	-0.93007200	-0.12654100
O	1.91535000	-0.55808000	1.51576100	H	9.23390400	1.92087600	-0.34539600
F	3.94212500	-1.55752300	2.42434600	H	7.60686400	6.34808700	-0.60295500
C	4.25683400	-0.30240800	0.13908100	H	9.02869900	4.32519400	-0.49820300
H	4.00205900	-1.24453000	-0.35904000	H	5.13040200	6.13839500	-0.44106400
H	3.93279100	0.54833300	-0.46970500	H	4.27953700	1.43310100	-0.11323900
H	5.34089100	-0.24139000	0.27685900	C	6.77364600	-4.48070100	-0.07357600
C	0.41850500	0.65309800	-0.76567100	C	8.19118000	-4.30269300	-0.04854200
C	-0.93244100	-0.04075000	-0.55852400	C	8.71954000	-3.05214700	-0.10157000
C	-0.86432200	-1.00094000	0.63114400	C	5.90512300	-3.42418800	-0.13496900
C	-0.42601600	-0.27870000	1.90737600	H	6.37200300	-5.49076200	-0.08037500
C	0.89047400	0.46305200	1.71544300	H	8.83535000	-5.17589200	-0.00854000
C	0.84423500	1.39083200	0.50565800	H	9.79543000	-2.89591000	-0.10295400
H	-1.83147700	-1.49441900	0.79209700	H	4.54061600	-1.03776900	-0.07911700
H	-1.70387200	0.72315100	-0.37464700	C	0.83674900	4.39122500	0.09168900
H	-1.22874000	-0.58591000	-1.46338900	C	1.62844900	4.07133000	1.20501200
H	1.17492900	-0.10019300	-1.02870200	C	1.47169500	4.49779800	-1.15240000
H	0.37254900	1.36288800	-1.60086500	C	3.00114800	3.87849600	1.08057500
H	-1.18281100	0.46462700	2.19488200	H	1.16970300	4.00075200	2.18807400
H	-0.32664000	-0.98222600	2.74188200	C	2.84828100	4.33021600	-1.27376600
H	1.15887600	1.02394100	2.61607000	H	0.88126000	4.70336300	-2.04118700
H	0.11544100	2.18196600	0.72771600	C	3.63533500	4.02783400	-0.15865100
H	1.81354900	1.89557700	0.37998000	H	3.59903600	3.65494400	1.96112500
H	-0.14155000	-1.79368100	0.40307100	H	3.32453900	4.44067800	-2.24480900
				P	-1.71756800	-0.35101100	1.69378000

[TET_7R2.GD]
E = -5659.770353

C	2.75681100	-3.92742100	-0.36860300
C	3.37931100	-4.61116700	-1.42030000
H	2.77969700	-4.93912300	-2.26592600
C	4.74481100	-4.87599600	-1.39695400
C	5.53922600	-4.46388700	-0.32093000
C	4.91266300	-3.77781500	0.73108500
C	3.54890500	-3.51125700	0.70836600
H	3.08596400	-2.98846900	1.54211900
C	7.00848500	-4.66211800	-0.31282600
C	7.71114600	-4.78655900	0.89150700
H	7.16654200	-4.87074600	1.82812600
C	9.09927800	-4.74992000	0.91699700
H	9.61006500	-4.75664500	1.87601400
C	9.84390900	-4.60553700	-0.26178300
C	9.14412700	-4.60179000	-1.47617700
H	9.69055900	-4.52269000	-2.41220500
C	7.75390000	-4.62224300	-1.49975800
H	7.24287200	-4.53646800	-2.45546900
C	11.27523000	-4.22434300	-0.17638400
C	11.76277500	-3.25132500	-1.05540100
H	11.16552500	-2.90888300	-1.89499400
C	12.92193000	-2.54496100	-0.75906600
H	13.16949700	-1.69219700	-1.38558500
C	13.62367000	-2.77669100	0.43144600
C	13.24602500	-3.88525400	1.20564500
H	13.81249600	-4.13848400	2.09887600
C	12.09169700	-4.59939600	0.90348700
H	11.78398200	-5.41252600	1.55635700
C	14.38150600	-1.62503000	0.98576800
C	15.04807900	-0.69212000	0.17611300
H	15.41335200	-0.99209700	-0.80315100
C	15.10308400	0.65023100	0.53933400
H	15.52328700	1.35882300	-0.16833600
C	14.47012800	1.10712800	1.71010800
C	14.09558500	0.12532700	2.63899500
H	13.64924800	0.40862100	3.58593400
C	14.05474200	-1.21722300	2.28539000
H	13.57723700	-1.91870200	2.96395600
C	13.78474700	2.43167900	1.76255100
C	13.01499700	2.80745300	2.87620800
H	13.22593100	2.38940200	3.85571200
C	11.86687700	3.57612900	2.72435700
H	11.21083600	3.70220300	3.58139300
C	11.46759900	4.04090200	1.46252500
C	12.39637000	3.91402000	0.42033900
H	12.18130900	4.33915800	-0.55576300
C	13.52231900	3.11387500	0.56375000
H	14.11406100	2.91134900	-0.32349500
C	10.04367600	4.35865000	1.18478200
C	9.52029500	4.01745800	-0.07068900
C	8.15329100	4.01803100	-0.30472500
H	7.78360200	3.67114700	-1.26603500
C	7.24863700	4.33969700	0.71053600
C	7.76715700	4.74642800	1.94788400
H	7.08564100	5.04567700	2.74080800
C	9.13994200	4.76556400	2.17774400
H	10.17576000	3.65408300	-0.85459000
O	-2.84474300	-1.08045500	0.84340200
O	-2.02329600	0.88662700	2.43793000
C	-3.35330800	-0.52327600	-0.39495300
H	-2.50517700	-0.16637000	-0.99060300
H	-3.81355700	-1.37570800	-0.90422200
C	-4.34972400	0.58099900	-0.11666900
H	-3.88432100	1.38333200	0.46307500
H	-4.71162300	1.00490500	-1.05888300
H	-5.21089500	0.19306700	0.43905100
N	-0.41380000	-0.23856700	0.69604400
C	-0.00458400	-1.39021700	-0.10203300
H	0.77678000	-1.96364400	0.41409900
H	-0.85501600	-2.05039000	-0.29526200
H	0.39459800	-1.03869500	-1.06058700
C	0.66393200	0.70395500	0.98713100
H	0.93571700	1.24917400	0.07591300
H	0.33439600	1.42411900	1.73529500
H	1.54687700	0.17211800	1.36631200
C	-1.41817000	-1.75064400	2.81086300
N	-1.15931600	-2.64791400	3.49413800

[TET_7R1.GD]
E = -3176.328283

C	-4.35860600	3.81841200	-0.29906100
C	-3.80501000	4.61898100	0.70691500
H	-4.45303800	5.00847700	1.48803300
C	-2.44706500	4.91925200	0.72041600
C	-1.59105800	4.42640800	-0.27106200
C	-2.15037300	3.63148000	-1.28296200
C	-3.50740500	3.32989800	-1.29719700
H	-3.91639800	2.71602900	-2.09637100
C	-0.12686600	4.65342600	-0.21700700
C	0.64884000	4.66537300	-1.38253100
H	0.16369100	4.63695800	-2.35455100
C	2.03643200	4.65777000	-1.31860000
H	2.60462700	4.57517200	-2.24088100
C	2.70848400	4.65971200	-0.08826800
C	1.93218600	4.76528500	1.07421300
H	2.41717900	4.80209300	2.04583900
C	0.54360700	4.75330900	1.01014200
H	-0.02459800	4.75499000	1.93669500
C	4.15256600	4.31977900	-0.04331100
C	4.62210100	3.49758700	0.98658200
H	3.99329600	3.25280400	1.83663200
C	5.81500100	2.79834900	0.84752200
H	6.05462400	2.04864500	1.59724100
C	6.57219400	2.88809200	-0.32791900
C	6.20585200	3.86731100	-1.26463000
H	6.81151400	4.01623900	-2.15576400
C	5.01693600	4.57353800	-1.12149200
H	4.72248700	5.27777400	-1.89578700
C	7.38707800	1.69795500	-0.68479400
C	8.03339300	0.89957900	0.27201300
H	8.33993900	1.33471800	1.22028900
C	8.14512100	-0.47575800	0.08924400
H	8.54695000	-1.07543300	0.90126400
C	7.59119900	-1.09922300	-1.04349400
C	7.23473900	-0.25874500	-2.10829400
H	6.84117900	-0.67797800	-3.02792800

H	9.50841500	5.07938100	3.15114900	C	7.13856900	1.11556700	-1.93444200
H	5.19481700	-5.42298100	-2.22130600	H	6.67435300	1.70691900	-2.71900100
H	5.50421700	-3.42509700	1.57185200	C	6.95266000	-2.44465900	-0.96852100
C	5.79625100	4.16048700	0.47671800	C	6.31126200	-3.00897400	-2.08311100
C	5.20863400	4.56477400	-0.72703200	H	6.61203300	-2.72833700	-3.08800300
C	4.99299000	3.50884600	1.42166600	C	5.17571800	-3.79509700	-1.92938700
C	3.85444600	4.35329400	-0.96507600	H	4.61479900	-4.07295800	-2.81729800
H	5.81381100	5.07311100	-1.47380700	C	4.66447800	-4.08960700	-0.65646400
C	3.64227200	3.28206900	1.17740900	C	5.47943400	-3.77438600	0.43975600
H	5.43929600	3.15527600	2.34831800	H	5.17934700	-4.06175400	1.44299700
C	3.04778100	3.71931400	-0.01319800	C	6.59015100	-2.95474600	0.28761000
H	3.40789800	4.69323700	-1.89627700	H	7.08257200	-2.59658100	1.18658800
H	3.03134000	2.78529900	1.92771400	C	3.22967900	-4.42812500	-0.47296400
C	-2.75598000	3.93083600	-0.36507300	C	2.57528700	-3.96092200	0.67610300
C	-3.37870700	4.61468600	-1.41675900	C	1.19213800	-3.98961500	0.77870900
H	-2.77908000	4.94348200	-2.26213400	H	0.71847900	-3.54932100	1.65205800
C	-4.74443900	4.87792400	-1.39389700	C	0.40175300	-4.46440900	-0.27105200
C	-5.53893300	4.46414400	-0.31853500	C	1.05104200	-4.99479200	-1.39390600
C	-4.91206000	3.77895500	0.73404200	H	0.45937600	-5.41153800	-2.20570900
C	-3.54803500	3.51394500	0.71190900	C	2.43959900	-4.98760700	-1.48820300
H	-3.08480500	2.99155200	1.54590100	H	3.13932100	-3.48254800	1.46946000
C	-7.00842400	4.66021900	-0.31200200	H	2.90914000	-5.40067900	-2.37705700
C	-7.71263700	4.78481200	0.89142100	H	-2.05147900	5.55584500	1.50740300
H	-7.16926500	4.86949300	1.82875200	H	-1.51011300	3.21542200	-2.05627100
C	-9.10090500	4.74814500	0.91499200	C	-7.45218100	-5.12261900	0.12242000
H	-9.61323300	4.75507600	1.87325100	C	-8.18453200	-3.99030700	-0.04423100
C	-9.84384500	4.60373800	-0.26491500	C	-7.53942600	-2.71352800	-0.16728200
C	-9.14232500	4.59897600	-1.47829300	C	-6.09372100	-2.63362100	-0.12962300
H	-9.68763000	4.51940100	-2.41495900	C	-5.35020700	-3.87293400	-0.00118000
C	-7.75213300	4.61922500	-1.49991500	C	-6.02375400	-5.05543100	0.13885100
H	-7.23963800	4.53294800	-2.45480200	C	-8.28609200	-1.55324800	-0.29299400
C	-11.27541900	4.22277300	-0.18145900	C	-5.48693600	-1.38174600	-0.17735900
C	-11.76106300	3.24833500	-1.05998100	C	-6.24392200	-0.19066100	-0.27469000
H	-11.16221500	2.90545800	-1.89811800	C	-7.67919100	-0.28566700	-0.34957100
C	-12.92026400	2.54183400	-0.76471600	C	-8.43958300	0.89430400	-0.43642700
H	-13.16646700	1.68821900	-1.39060600	H	-9.52466900	0.81910700	-0.48152200
C	-13.62399800	2.77473900	0.42431800	C	-7.84632200	2.14659900	-0.43872100
C	-13.24837200	3.88461400	1.19766200	C	-6.40411100	2.25622600	-0.36492200
H	-13.81644100	4.13883300	2.08953700	C	-5.64652600	1.09094900	-0.28082100
C	-12.09380800	4.59904200	0.89651400	H	-9.37222200	-1.61926200	-0.32522000
H	-11.78747400	5.41317700	1.54879700	H	-7.93921300	-6.08754200	0.22705800
C	-14.38214500	1.62325800	0.97843400	H	-9.27080900	-4.02343200	-0.07562400
C	-15.04732500	0.68951100	0.16869300	H	-5.45351700	-5.97638300	0.23122900
H	-15.41114800	0.98855100	-0.81137700	H	-4.40245500	-1.30747300	-0.11922100
C	-15.10245100	-0.65256800	0.53288700	C	-6.64898300	4.68289000	-0.36087100
H	-15.52121200	-1.36189100	-0.17493100	C	-8.07175300	4.56416000	-0.43074300
C	-14.47125800	-1.10833800	1.70507300	C	-8.65002900	3.33548500	-0.47944100
C	-14.09842400	-0.12553400	2.63367800	C	-5.82655700	3.58913400	-0.34220000
H	-13.65355900	-0.40779000	3.58162200	H	-6.20347000	5.67451400	-0.35161100
C	-14.05728300	1.21672800	2.27889600	H	-8.67924300	5.46387100	-0.45494800
H	-13.58097600	1.91894300	2.95759700	H	-9.72992900	3.22484600	-0.54031400
C	-13.78533800	-2.43259200	1.75974100	H	-4.56421300	1.15626900	-0.19624100
C	-13.01611800	-2.80648000	2.87440400	C	-1.07128200	-4.31506400	-0.20251500
H	-13.22791200	-2.38717700	3.85315800	C	-1.77095000	-4.62654700	0.96830700
C	-11.86751900	-3.57463400	2.72455700	C	-1.78554600	-3.78259700	-1.28369700
H	-11.21214600	-3.69911200	3.58229400	C	-3.14817500	-4.44098900	1.04453900
C	-11.46694800	-4.04111800	1.46376400	H	-1.23405000	-5.04304600	1.81739200
C	-12.39512700	-3.91616200	0.42070200	C	-3.15910200	-3.57772800	-1.20063100
H	-12.17923900	-4.34241400	-0.55471900	H	-1.25107500	-3.50361600	-2.18882800
C	-13.52162400	-3.11632700	0.56211900	C	-3.86307400	-3.92223100	-0.04027700

H	-14.11274800	-2.91543200	-0.32591300
C	-10.04263500	-4.35870100	1.18746600
C	-9.51899600	-4.02088900	-0.06878000
C	-8.15186100	-4.02133500	-0.30245200
H	-7.78219700	-3.67701100	-1.26462400
C	-7.24724500	-4.33939100	0.71390200
C	-7.76600300	-4.74268900	1.95222600
H	-7.08448100	-5.03895600	2.74621100
C	-9.13877800	-4.76203500	2.18171500
H	-10.17439400	-3.66019400	-0.85389500
H	-9.50724800	-5.07324500	3.15589900
H	-5.19462300	5.42479200	-2.21835600
H	-5.50351300	3.42529400	1.57462100
C	0.51024000	-4.88485300	-0.49369100
C	1.28010400	-3.76389300	-0.35961600
C	0.63201700	-2.47393200	-0.21104300
C	-0.81942500	-2.41824100	-0.15563700
C	-1.57417100	-3.65551500	-0.20654300
C	-0.90988400	-4.83230100	-0.40848600
C	1.35254700	-1.28522400	-0.18187200
C	-1.44742200	-1.17865500	-0.12370400
C	-0.71764800	0.02927700	-0.12545000
C	0.71829700	-0.02623700	-0.12580000
C	1.44800700	1.18163600	-0.12379000
H	2.53506100	1.13290800	-0.13332700
C	0.82007100	2.42121300	-0.15497600
C	-0.63137000	2.47705500	-0.20931400
C	-1.35201200	1.28834200	-0.18051700
H	2.43784200	-1.31286600	-0.24359100
H	0.99414300	-5.85166500	-0.60558400
H	-1.47324700	-5.76110500	-0.44494000
H	-2.53445100	-1.12983800	-0.13263300
C	-0.50931800	4.88814700	-0.49134500
C	0.91088100	4.83522100	-0.40751000
C	1.57494900	3.65823600	-0.20630200
C	-1.27934100	3.76725500	-0.35681700
H	-0.99308400	5.85509800	-0.60240500
H	1.47452100	5.76384600	-0.44439000
H	-2.43731700	1.31606800	-0.24175000
C	-5.79493400	-4.15990600	0.47966700
C	-5.20745500	-4.56554700	-0.72376600
C	-4.99167900	-3.50626200	1.42325400
C	-3.85352500	-4.35333100	-0.96274300
H	-5.81253500	-5.07545400	-1.46955800
C	-3.64123300	-3.27890700	1.17804300
H	-5.43788000	-3.15144100	2.34947400
C	-3.04687100	-3.71728300	-0.01225500
H	-3.40724100	-4.69429100	-1.89369400
H	-3.03044700	-2.78051100	1.92734200
P	-10.65487300	-0.51203100	-1.44990300
O	-10.95951200	0.56763900	-2.40918600
O	-9.11639000	-0.88447100	-1.28403700
C	-11.31138900	-0.37865800	0.21031500
H	-10.95503400	0.55361700	0.65870100
H	-12.40433800	-0.35006800	0.16168200
H	-10.99282100	-1.23695500	0.81247700
C	-8.06991200	-0.17147500	-2.00561800
C	-7.05552800	0.34604600	-0.96943700
F	-11.26142100	-1.90971800	-1.92421000
C	-6.36771700	-0.80372800	-0.22568200
H	-3.68427100	-4.71017100	1.95111700
H	-3.70048800	-3.16968800	-2.05127200
P	3.49295400	-0.22973100	1.71406100
O	3.67651100	0.96926000	2.55452300
O	1.98861500	-0.66400600	1.43397600
C	4.31885900	-0.28039500	0.12759600
H	3.99970400	0.58547100	-0.46083800
H	5.40027500	-0.21914300	0.28484200
H	4.07800100	-1.21037200	-0.39926600
C	0.85907800	0.05961100	2.00489200
C	-0.09746000	0.40870700	0.85083200
F	4.06713500	-1.53934800	2.42387500
C	-0.67102400	-0.85006900	0.19103400
H	0.13119000	-1.52285500	-0.13392600
H	-1.33538100	-1.40989500	0.86132400
H	-1.25985500	-0.57299000	-0.69232500
C	0.68580400	1.21329400	-0.19610800
H	1.22469700	2.05526900	0.26021000
H	1.40975300	0.57726500	-0.71667700
H	0.00041800	1.62619600	-0.94475700
C	-1.23676900	1.27360600	1.40654600
H	-1.88979700	1.60266300	0.59031500
H	-1.85436600	0.72307200	2.12437400
H	-0.85056300	2.17554100	1.89914500
C	0.28781200	-0.79482600	3.12305800
H	-0.10112300	-1.74219000	2.73834200
H	1.07026900	-1.01055400	3.85707700
H	-0.52238500	-0.27010500	3.63681200
H	1.24721200	0.99684400	2.42373700

TET_7R2
E = -3923.942072

C	3.80152100	2.97408500	-0.87211000
C	4.57847300	3.62772000	-1.83800700
H	5.00616800	3.04920400	-2.65305700
C	4.82120200	4.99529200	-1.76512600
C	4.28724400	5.76502500	-0.72565500
C	3.48583700	5.11498200	0.22445200
C	3.25022400	3.74662700	0.15717900
H	2.65214100	3.26594500	0.92751200
C	4.51272200	7.22840400	-0.63233200
C	4.57375700	7.86080900	0.61531100
H	4.57072600	7.26161900	1.52176900
C	4.58327600	9.24567700	0.71693000
H	4.52980500	9.70078600	1.70182700
C	4.55161500	10.05830100	-0.42522400
C	4.61782800	9.42341500	-1.67317000
H	4.63476100	10.02050600	-2.58119700
C	4.58994700	8.03658600	-1.77488900
H	4.55984700	7.57911100	-2.76049700
C	4.18223800	11.48942500	-0.27901600
C	3.28741100	12.05382700	-1.19613400
H	3.05820600	11.53030300	-2.11965000
C	2.52949200	13.16771300	-0.85806300
H	1.72553500	13.47303100	-1.52170200
C	2.63431800	13.75284000	0.41150100
C	3.69334000	13.33233400	1.23040400
H	3.85836200	13.81655200	2.19004300
C	4.45537200	12.22001900	0.88856900

H	-7.10506000	-1.46517600	0.24383000
H	-5.73437900	-1.41157100	-0.88373900
H	-5.72150300	-0.40150600	0.56479000
C	-7.80523700	1.22040100	0.04548800
H	-8.41617700	1.98734500	-0.45099100
H	-8.45702700	0.61131800	0.68074400
H	-7.09266600	1.73835700	0.69689500
C	-6.00700600	1.19896100	-1.69492300
H	-5.31641200	1.64200700	-0.96858900
H	-5.41261900	0.60550300	-2.39767300
H	-6.47536000	2.02434100	-2.24627500
C	-7.53086800	-1.11192100	-3.06956600
H	-7.06552500	-1.99467100	-2.62074200
H	-8.34939700	-1.44037500	-3.71704300
H	-6.78787500	-0.60566600	-3.69178500
H	-8.53717200	0.69572800	-2.48952700
P	10.65452100	0.51116600	-1.45453500
O	10.96219000	-0.56669500	-2.41505000
O	9.11547600	0.88178600	-1.29058700
C	11.30881700	0.37614300	0.20636800
H	10.95302700	-0.55727000	0.65296700
H	12.40187600	0.34904600	0.15925100
H	10.98831000	1.23320200	0.80926900
C	8.07077800	0.16973900	-2.01537900
C	7.05217500	-0.34659300	-0.98255600
F	11.26031000	1.91031200	-1.92576700
C	6.36076200	0.80380500	-0.24288200
H	7.09581800	1.46557600	0.22972700
H	5.73042900	1.41108000	-0.90427400
H	5.71091200	0.40191600	0.54472900
C	7.79835400	-1.21924000	0.03652000
H	8.40981600	-1.98793800	-0.45684400
H	8.44922700	-0.60914800	0.67207900
H	7.08327900	-1.73480500	0.68743100
C	6.00698000	-1.20071600	-1.71125400
H	5.31439600	-1.64445300	-0.98713000
H	5.41423900	-0.60799600	-2.41613900
H	6.47791400	-2.02570800	-2.26103800
C	7.53605800	1.10991200	-3.08187000
H	7.06962700	1.99330800	-2.63537500
H	8.35724000	1.43735900	-3.72654300
H	6.79503200	0.60359000	-3.70655400
H	8.53858100	-0.69834300	-2.49736200

6CPP_1R
E = -1616.679299

C	5.46831300	0.57760300	-0.02235900
C	6.48979200	-1.61533100	-0.00193900
C	7.89038800	0.34183200	0.04663100
C	5.33023000	-0.82896200	-0.04469700
C	7.75767400	-1.04416400	0.04771000
C	6.74940300	1.13847000	0.01161500
C	4.28854500	1.48943200	-0.00547000
C	4.04485000	2.37681000	-1.06042100
H	4.75735000	2.43801500	-1.88022700
C	2.86761000	3.11985100	-1.10920900
C	1.91646500	3.02011700	-0.08594300
C	2.21995000	2.21884000	1.02233500
C	3.37923100	1.45174100	1.05443700
H	5.21464500	11.86483600	1.58084400
C	1.40664400	14.39842400	0.94690200
C	0.49015500	15.08521100	0.13551100
H	0.83183300	15.56374700	-0.77946700
C	-0.87351100	15.02850500	0.40277600
H	-1.56194200	15.47815400	-0.30740800
C	-1.36621000	14.26756400	1.47776000
C	-0.42848900	13.85358600	2.43417400
H	-0.74809500	13.29731700	3.30862900
C	0.93396900	13.92134500	2.17610500
H	1.61537000	13.41313100	2.85256700
C	-2.66881600	13.54683800	1.40737400
C	-3.12612100	12.75339000	2.47116100
H	-2.78382400	12.94629100	3.48348200
C	-3.88568300	11.61349800	2.23677300
H	-4.07868800	10.94092600	3.06773800
C	-4.25952400	11.24513300	0.93627100
C	-4.04714800	12.19419200	-0.07334500
H	-4.41098300	12.00788400	-1.07983200
C	-3.25403300	13.31123000	0.15353900
H	-2.98386000	13.92735400	-0.69821400
C	-4.58093000	9.83122200	0.61174300
C	-4.18223800	9.31500900	-0.63032300
C	-4.18223700	7.94915100	-0.88041300
H	-3.80037500	7.57910900	-1.82860200
C	-4.55827500	7.03853400	0.11141300
C	-5.03285700	7.55025100	1.32646800
H	-5.37818300	6.86352400	2.09568000
C	-5.05607100	8.92072300	1.56713300
H	-3.77650700	9.98018600	-1.38562300
H	-5.42102600	9.28214700	2.52478600
H	5.45338400	5.46474700	-2.51460500
H	3.02752300	5.69169000	1.02316300
C	-4.36300600	5.58459900	-0.10944200
C	-4.75191200	4.97363200	-1.30624200
C	-3.71228600	4.80320700	0.85470100
C	-4.51520500	3.61948900	-1.52412000
H	-5.26258100	5.56111700	-2.06548600
C	-3.45789600	3.45442700	0.62900700
H	-3.38173900	5.26663000	1.78114700
C	-3.86683700	2.83903800	-0.56062900
H	-4.83722900	3.15501600	-2.45293200
H	-2.95456600	2.86278800	1.39022900
C	-3.80152100	-2.97408500	-0.87211000
C	-4.57847300	-3.62772000	-1.83800700
H	-5.00616800	-3.04920400	-2.65305700
C	-4.82120200	-4.99529200	-1.76512600
C	-4.28724400	-5.76502500	-0.72565500
C	-3.48583700	-5.11498200	0.22445200
C	-3.25022400	-3.74662700	0.15717900
H	-2.65214100	-3.26594500	0.92751200
C	-4.51272200	-7.22840400	-0.63233200
C	-4.57375700	-7.86080900	0.61531100
H	-4.57072600	-7.26161900	1.52176900
C	-4.58327600	-9.24567700	0.71693000
H	-4.52980500	-9.70078600	1.70182700
C	-4.55161500	-10.05830100	-0.42522400
C	-4.61782800	-9.42341500	-1.67317000
H	-4.63476100	-10.02050600	-2.58119700
C	-4.58994700	-8.03658600	-1.77488900

H	3.55648800	0.77413900	1.88676900	H	-4.55984700	-7.57911100	-2.76049700
C	0.52480500	3.51603100	-0.24213800	C	-4.18223800	-11.48942500	-0.27901600
C	-0.22540600	4.05649000	0.81103600	C	-3.28741100	-12.05382700	-1.19613400
H	0.27939100	4.37372900	1.72081700	H	-3.05820600	-11.53030300	-2.11965000
C	-1.61582700	4.10785100	0.74717100	C	-2.52949200	-13.16771300	-0.85806300
H	-2.16404800	4.47205400	1.61195200	H	-1.72553500	-13.47303100	-1.52170200
C	-2.30987400	3.59531500	-0.36140800	C	-2.63431800	-13.75284000	0.41150100
C	-1.54140000	3.22695700	-1.47674900	C	-3.69334000	-13.33233400	1.23040400
H	-2.02357100	2.81685400	-2.35754100	H	-3.85836200	-13.81655200	2.19004300
C	-0.15515700	3.19617800	-1.42205200	C	-4.45537200	-12.22001900	0.88856900
H	0.39720800	2.78025400	-2.26116000	H	-5.21464500	-11.86483600	1.58084400
C	-3.70667600	3.08639300	-0.27482300	C	-1.40664400	-14.39842400	0.94690200
C	-4.36978800	2.58816000	-1.40799400	C	-0.49015500	-15.08521100	0.13551100
H	-4.10863100	2.94830600	-2.39872600	H	-0.83183300	-15.56374700	-0.77946700
C	-5.22006500	1.49469500	-1.30688700	C	0.87351100	-15.02850500	0.40277600
H	-5.55181200	1.02443700	-2.22730500	H	1.56194200	-15.47815400	-0.30740800
C	-5.46838800	0.88102700	-0.06648900	C	1.36621000	-14.26756400	1.47776000
C	-5.12376600	1.63636600	1.06695600	C	0.42848900	-13.85358600	2.43417400
H	-5.39234300	1.29116100	2.06073400	H	0.74809500	-13.29731700	3.30862900
C	-4.23784300	2.70303500	0.96556400	C	-0.93396900	-13.92134500	2.17610500
H	-3.83303200	3.11668100	1.88486600	H	-1.61537000	-13.41313100	2.85256700
C	-5.54789600	-0.60840900	0.00816800	C	2.66881600	-13.54683800	1.40737400
C	-5.57474000	-1.40003500	-1.15611000	C	3.12612100	-12.75339000	2.47116100
H	-6.05078400	-1.03181900	-2.06029700	H	2.78382400	-12.94629100	3.48348200
C	-4.83140900	-2.57137500	-1.23766900	C	3.88568300	-11.61349800	2.23677300
H	-4.75976000	-3.07449500	-2.19831700	H	4.07868800	-10.94092600	3.06773800
C	-4.03733400	-2.99474100	-0.15724900	C	4.25952400	-11.24513300	0.93627100
C	-4.32758600	-2.41160100	1.08370900	C	4.04714800	-12.19419200	-0.07334500
H	-3.80336000	-2.73747800	1.97649100	H	4.41098300	-12.00788400	-1.07983200
C	-5.07176400	-1.24256400	1.16569100	C	3.25403300	-13.31123000	0.15353900
H	-5.08912200	-0.73034300	2.12163000	H	2.98386000	-13.92735400	-0.69821400
C	-2.68719400	-3.59265500	-0.35324300	C	4.58093000	-9.83122200	0.61174300
C	-1.88312500	-3.98176000	0.73068900	C	4.18223800	-9.31500900	-0.63032300
H	-2.33922700	-4.24890500	1.68002700	C	4.18223700	-7.94915100	-0.88041300
C	-0.49734100	-3.87954600	0.66525400	H	3.80037500	-7.57910900	-1.82860200
H	0.07852500	-4.05098800	1.57116300	C	4.55827500	-7.03853400	0.11141300
C	0.14493500	-3.41834400	-0.49404800	C	5.03285700	-7.55025100	1.32646800
C	-0.64441100	-3.29100200	-1.64443900	H	5.37818300	-6.86352400	2.09568000
H	-0.18141700	-3.02903100	-2.59177700	C	5.05607100	-8.92072300	1.56713300
C	-2.02958200	-3.36385800	-1.57267700	H	3.77650700	-9.98018600	-1.38562300
H	-2.60236000	-3.09654400	-2.45511100	H	5.42102600	-9.28214700	2.52478600
C	1.52725100	-2.87630500	-0.44905400	H	-5.45338400	-5.46474700	-2.51460500
C	1.85833400	-1.77758200	-1.25850400	H	-3.02752300	-5.69169000	1.02316300
C	3.07291300	-1.11744800	-1.12755500	C	4.85897200	0.77807700	-0.99823500
H	3.28667900	-0.26480900	-1.76594900	C	3.69769000	1.49121800	-0.88634400
C	4.01334900	-1.51861700	-0.17041000	C	2.43979000	0.77326300	-0.76659500
C	3.69714200	-2.62992900	0.62262700	C	2.46270500	-0.68018700	-0.71293200
H	4.40690700	-2.96787600	1.37423800	C	3.73872600	-1.36922600	-0.74674800
C	2.48183600	-3.29801600	0.48447400	C	4.88139500	-0.64308100	-0.92507400
H	1.13164500	-1.39327800	-1.96821700	C	1.21048800	1.42294000	-0.75469200
H	2.27329800	-4.15242700	1.12341000	C	1.25956200	-1.37677500	-0.69593500
H	2.66697700	3.75838900	-1.96643100	C	0.01184100	-0.71809000	-0.70350700
H	1.49368000	2.12369700	1.82544100	C	-0.01184100	0.71809000	-0.70350700
H	6.38881400	-2.69694900	-0.04433900	C	-1.25956200	1.37677500	-0.69593500
H	8.63738100	-1.68064100	0.07133600	H	-1.27367300	2.46418100	-0.70576200
H	8.87391400	0.80140800	0.08003800	C	-2.46270500	0.68018700	-0.71293200
H	6.84104800	2.22173700	0.03583000	C	-2.43979000	-0.77326300	-0.76659500
C	-1.51129100	-0.11353500	0.18462100	C	-1.21048800	-1.42294000	-0.75469200
H	-0.79566700	0.08785400	-0.62254200	H	1.17430300	2.50708600	-0.82117400
H	-1.72373700	-1.18973700	0.18633300	H	5.80282900	1.31076700	-1.07775200

H	-2.44664200	0.41540300	-0.04745500
C	-0.94292300	0.31783700	1.53690700
H	-0.71234300	1.39196600	1.47729400
C	-1.96857100	0.10871800	2.65097400
H	-1.58560500	0.45379800	3.61872300
H	-2.90059400	0.64302300	2.43647700
H	-2.20677100	-0.95960100	2.74455900
C	0.34052800	-0.45013000	1.85770300
H	1.08866300	-0.34576800	1.06427300
H	0.78890300	-0.10552500	2.79883900
H	0.11883200	-1.51972400	1.96573000

Cyclohexanol
E = -310.951092

C	1.13812900	1.27273200	0.21302100
C	-0.33942600	1.24767300	-0.19161000
C	-1.02924700	-0.01512100	0.31975900
C	-0.31586100	-1.26284000	-0.18003000
C	1.16261900	-1.25100000	0.21946200
C	1.86209800	0.01652200	-0.27926000
H	1.62020200	2.17442800	-0.18099100
H	-0.85666000	2.13871100	0.19095100
H	-0.82515900	-2.15021300	0.21184300
H	1.66233200	-2.14367100	-0.17298100
H	2.90802500	0.02847900	0.04854100
O	-2.37714300	-0.09928500	-0.12177300
H	-2.84881200	0.69713000	0.16103600
H	-0.43152800	1.26473400	-1.28680900
H	1.21448900	1.32734600	1.30880200
H	1.87279200	0.01428900	-1.37870500
H	1.24482200	-1.30069200	1.31506000
H	-0.41182800	-1.29122300	-1.27458200
H	-1.00140500	-0.01284000	1.42396500

Ethanol
E = -154.961524

C	1.19132600	-0.78090800	-0.10275300
C	-0.00118200	0.04327200	0.36795600
H	0.00639200	0.09608000	1.46820600
H	2.13746600	-0.33291500	0.22376900
H	1.14677300	-1.79801200	0.30105100
H	1.19660800	-0.83690300	-1.19662000
C	-1.32187000	-0.54992600	-0.08908100
H	-2.15481400	0.07601100	0.24259800
H	-1.34564500	-0.60449600	-1.18272700
H	-1.45426700	-1.55756600	0.31711500
O	0.04710000	1.36313800	-0.16573600
H	0.88104700	1.77807400	0.09575700

[6CPP_1R. Isobutane]
E = -1775.062501

C	5.46831300	0.57760300	-0.02235900
C	6.48979200	-1.61533100	-0.00193900
C	7.89038800	0.34183200	0.04663100
C	5.33023000	-0.82896200	-0.04469700
C	7.75767400	-1.04416400	0.04771000
H	5.83940200	-1.15613300	-0.94956700
H	1.27367300	-2.46418100	-0.70576200
C	-4.85897200	-0.77807700	-0.99823500
C	-4.88139500	0.64308100	-0.92507400
C	-3.73872600	1.36922600	-0.74674800
C	-3.69769000	-1.49121800	-0.88634400
H	-5.80282900	-1.31076700	-1.07775200
H	-5.83940200	1.15613300	-0.94956700
H	-1.17430300	-2.50708600	-0.82117400
C	4.36300600	-5.58459900	-0.10944200
C	4.75191200	-4.97363200	-1.30624200
C	3.71228600	-4.80320700	0.85470100
C	4.51520500	-3.61948900	-1.52412000
H	5.26258100	-5.56111700	-2.06548600
C	3.45789600	-3.45442700	0.62900700
H	3.38173900	-5.26663000	1.78114700
C	3.86683700	-2.83903800	-0.56062900
H	4.83722900	-3.15501600	-2.45293200
H	2.95456600	-2.86278800	1.39022900

[TET_7R2.GF]
E = -5657.377418

C	2.82561400	-3.90647800	-0.35189000
C	3.45331500	-4.71758600	-1.30760300
H	2.85926800	-5.14130600	-2.11315400
C	4.81397100	-4.99360200	-1.23913500
C	5.60673000	-4.45832400	-0.21607800
C	4.97986500	-3.63139100	0.72822400
C	3.61703400	-3.36402500	0.66714800
H	3.15816100	-2.74140100	1.43122900
C	7.07067200	-4.68821800	-0.15421600
C	7.75372900	-4.64021800	1.06738400
H	7.19444300	-4.57544100	1.99679800
C	9.14235700	-4.62130800	1.11038100
H	9.64092400	-4.49198600	2.06725000
C	9.90455900	-4.66159400	-0.06517700
C	9.22320300	-4.83259200	-1.27810900
H	9.78566700	-4.89662600	-2.20576300
C	7.83381800	-4.84208800	-1.32074100
H	7.33851700	-4.88933300	-2.28701800
C	11.33366100	-4.26840500	-0.00957100
C	11.81263300	-3.37166300	-0.97078200
H	11.21360200	-3.11682000	-1.83996200
C	12.95793600	-2.62175400	-0.73116000
H	13.19327500	-1.82073300	-1.42647200
C	13.65653800	-2.73925700	0.47786700
C	13.29076900	-3.78119300	1.34537800
H	13.85622900	-3.94625300	2.25947900
C	12.14638300	-4.53319800	1.10513800
H	11.84059200	-5.28526500	1.82852900
C	14.39659400	-1.53562900	0.93693800
C	15.03916300	-0.65396400	0.05323000
H	15.40035800	-1.01837900	-0.90527400
C	15.07622900	0.71192400	0.31697000
H	15.47817000	1.37422900	-0.44426400
C	14.45101100	1.24311000	1.46001300
C	14.09846500	0.32634600	2.46162300
H	13.65555400	0.67163500	3.38932100
C	14.07475700	-1.03858200	2.20665600

C	6.74940300	1.13847000	0.01161500	H	13.61281000	-1.69490400	2.93882700
C	4.28854500	1.48943200	-0.00547000	C	13.75638500	2.56220200	1.43155200
C	4.04485000	2.37681000	-1.06042100	C	13.02100300	3.02128900	2.53671000
H	4.75735000	2.43801500	-1.88022700	H	13.26403100	2.67847000	3.53759400
C	2.86761000	3.11985100	-1.10920900	C	11.87137800	3.78256600	2.36309500
C	1.91646500	3.02011700	-0.08594300	H	11.24513600	3.97772600	3.22899200
C	2.21995000	2.21884000	1.02233500	C	11.43604800	4.15818100	1.08312400
C	3.37923100	1.45174100	1.05443700	C	12.33057500	3.94573800	0.02510800
H	3.55648800	0.77413900	1.88676900	H	12.08916500	4.29979600	-0.97241100
C	0.52480500	3.51603100	-0.24213800	C	13.45668700	3.15048800	0.19281400
C	-0.22540600	4.05649000	0.81103600	H	14.01984400	2.87748500	-0.69399900
H	0.27939100	4.37372900	1.72081700	C	10.00923300	4.48153400	0.82767600
C	-1.61582700	4.10785100	0.74717100	C	9.44564200	4.10413300	-0.40035700
H	-2.16404800	4.47205400	1.61195200	C	8.07205800	4.11391200	-0.59580000
C	-2.30987400	3.59531500	-0.36140800	H	7.66934600	3.74250600	-1.53519600
C	-1.54140000	3.22695700	-1.47674900	C	7.20114800	4.47542900	0.43701700
H	-2.02357100	2.81685400	-2.35754100	C	7.76028100	4.92374900	1.64132300
C	-0.15515700	3.19617800	-1.42205200	H	7.10557100	5.25625300	2.44330300
H	0.39720800	2.78025400	-2.26116000	C	9.13920300	4.93859500	1.82891700
C	-3.70667600	3.08639300	-0.27482300	H	10.07426500	3.70655500	-1.18939000
C	-4.36978800	2.58816000	-1.40799400	H	9.53803300	5.28457100	2.77870100
H	-4.10863100	2.94830600	-2.39872600	H	5.26062900	-5.64834100	-1.98243900
C	-5.22006500	1.49469500	-1.30688700	H	5.56816600	-3.17329100	1.51835200
H	-5.55181200	1.02443700	-2.22730500	C	5.74224600	4.28161900	0.26289500
C	-5.46838800	0.88102700	-0.06648900	C	5.10821000	4.63723200	-0.93224500
C	-5.12376600	1.63636600	1.06695600	C	4.98316700	3.64496000	1.25436700
H	-5.39234300	1.29116100	2.06073400	C	3.75140700	4.39232500	-1.11806000
C	-4.23784300	2.70303500	0.96556400	H	5.67951900	5.13023600	-1.71480800
H	-3.83303200	3.11668100	1.88486600	C	3.63029200	3.38192900	1.06153000
C	-5.54789600	-0.60840900	0.00816800	H	5.46750500	3.33022900	2.17584100
C	-5.57474000	-1.40003500	-1.15611000	C	2.98943000	3.77022100	-0.12212400
H	-6.05078400	-1.03181900	-2.06029700	H	3.26686200	4.69519800	-2.04279000
C	-4.83140900	-2.57137500	-1.23766900	H	3.05412700	2.89585200	1.84551100
H	-4.75976000	-3.07449500	-2.19831700	C	-2.82563800	3.90656700	-0.35193100
C	-4.03733400	-2.99474100	-0.15724900	C	-3.45340800	4.71759200	-1.30766600
C	-4.32758600	-2.41160100	1.08370900	H	-2.85941600	5.14127200	-2.11327900
H	-3.80336000	-2.73747800	1.97649100	C	-4.81406800	4.99358000	-1.23913800
C	-5.07176400	-1.24256400	1.16569100	C	-5.60676400	4.45836800	-0.21599200
H	-5.08912200	-0.73034300	2.12163000	C	-4.97981500	3.63153300	0.72834500
C	-2.68719400	-3.59265500	-0.35324300	C	-3.61698500	3.36418000	0.66719700
C	-1.88312500	-3.98176000	0.73068900	H	-3.15805300	2.74161500	1.43129100
H	-2.33922700	-4.24890500	1.68002700	C	-7.07071300	4.68823000	-0.15409700
C	-0.49734100	-3.87954600	0.66525400	C	-7.75376800	4.64018800	1.06750400
H	0.07852500	-4.05098800	1.57116300	H	-7.19448700	4.57539600	1.99691900
C	0.14493500	-3.41834400	-0.49404800	C	-9.14239700	4.62125400	1.11051000
C	-0.64441100	-3.29100200	-1.64443900	H	-9.64095200	4.49189200	2.06737900
H	-0.18141700	-3.02903100	-2.59177700	C	-9.90461000	4.66156400	-0.06503900
C	-2.02958200	-3.36385800	-1.57267700	C	-9.22326100	4.83262800	-1.27796700
H	-2.60236000	-3.09654400	-2.45511100	H	-9.78572700	4.89669200	-2.20561700
C	1.52725100	-2.87630500	-0.44905400	C	-7.83387800	4.84213900	-1.32060600
C	1.85833400	-1.77758200	-1.25850400	H	-7.33859700	4.88942100	-2.28689100
C	3.07291300	-1.11744800	-1.12755500	C	-11.33370300	4.26833400	-0.00944900
H	3.28667900	-0.26480900	-1.76594900	C	-11.81264500	3.37160600	-0.97068800
C	4.01334900	-1.51861700	-0.17041000	H	-11.21359600	3.11679500	-1.83986400
C	3.69714200	-2.62992900	0.62262700	C	-12.95793700	2.62167000	-0.73110300
H	4.40690700	-2.96787600	1.37423800	H	-13.19325400	1.82066500	-1.42644200
C	2.48183600	-3.29801600	0.48447400	C	-13.65656400	2.73913000	0.47791500
H	1.13164500	-1.39327800	-1.96821700	C	-13.29081900	3.78104200	1.34546200
H	2.27329800	-4.15242700	1.12341000	H	-13.85629400	3.94606700	2.25956000
H	2.66697700	3.75838900	-1.96643100	C	-12.14644300	4.53307600	1.10525900

H	1.49368000	2.12369700	1.82544100
H	6.38881400	-2.69694900	-0.04433900
H	8.63738100	-1.68064100	0.07133600
H	8.87391400	0.80140800	0.08003800
H	6.84104800	2.22173700	0.03583000
C	-1.51129100	-0.11353500	0.18462100
H	-0.79566700	0.08785400	-0.62254200
H	-1.72373700	-1.18973700	0.18633300
H	-2.44664200	0.41540300	-0.04745500
C	-0.94292300	0.31783700	1.53690700
H	-0.71234300	1.39196600	1.47729400
C	-1.96857100	0.10871800	2.65097400
H	-1.58560500	0.45379800	3.61872300
H	-2.90059400	0.64302300	2.43647700
H	-2.20677100	-0.95960100	2.74455900
C	0.34052800	-0.45013000	1.85770300
H	1.08866300	-0.34576800	1.06427300
H	0.78890300	-0.10552500	2.79883900
H	0.11883200	-1.51972400	1.96573000

[6CPP_1R. Isopropanol]
E = -1810.959702

C	5.38815300	-0.70241800	-0.04073100
C	6.69099200	1.33898200	-0.15017100
C	7.82142600	-0.77601700	0.04971800
C	5.44318100	0.70701300	-0.12700400
C	7.87467000	0.61082900	-0.06516800
C	6.58743800	-1.41920200	0.05650100
C	4.10833900	-1.46337300	-0.11460000
C	3.81379800	-2.49024700	0.79144100
H	4.51940400	-2.71501600	1.58818900
C	2.62168100	-3.20978100	0.70742900
C	1.68596200	-2.92866400	-0.29475700
C	1.99624900	-1.91470900	-1.21491500
C	3.18113600	-1.19821200	-1.12910400
H	3.38293900	-0.40822000	-1.84723900
C	0.32303600	-3.51393200	-0.34632600
C	-0.33232800	-3.70367500	-1.57345900
H	0.24720400	-3.71225800	-2.49322700
C	-1.71578400	-3.77941400	-1.64159200
H	-2.18339000	-3.84598000	-2.61924200
C	-2.50489200	-3.63673100	-0.48618200
C	-1.83405300	-3.72303000	0.74194300
H	-2.39614600	-3.67875900	1.66959600
C	-0.44430500	-3.66173700	0.81337600
H	0.02815900	-3.57623400	1.78954000
C	-3.85332300	-3.00332200	-0.54710800
C	-4.61948900	-2.74782600	0.60504400
H	-4.50657900	-3.37178800	1.48783900
C	-5.41076900	-1.60746800	0.70012200
H	-5.87615800	-1.37362500	1.65416000
C	-5.45648600	-0.67545800	-0.35194800
C	-4.97246000	-1.11695700	-1.59191100
H	-5.02185800	-0.46300200	-2.45681200
C	-4.18644700	-2.25730000	-1.68674600
H	-3.67951900	-2.43647700	-2.62853000
C	-5.45360500	0.79673300	-0.11610800
C	-4.86539100	1.23191300	1.07744000
H	-4.81207600	0.57043100	1.93611500
H	-11.84067500	5.28512600	1.82867700
C	-14.39663100	1.53548800	0.93693200
C	-15.03917600	0.65384600	0.05318300
H	-15.40033700	1.01828400	-0.90532500
C	-15.07625800	-0.71204800	0.31689000
H	-15.47817200	-1.37433300	-0.44437600
C	-14.45108400	-1.24326500	1.45994300
C	-14.09857000	-0.32652400	2.46158500
H	-13.65569700	-0.67183500	3.38929300
C	-14.07484400	1.03840900	2.20665000
H	-13.61292100	1.69471100	2.93885300
C	-13.75646000	-2.56235700	1.43147100
C	-13.02108800	-3.02145000	2.53663300
H	-13.26412800	-2.67864000	3.53751800
C	-11.87145200	-3.78271100	2.36302700
H	-11.24521500	-3.97786900	3.22892700
C	-11.43609800	-4.15830000	1.08305600
C	-12.33061600	-3.94586100	0.02503200
H	-12.08918200	-4.29990100	-0.97248800
C	-13.45674200	-3.15063000	0.19273300
H	-14.01989300	-2.87762900	-0.69408500
C	-10.00926800	-4.48159800	0.82762700
C	-9.44566200	-4.10409400	-0.40036600
C	-8.07207200	-4.11380500	-0.59577300
H	-7.66934300	-3.74232300	-1.53513100
C	-7.20117800	-4.47536700	0.43703900
C	-7.76032300	-4.92378600	1.64130300
H	-7.10562000	-5.25632000	2.44327400
C	-9.13924900	-4.93869100	1.82886200
H	-10.07428500	-3.70649200	-1.18938700
H	-9.53809400	-5.28474100	2.77861200
H	-5.26077100	5.64825700	-1.98246800
H	-5.56803500	3.17350200	1.51857100
C	0.59464300	-4.88559000	-0.48656900
C	1.34857000	-3.75101900	-0.37205900
C	0.67741900	-2.46717700	-0.26105300
C	-0.77623600	-2.43459900	-0.21888100
C	-1.51175800	-3.68288800	-0.26858700
C	-0.82668300	-4.85278400	-0.43444200
C	1.37461000	-1.26406000	-0.24714600
C	-1.42600800	-1.20556100	-0.19784600
C	-0.71814200	0.01556200	-0.19966200
C	0.71810600	-0.01543600	-0.19970600
C	1.42597900	1.20568200	-0.19795300
H	2.51377800	1.17663600	-0.20886600
C	0.77621300	2.43472400	-0.21898200
C	-0.67744300	2.46729700	-0.26109900
C	-1.37464300	1.26418800	-0.24709700
H	2.45970900	-1.26931600	-0.30825200
H	1.09212400	-5.84894600	-0.55800000
H	-1.37618100	-5.78970100	-0.47013100
H	-2.51380900	-1.17652200	-0.20870800
C	-0.59467600	4.88569800	-0.48674700
C	0.82665200	4.85290100	-0.43464000
C	1.51173200	3.68301400	-0.26873900
C	-1.34859400	3.75113000	-0.37215100
H	-1.09216800	5.84904500	-0.55822300
H	1.37614500	5.78981900	-0.47037100
H	-2.45974800	1.26945900	-0.30812300
C	-5.74227600	-4.28152100	0.26295000

C	-4.04925900	2.35745900	1.09813800
H	-3.41871300	2.49460200	1.97025400
C	-3.79840500	3.07603300	-0.07509300
C	-4.65631200	2.83739500	-1.16523100
H	-4.58645700	3.45443400	-2.05814700
C	-5.48013300	1.71828500	-1.18143500
H	-6.03047800	1.48496800	-2.08963400
C	-2.42999500	3.63872800	-0.23155700
C	-1.74133800	3.36620400	-1.42289400
H	-2.29512500	3.03648300	-2.29748400
C	-0.35098000	3.31431000	-1.45516700
H	0.14425800	2.96325500	-2.35731300
C	0.39888700	3.52492400	-0.29310600
C	-0.28006500	3.98977300	0.84337700
H	0.28337700	4.23289800	1.74106200
C	-1.66798100	4.05737800	0.87083700
H	-2.16659000	4.34310100	1.79327800
C	1.78922500	3.00797500	-0.21095800
C	2.12783100	2.21375800	0.89390700
C	3.30788100	1.48364200	0.92402300
H	3.52018300	0.83046000	1.76751200
C	4.21061600	1.54603400	-0.14214200
C	3.91797000	2.40257500	-1.20950400
H	4.61864300	2.47782700	-2.03837100
C	2.71829200	3.11312700	-1.25219200
H	1.41745900	2.12075900	1.70924200
H	2.49404100	3.74060000	-2.11162800
H	2.41678100	-3.99358800	1.43312300
H	1.27193700	-1.65120400	-1.98092700
H	6.72291000	2.42439800	-0.20729700
H	8.83085600	1.12577900	-0.07887300
H	8.73622400	-1.35757500	0.11764800
H	6.54428600	-2.50464900	0.10306200
C	-0.02355600	-0.54709300	2.47002600
C	-1.46495800	-0.46597300	1.98081900
H	-1.94144700	-1.45230100	2.06721300
H	0.03916600	-1.00184700	3.46718800
H	0.60072400	-1.13316100	1.78589200
H	0.39173900	0.46336700	2.52814800
C	-1.55140300	-0.01404200	0.53420600
H	-2.59252500	0.01877700	0.19539800
H	-1.11663700	0.98844500	0.41977800
H	-0.99939900	-0.71488500	-0.10478300
O	-2.20806200	0.46684500	2.77403200
H	-2.10097800	0.22629100	3.70557000

[6CPP_1R. GB]
E = -2366.697429

C	-5.49436800	-0.59808000	-0.67591800
C	-6.79239800	1.39914500	-0.22824400
C	-7.92529200	-0.69060900	-0.61139400
C	-5.54685400	0.78928500	-0.41395000
C	-7.97548600	0.67094700	-0.32230700
C	-6.69224000	-1.31389800	-0.78207900
C	-4.20166000	-1.33162800	-0.78897400
C	-3.90972400	-2.13904700	-1.89383800
H	-4.64369100	-2.23754300	-2.69060800
C	-2.67696700	-2.78219200	-2.00718100
C	-1.70851600	-2.65016400	-1.00668000
C	-5.10821200	-4.63711700	-0.93217900
C	-4.98323000	-3.64485500	1.25444100
C	-3.75140700	-4.39219400	-1.11796100
H	-5.67949800	-5.13012200	-1.71475700
C	-3.63035300	-3.38180700	1.06163700
H	-5.46759400	-3.33013500	2.17590500
C	-2.98946000	-3.77009200	-0.12200200
H	-3.26683400	-4.69505900	-2.04267900
H	-3.05421100	-2.89572700	1.84563200
P	10.58989000	0.37945700	-1.53633700
O	10.94847700	-0.73721100	-2.43136000
O	9.03306000	0.69856900	-1.41196800
F	11.11358200	1.78362900	-2.07209700
C	11.25816800	0.35185800	0.12507200
H	10.96673800	1.25769200	0.66815700
H	10.88020500	-0.53651700	0.64185700
H	12.34975000	0.29029700	0.07448900
C	7.38324900	-0.77294000	0.60336800
C	6.03543600	-0.08418600	0.36330000
C	6.17729900	1.00534000	-0.70208500
C	6.73322600	0.43790900	-2.01056400
C	8.04576600	-0.30587800	-1.79701700
C	7.92495400	-1.36048300	-0.70135200
H	5.21612000	1.50186700	-0.88715600
H	5.30184900	-0.83502200	0.03170000
H	5.65246200	0.35037300	1.29518600
H	8.09663400	-0.03922500	1.00476200
H	7.29351600	-1.57088100	1.35036600
H	6.01757300	-0.27599100	-2.44121100
H	6.88468300	1.23243600	-2.75014300
H	8.39203800	-0.76300500	-2.72907800
H	7.23239500	-2.13084900	-1.06569300
H	8.89085000	-1.86487200	-0.55116500
H	6.86228700	1.77499000	-0.32704600
P	-10.58982800	-0.37949700	-1.53627600
O	-10.94832300	0.73721600	-2.43128100
O	-9.03300600	-0.69849400	-1.41158700
F	-11.11339400	-1.78363600	-2.07226600
C	-11.25830600	-0.35207400	0.12504600
H	-10.96701700	-1.25800500	0.66804300
H	-10.88028400	0.53618900	0.64198100
H	-12.34987400	-0.29038300	0.07436500
C	-7.38284200	0.77344500	0.60297100
C	-6.03510900	0.08451400	0.36297700
C	-6.17718300	-1.00534900	-0.70203900
C	-6.73322300	-0.43829800	-2.01063700
C	-8.04566100	0.30569500	-1.79717500
C	-7.92462200	1.36065100	-0.70187000
H	-5.21606500	-1.50201000	-0.88707100
H	-5.30150500	0.83519600	0.03105800
H	-5.65205800	-0.34977300	1.29496000
H	-8.09625800	0.03990500	1.00462700
H	-7.29299000	1.57158700	1.34973500
H	-6.01755400	0.27539100	-2.44161000
H	-6.88484500	-1.23305300	-2.74993800
H	-8.39201900	0.76253200	-2.72934400
H	-7.23203100	2.13084700	-1.06651700
H	-8.89046900	1.86514700	-0.55172800
H	-6.86218500	-1.77483000	-0.32666700

C	-2.02131200	-1.88027600	0.12401500
C	-3.24419300	-1.23055900	0.22712200
H	-3.45398800	-0.60449500	1.09111600
C	-0.31557300	-3.14404700	-1.15394400
C	0.39015900	-3.69569100	-0.07484400
H	-0.14799000	-3.97559700	0.82725800
C	1.77593500	-3.80766800	-0.10432900
H	2.29053600	-4.17796500	0.77860900
C	2.51550100	-3.33218000	-1.20074500
C	1.79054600	-2.95300600	-2.34211100
H	2.31726300	-2.57523900	-3.21321400
C	0.40180700	-2.86972700	-2.32308900
H	-0.11588000	-2.45381000	-3.18418700
C	3.90253900	-2.80844200	-1.04877800
C	4.66622300	-2.37067400	-2.14660100
H	4.53340400	-2.83143000	-3.12234000
C	5.45942000	-1.23403500	-2.04555800
H	5.91906900	-0.83624300	-2.94678700
C	5.49624400	-0.49422700	-0.84773900
C	5.05100500	-1.15659300	0.30456800
H	5.10387400	-0.66809400	1.27252400
C	4.26743600	-2.29992900	0.20419800
H	3.76385300	-2.64255600	1.10199600
C	5.40992900	0.99516100	-0.84225400
C	4.81331200	1.59066700	-1.96351200
H	4.83249000	1.08067200	-2.92135500
C	3.96584600	2.68281900	-1.82739000
H	3.37298900	2.97662600	-2.68798200
C	3.68267500	3.22056300	-0.56482400
C	4.53134700	2.83235300	0.48786200
H	4.43306100	3.30008700	1.46407100
C	5.38480600	1.74204100	0.35051000
H	5.92443500	1.38990300	1.22601100
C	2.31734100	3.76786500	-0.33442100
C	1.66205100	3.42217300	0.85507900
H	2.21818800	3.03590500	1.70368100
C	0.27336100	3.35029000	0.91648500
H	-0.17680100	2.92664900	1.81053900
C	-0.50542200	3.61492000	-0.21413000
C	0.13875800	4.16605000	-1.33361400
H	-0.45028400	4.46508000	-2.19745400
C	1.52432400	4.25361200	-1.38842000
H	1.99301800	4.61934400	-2.29846500
C	-1.88941100	3.08216400	-0.29349600
C	-2.25870200	2.35612800	-1.43619800
C	-3.44517300	1.63631900	-1.47677800
H	-3.68869200	1.04337600	-2.35489900
C	-4.31372400	1.62574600	-0.38031200
C	-3.98133900	2.40156500	0.73595800
H	-4.64962200	2.41466300	1.59427000
C	-2.78225000	3.11174900	0.78404000
H	-1.57292700	2.30416800	-2.27791000
H	-2.52701500	3.67907100	1.67584000
H	-2.46130400	-3.38357400	-2.88734500
H	-1.26708400	-1.74100900	0.89462400
H	-6.82537300	2.46903400	-0.03667600
H	-8.93114600	1.16738400	-0.18151000
H	-8.84117400	-1.26909200	-0.69027500
H	-6.64624200	-2.38382100	-0.97085400
P	1.50211500	-0.10682900	2.11446300

Isobutane
E = -158.363519

C	0.00000000	1.45268100	-0.09644400
H	0.88655900	1.98943700	0.25907600
H	0.00000000	1.49474200	-1.19320800
H	-0.88655900	1.98943700	0.25907600
C	0.00000000	0.00000000	0.38024600
H	0.00000000	0.00000000	1.47968800
C	-1.25805800	-0.72634000	-0.09644400
H	-1.27962300	-1.76250100	0.25907600
H	-2.16618200	-0.22693600	0.25907600
H	-1.29448400	-0.74737100	-1.19320800
C	1.25805800	-0.72634000	-0.09644400
H	2.16618200	-0.22693600	0.25907600
H	1.27962300	-1.76250100	0.25907600
H	1.29448400	-0.74737100	-1.19320800

Isopropanol
E = -194.261132

C	1.19132600	-0.78090800	-0.10275300
C	-0.00118200	0.04327200	0.36795600
H	0.00639200	0.09608000	1.46820600
H	2.13746600	-0.33291500	0.22376900
H	1.14677300	-1.79801200	0.30105100
H	1.19660800	-0.83690300	-1.19662000
C	-1.32187000	-0.54992600	-0.08908100
H	-2.15481400	0.07601100	0.24259800
H	-1.34564500	-0.60449600	-1.18272700
H	-1.45426700	-1.55756600	0.31711500
O	0.04710000	1.36313800	-0.16573600
H	0.88104700	1.77807400	0.09575700

[6CPP_1R. Cyclohexanol]
E = -1927.650174

C	-5.46431200	-0.49679200	-0.48512400
C	-6.43955100	1.69878700	-0.19721500
C	-7.88027700	-0.20583300	-0.50295300
C	-5.29726300	0.89441600	-0.30187100
C	-7.72004300	1.16072800	-0.28946900
C	-6.75585400	-1.02148600	-0.59655900
C	-4.30130500	-1.42946800	-0.50302400
C	-3.96309100	-2.16929400	-1.64309400
H	-4.59384100	-2.10580100	-2.52738800
C	-2.79073900	-2.92208000	-1.68322200
C	-1.94105400	-2.98289400	-0.57018800
C	-2.35842800	-2.34816100	0.60565700
C	-3.50430000	-1.56376300	0.63582000
H	-3.74016800	-1.00820600	1.54029100
C	-0.53883900	-3.46858000	-0.65417900
C	0.14778200	-3.99103900	0.45322300
H	-0.41202800	-4.29715700	1.33405300
C	1.53829000	-4.05111200	0.47564000
H	2.03840800	-4.39785300	1.37716000
C	2.29794600	-3.58402900	-0.60863700

O	1.32525800	1.11371300	2.92454300
O	0.42152400	-1.25845700	2.34556900
C	1.61934300	0.01349900	0.33371000
H	2.51700300	0.58248100	0.06444300
H	1.67259300	-0.98874400	-0.10389400
H	0.73462800	0.53858700	-0.04041500
F	2.85378500	-0.85258800	2.52731300
C	-0.13373500	-1.47395200	3.67541400
H	0.63223400	-1.20245100	4.41192900
C	-0.44563800	-2.95552300	3.76629200
H	-0.83833600	-3.19541000	4.75919800
H	-1.20277700	-3.22674900	3.02188800
H	0.45349500	-3.55326600	3.59005100
C	-1.35774700	-0.59235000	3.84580700
H	-1.08994600	0.46147200	3.73350100
H	-2.11304800	-0.85497200	3.09689400
H	-1.79196400	-0.74213000	4.83960800

[6CPP_1R. GD]
E = -2484.585669

C	-5.29378400	-0.49525500	-1.33602000
C	-6.60101200	1.48605500	-0.84615800
C	-7.72494700	-0.56883200	-1.40463700
C	-5.35127300	0.87622800	-1.00039400
C	-7.78193500	0.77479000	-1.04239400
C	-6.48857000	-1.19313000	-1.54465400
C	-4.00097300	-1.23188200	-1.42248000
C	-3.65965600	-1.98822100	-2.54936400
H	-4.35460100	-2.04454400	-3.38433300
C	-2.42665100	-2.63516300	-2.63240000
C	-1.50710500	-2.55580100	-1.58164400
C	-1.86812500	-1.83259600	-0.43534900
C	-3.09335000	-1.18354700	-0.35865500
H	-3.34522900	-0.60706600	0.52844800
C	-0.11347300	-3.05895600	-1.67936400
C	0.51901800	-3.70175800	-0.60431100
H	-0.07790300	-4.04799200	0.23654400
C	1.90431200	-3.82262000	-0.55595500
H	2.36252300	-4.26535300	0.32456000
C	2.71147200	-3.26576300	-1.56271200
C	2.06298600	-2.78880500	-2.71232900
H	2.64697000	-2.34269500	-3.51162000
C	0.67640600	-2.69694500	-2.77516300
H	0.21390800	-2.20795300	-3.62904000
C	4.08930700	-2.76860800	-1.29016900
C	4.93234600	-2.28793300	-2.30854400
H	4.86323000	-2.69960000	-3.31256300
C	5.72822400	-1.16865400	-2.09495500
H	6.25640200	-0.73294400	-2.93939400
C	5.68561800	-0.48841800	-0.86285600
C	5.14778800	-1.19674200	0.22077300
H	5.13148700	-0.75358500	1.21197600
C	4.36388500	-2.32458800	0.00896900
H	3.79113200	-2.70240900	0.84871800
C	5.62282300	0.99984900	-0.78714100
C	5.10410400	1.66150100	-1.90986000
H	5.17891900	1.20334400	-2.89095100
C	4.25807700	2.75406000	-1.76639700
H	3.72010000	3.09979700	-2.64382000
C	1.60216000	-3.23033700	-1.77410900
H	2.15131200	-2.85051000	-2.63072300
C	0.21391200	-3.17557000	-1.79725100
H	-0.28075400	-2.76706800	-2.67438400
C	3.69034900	-3.08015500	-0.46773300
C	4.52135100	-2.84156100	-1.57599900
H	4.38474300	-3.41573900	-2.48936600
C	5.40413000	-1.76812300	-1.57935100
H	5.93251900	-1.52819200	-2.49845500
C	5.46818000	-0.88990100	-0.48158400
C	4.91349600	-1.35482700	0.72015600
H	4.98714800	-0.75499200	1.62267800
C	4.03634300	-2.43368700	0.72494100
H	3.46871200	-2.63286100	1.62898200
C	5.55369200	0.58750300	-0.66888700
C	5.06786600	1.09211700	-1.88497700
H	5.07142900	0.46748400	-2.77226100
C	4.33127000	2.26699300	-1.92600400
H	3.81025800	2.49785900	-2.84862000
C	4.05058100	2.98560900	-0.75438600
C	4.84125100	2.66960200	0.36553400
H	4.77311400	3.26739100	1.27077300
C	5.58135600	1.49054800	0.40797600
H	6.05690200	1.20828600	1.34440000
C	2.71368700	3.63942000	-0.63763800
C	2.06951200	3.67047600	0.60675600
H	2.65191300	3.57364400	1.51734800
C	0.68073200	3.61096500	0.70624400
H	0.22926100	3.48047400	1.68746500
C	-0.11218000	3.51337000	-0.44147900
C	0.51826000	3.75845400	-1.67274000
H	-0.08098000	3.80879300	-2.57843800
C	1.90015300	3.83512700	-1.76837600
H	2.34624600	3.94353200	-2.75246700
C	-1.48156000	2.93984600	-0.39425100
C	-1.81877000	1.97284000	-1.35436100
C	-3.03068600	1.29720500	-1.30775100
H	-3.25728100	0.55193700	-2.06510200
C	-3.96135300	1.55943400	-0.29524500
C	-3.62673400	2.52180800	0.66660900
H	-4.32677900	2.73434400	1.47124800
C	-2.40818300	3.19906200	0.62194400
H	-1.09942400	1.71882000	-2.12853300
H	-2.17911300	3.93826800	1.38628600
H	-2.51933200	-3.44133400	-2.59934400
H	-1.73582800	-2.38080600	1.49206700
H	-6.31327300	2.77200800	-0.07685100
H	-8.58698800	1.81063400	-0.21305500
H	-8.87334700	-0.63753200	-0.58723600
H	-6.87090400	-2.09444800	-0.73091700
C	2.13135600	-0.29314200	3.33018000
C	0.70698600	-0.32150400	3.89261100
C	-0.28435200	-0.75457800	2.81613800
C	-0.21836900	0.16017600	1.60101500
C	1.20050100	0.18854400	1.02902500
C	2.20052100	0.61875600	2.10358200
H	2.83634800	0.03830000	4.10165400
H	0.64635900	-1.00170200	4.75425200
H	-0.94485000	-0.16473200	0.84789300
H	1.24701200	0.86759000	0.16859700

C	3.90153800	3.22396400	-0.49546300
C	4.68141700	2.77230000	0.58456800
H	4.52548000	3.18630200	1.57728700
C	5.53260700	1.68204300	0.44013800
H	6.01212400	1.27503000	1.32666300
C	2.52573600	3.76365100	-0.31552100
C	1.80550200	3.34831400	0.81274800
H	2.31240000	2.90455900	1.66320600
C	0.41586700	3.27913000	0.79081900
H	-0.08900000	2.80804100	1.63140200
C	-0.29895400	3.61647100	-0.36258500
C	0.40752000	4.22972300	-1.40972400
H	-0.13254700	4.58187400	-2.28563100
C	1.79437700	4.31424700	-1.38181700
H	2.31413000	4.73082400	-2.24095800
C	-1.67988100	3.10354200	-0.54773400
C	-1.98824200	2.44002400	-1.74543200
C	-3.18037200	1.74515400	-1.89447100
H	-3.37696000	1.19899600	-2.81379900
C	-4.11482400	1.69546600	-0.85425100
C	-3.83995100	2.40864000	0.31730600
H	-4.55847700	2.38833700	1.13435200
C	-2.63596700	3.09512200	0.47377600
H	-1.25259700	2.41714900	-2.54543600
H	-2.42711100	3.60953600	1.40922700
H	-2.17227200	-3.20000200	-3.52623700
H	-1.15457900	-1.73709800	0.38181800
H	-6.63726400	2.54401700	-0.59737700
H	-8.74065800	1.27125000	-0.92439600
H	-8.63892200	-1.13312600	-1.56564200
H	-6.43974200	-2.25139000	-1.78995600
P	1.50894400	-0.22823500	2.01802400
O	1.66437700	1.00247800	2.81842500
O	0.22597200	-1.11451200	2.35829700
C	1.52664800	-0.11400700	0.23318300
H	0.66163000	0.47358800	-0.09229600
H	2.44459500	0.39861900	-0.07871200
H	1.49074800	-1.11301700	-0.21382500
C	-0.27029900	-1.13734100	3.73295200
C	-1.75453900	-0.72266900	3.72917000
F	2.69687800	-1.25138800	2.32925200
C	-2.65064700	-1.83504300	3.17518700
H	-2.29174200	-2.20081000	2.20824400
H	-2.70355600	-2.68764100	3.86141900
H	-3.67140300	-1.46097800	3.03401800
C	-1.90912100	0.55208100	2.88904100
H	-1.21605700	1.33063700	3.23336700
H	-1.70914500	0.37099100	1.82684500
H	-2.93140200	0.93908100	2.97427300
C	-2.16918800	-0.40779200	5.17346100
H	-3.21951500	-0.09714000	5.19759300
H	-2.06905100	-1.27895000	5.82986500
H	-1.56768100	0.40830700	5.59079800
C	0.03615300	-2.52030500	4.28208700
H	-0.39627900	-3.29178400	3.63835100
H	1.11808200	-2.67429900	4.32376600
H	-0.36634300	-2.63999500	5.29206600
H	0.28198200	-0.37349800	4.29413300

[TET_6R1. GA]

H	3.21388300	0.65116600	1.68890000
O	-1.62648300	-0.74280900	3.29261600
H	-1.66270400	-1.21520400	4.13693200
H	0.41934000	0.67920400	4.24515600
H	2.42435500	-1.31458000	3.04794200
H	1.95770500	1.64087400	2.42612200
H	1.47042500	-0.81294100	0.65532300
H	-0.52165000	1.16871000	1.91800300
H	-0.01281500	-1.77840600	2.49633100

[6CPP_1R. Ethanol]
E = -1771.660661

C	-5.40592100	-0.65287900	-0.04304800
C	-6.55843300	1.47211400	0.11038200
C	-7.83932500	-0.56216300	0.01207400
C	-5.35675900	0.75772200	0.02585500
C	-7.79092700	0.82630900	0.10776300
C	-6.65338700	-1.28737800	-0.05991100
C	-4.17599900	-1.49535200	-0.04752100
C	-3.94234600	-2.43773900	-1.05626600
H	-4.67664600	-2.56256000	-1.84904800
C	-2.76184700	-3.18008600	-1.08442400
C	-1.79357500	-3.01544200	-0.08783100
C	-2.05712800	-2.11434000	0.95530700
C	-3.22533300	-1.36317700	0.97002500
H	-3.38694200	-0.63377900	1.75994500
C	-0.41898700	-3.57590400	-0.17482400
C	0.25590000	-4.05187900	0.95801300
H	-0.31031300	-4.28042800	1.85739000
C	1.64410500	-4.11944200	0.98876300
H	2.13975700	-4.40162400	1.91411100
C	2.40760500	-3.68303500	-0.10387600
C	1.72436700	-3.41887400	-1.30186400
H	2.28117900	-3.09186800	-2.17587700
C	0.33264400	-3.37395900	-1.33982800
H	-0.16459900	-3.03197100	-2.24485900
C	3.76168500	-3.08846100	0.06169500
C	4.62920500	-2.85393500	-1.02006300
H	4.57100700	-3.47976200	-1.90753900
C	5.44969000	-1.73132700	-1.03846700
H	6.00600900	-1.50201900	-1.94400200
C	5.41814100	-0.80479800	0.02005900
C	4.81883200	-1.23387600	1.21218400
H	4.79266000	-0.56954100	2.07124700
C	3.99601600	-2.35471200	1.23122900
H	3.36362100	-2.51150000	2.10066700
C	5.44124000	0.66707200	-0.22181500
C	4.93621200	1.10241600	-1.45642600
H	4.95503900	0.43729300	-2.31410100
C	4.17002600	2.25578300	-1.54771300
H	3.64253900	2.43351800	-2.47853600
C	3.87790500	3.02363400	-0.41025700
C	4.66738400	2.76942200	0.72564000
H	4.58501800	3.40788200	1.60193500
C	5.43509600	1.61090800	0.81926300
H	5.91555100	1.37878300	1.76679000
C	2.54022500	3.68066000	-0.33627400
C	1.86509900	3.73320600	0.89168100
H	2.42136600	3.65145400	1.82004200

E = -2875.739830

C	-3.39540600	3.88126400	-0.46651600
C	-2.77429300	4.76164500	0.42902400
H	-3.37482100	5.24818600	1.19341100
C	-1.40672600	5.00394600	0.37025400
C	-0.60544400	4.36639900	-0.58339000
C	-1.23149600	3.50391500	-1.49484200
C	-2.60084100	3.26736600	-1.44166400
H	-3.05934800	2.59629300	-2.16389600
C	0.87267800	4.48405100	-0.56823500
C	1.63118100	4.36013200	-1.73979500
H	1.13457500	4.35950600	-2.70679500
C	3.00226900	4.14315200	-1.67969400
H	3.53627100	3.91795300	-2.59887500
C	3.66937900	4.06049800	-0.44743400
C	2.93625600	4.37115700	0.70549200
H	3.41903500	4.35285600	1.67860800
C	1.56225900	4.56376800	0.64776900
H	1.00881300	4.65542900	1.57753900
C	4.93367200	3.28253800	-0.37328200
C	5.08248700	2.39297700	0.69906000
H	4.44078200	2.48726600	1.57036500
C	5.84331700	1.23964100	0.56833300
H	5.76792600	0.48284100	1.34591900
C	6.47143200	0.92596000	-0.64416500
C	6.53757100	1.93983800	-1.61326500
H	7.08849800	1.76946100	-2.53547200
C	5.78650400	3.10314200	-1.47555200
H	5.77853700	3.82479800	-2.28865800
C	6.54150100	-0.52025900	-0.98175100
C	6.72805400	-1.51963900	-0.01274000
H	7.28781800	-1.29397000	0.89239800
C	6.07512000	-2.74345800	-0.12157700
H	6.15398400	-3.45299700	0.69844500
C	5.20799600	-3.00435000	-1.19710900
C	5.27549200	-2.12522600	-2.28695300
H	4.65784400	-2.29528100	-3.16305000
C	5.93328100	-0.90654300	-2.18344100
H	5.79789900	-0.17431300	-2.97469200
C	3.98871600	-3.84652200	-1.05193100
C	3.14981400	-4.12422100	-2.14153700
H	3.53612200	-4.06829400	-3.15518900
C	1.79049700	-4.35208200	-1.95733200
H	1.14898200	-4.44925900	-2.82947000
C	1.21897600	-4.33244800	-0.67733400
C	2.09105000	-4.26564000	0.41641400
H	1.69242400	-4.32680600	1.42633300
C	3.44776900	-4.03255100	0.22967600
H	4.07626400	-3.88848200	1.10292600
C	-0.24917500	-4.24905800	-0.49253500
C	-0.77509000	-3.41858500	0.50800200
C	-2.14792000	-3.24849100	0.65313600
H	-2.52617400	-2.61178800	1.44936300
C	-3.04268500	-3.89898400	-0.20483000
C	-2.51528200	-4.72862200	-1.20399100
H	-3.19682100	-5.23841200	-1.88024300
C	-1.14346500	-4.90444300	-1.34546300
H	-0.10208900	-2.87176300	1.16317700
H	-0.76559300	-5.56653900	-2.12073600
C	0.47438900	3.67354200	0.95508200
H	-0.00135200	3.54660100	1.92465800
C	-0.28840500	3.56708800	-0.21234600
C	0.37294400	3.79331000	-1.43038800
H	-0.20266100	3.83502300	-2.35178500
C	1.75747600	3.86122600	-1.49062900
H	2.23139500	3.95333000	-2.46352900
C	-1.65557800	2.98796100	-0.18434000
C	-1.96822500	1.99991300	-1.13141800
C	-3.15405000	1.28218900	-1.06348200
H	-3.35578200	0.50574800	-1.79687100
C	-4.08068700	1.52551800	-0.04346900
C	-3.78870400	2.53443700	0.88301900
H	-4.49687100	2.74268700	1.68196900
C	-2.59389600	3.25075100	0.81989600
H	-1.24286300	1.75485500	-1.90271200
H	-2.38828600	4.01672800	1.56400000
H	-2.58480800	-3.88192800	-1.89635900
H	-1.29506100	-1.94114000	1.71014000
H	-6.51712200	2.55809100	0.14552700
H	-8.70738200	1.40626900	0.16616200
H	-8.79366600	-1.08069300	0.00467600
H	-6.68326900	-2.37361700	-0.09933700
C	2.03649800	0.65094400	2.25216600
H	2.42751300	-0.20794300	2.80835200
H	2.78061000	1.45115900	2.28138500
H	1.12347500	0.99263100	2.75136700
C	1.73730800	0.23524800	0.82465200
H	1.20778700	1.03725900	0.28978800
H	2.67114400	0.02353400	0.28301500
O	0.93039700	-0.93105300	0.90838700
H	0.80589000	-1.30972200	0.02469100

[6CPP_1R. GA]
E = -2414.982290

C	5.73769900	0.60439300	-0.30935900
C	6.80164400	-1.55260100	-0.03681100
C	8.15954700	0.43201000	-0.15847900
C	5.63239700	-0.79975800	-0.19661800
C	8.05573900	-0.94901600	-0.01255000
C	7.00509000	1.19658100	-0.30219200
C	4.53111600	1.47577800	-0.37499800
C	4.32392900	2.37435600	-1.42748700
H	5.07352400	2.45906200	-2.21115300
C	3.14640400	3.11606300	-1.51605400
C	2.15226600	2.99746300	-0.53815400
C	2.40339300	2.15798300	0.55628300
C	3.56500000	1.40221000	0.63388500
H	3.70972500	0.71411700	1.46306600
C	0.77441500	3.52356500	-0.71522600
C	0.00586400	4.00413600	0.35556500
H	0.49923600	4.26766800	1.28886200
C	-1.38297000	4.06294800	0.27143900
H	-1.95660000	4.36499200	1.14516200
C	-2.05380200	3.61953100	-0.88081800
C	-1.26913800	3.33523700	-2.00874500
H	-1.74914300	2.99112900	-2.92068100
C	0.11831400	3.29213500	-1.92944800
H	0.68593700	2.93288300	-2.78454000

H	-0.95610600	5.69230200	1.08042000
H	-0.63155500	2.97715900	-2.23213200
C	-6.63969300	-5.01284200	0.21244000
C	-7.34604000	-3.85279500	0.23258400
C	-6.67845900	-2.58816300	0.10912600
C	-5.23499600	-2.54313200	-0.02026700
C	-4.51851800	-3.80890600	-0.04137400
C	-5.21827500	-4.98079900	0.06842500
C	-7.40800200	-1.41001100	0.09204300
C	-4.61660600	-1.30192000	-0.14826200
C	-5.35299400	-0.09361300	-0.14789700
C	-6.78687700	-0.15535000	-0.03910300
C	-7.52590200	1.04065700	-0.06654600
H	-8.61005200	0.99213700	0.01963300
C	-6.91133300	2.27645700	-0.19355600
C	-5.46735200	2.35548700	-0.29086600
C	-4.73201500	1.17306000	-0.25368700
H	-8.49249800	-1.45499200	0.17569800
H	-7.14205300	-5.97069900	0.30956400
H	-8.42798300	-3.85497600	0.33975200
H	-4.67010100	-5.91940300	0.06776900
H	-3.53761700	-1.24488100	-0.26600000
C	-5.67104400	4.78445700	-0.38681700
C	-7.09445900	4.69471500	-0.29769800
C	-7.69454800	3.47928900	-0.20686400
C	-4.86618700	3.67679100	-0.39005900
H	-5.21157200	5.76546000	-0.47685700
H	-7.68595400	5.60535400	-0.30888000
H	-8.77602400	3.38992600	-0.13894700
H	-3.64664200	1.21043800	-0.29130500
P	1.96847300	0.21339200	2.09286100
O	1.88427500	-0.70523200	0.80062700
O	1.62088900	1.64505000	2.04197300
C	2.81741800	-0.56045000	-0.30568100
H	3.83900600	-0.60601200	0.09334800
H	2.64160500	-1.45074800	-0.91428900
C	2.56827600	0.72211900	-1.07286200
H	2.60229100	1.59336600	-0.41254700
H	3.34557200	0.84387100	-1.83730800
H	1.58833900	0.69879200	-1.55918100
N	3.45817700	-0.07446300	2.75126000
C	3.95007600	-1.44137200	2.86919000
H	3.68750400	-1.87819500	3.84323800
H	3.52297800	-2.05735700	2.07546200
H	5.04150400	-1.44416300	2.76167000
C	3.97659900	0.84472400	3.76097100
H	5.05935300	0.95480200	3.62667000
H	3.50274100	1.82110700	3.64745100
H	3.78505800	0.46604200	4.77408000
C	0.77953700	-0.74710200	3.06905500
N	0.08390300	-1.38194400	3.74145200

[TET_6R1. GD]
E = -2945.350072

C	2.42797500	4.08103300	-0.41293400
C	1.79689500	4.85052500	-1.40133400
H	2.40700100	5.36808900	-2.13735800
C	0.41223100	4.96479100	-1.45425700
C	-0.40154500	4.30570300	-0.52401000
C	-3.42687800	3.04693100	-0.84164700
C	-4.18362900	2.81018100	-2.00265400
H	-4.02266200	3.42133100	-2.88795500
C	-5.02754700	1.70762800	-2.08873500
H	-5.50240800	1.48467000	-3.04104400
C	-5.12252600	0.79344900	-1.02317700
C	-4.61895900	1.21602800	0.21589900
H	-4.66586000	0.54971500	1.07192500
C	-3.78549800	2.32480900	0.30370900
H	-3.24521000	2.48436400	1.23343700
C	-5.20094700	-0.68039700	-1.23472400
C	-4.62800100	-1.18014500	-2.41494400
H	-4.57807600	-0.55446300	-3.30127500
C	-3.87027500	-2.34403400	-2.39555500
H	-3.27111600	-2.57257700	-3.27150400
C	-3.66112100	-3.04360600	-1.19672600
C	-4.52763600	-2.73718600	-0.13448800
H	-4.48999000	-3.31164100	0.78647200
C	-5.28663000	-1.57274800	-0.15307500
H	-5.80605800	-1.28169300	0.75589600
C	-2.31546300	-3.64116600	-0.96438500
C	-1.74046900	-3.49626500	0.30801900
H	-2.36900100	-3.26240500	1.16188600
C	-0.36053200	-3.41449000	0.46841800
H	0.04307300	-3.16448900	1.44671300
C	0.49154700	-3.45383600	-0.63964900
C	-0.06836600	-3.82446000	-1.87226200
H	0.57965500	-3.96016400	-2.73523000
C	-1.44530500	-3.93651400	-2.02781400
H	-1.84524800	-4.15252500	-3.01498200
C	1.86407900	-2.89515400	-0.54408600
C	2.28955400	-1.98957700	-1.52847800
C	3.49537200	-1.31001500	-1.40946800
H	3.79107300	-0.59713600	-2.17547500
C	4.32926500	-1.51719200	-0.30453400
C	3.92120200	-2.44047600	0.66522500
H	4.55396800	-2.61756500	1.53222900
C	2.70442900	-3.11205800	0.55365000
H	1.64134500	-1.78296800	-2.37692700
H	2.40181600	-3.80784300	1.33255900
H	2.98732500	3.77585500	-2.36586000
H	1.63779200	2.04036300	1.31762800
H	6.71872600	-2.63427900	0.03662200
H	8.94786300	-1.55728900	0.10541000
H	9.13242400	0.91475500	-0.14727900
H	7.07612400	2.27906800	-0.37854200
P	-1.64313400	-0.37169600	2.36420300
O	-0.50319800	-0.30779600	1.26723500
O	-2.96087500	-0.96936300	2.06545400
C	-0.68372400	0.45489100	0.03600100
H	-1.07794700	1.44042900	0.30453100
H	0.33108000	0.57461000	-0.35031300
C	-1.58370900	-0.26888800	-0.94279600
H	-2.54082700	-0.52708000	-0.47885700
H	-1.78757200	0.39085500	-1.79551700
H	-1.11209700	-1.18756000	-1.30673500
N	-1.74963600	1.14749400	3.01195200
C	-0.53838700	1.82954100	3.45505300
H	-0.32826600	1.62083400	4.51336400
H	0.31454400	1.50354800	2.85751400

C	0.22881000	3.53129300	0.46203000
C	1.61635600	3.42455200	0.51760300
H	2.07978500	2.82974500	1.30227100
C	-1.88371500	4.31861900	-0.62388600
C	-2.69526200	4.17228300	0.50776700
H	-2.25668800	4.22327000	1.50120700
C	-4.04272700	3.84898100	0.38451100
H	-4.59169700	3.60367800	1.28764100
C	-4.63696800	3.65780100	-0.87192400
C	-3.87442900	4.02420200	-1.99364600
H	-4.30650000	3.96902100	-2.98874000
C	-2.52433900	4.33256200	-1.87248200
H	-1.94105900	4.48944600	-2.77611600
C	-5.78858100	2.71432500	-0.98368400
C	-5.82075100	1.85552300	-2.09200900
H	-5.28115300	2.11285300	-2.99728100
C	-6.31318100	0.56172300	-1.98186100
H	-6.13391300	-0.12777700	-2.80166900
C	-6.78549600	0.07142400	-0.75543700
C	-7.04135000	1.02358300	0.24627400
H	-7.50060900	0.71420500	1.18223300
C	-6.55914200	2.32673700	0.12869200
H	-6.66490900	2.99762200	0.97723600
C	-6.51261800	-1.36699100	-0.47690000
C	-6.50013600	-2.33572600	-1.49519600
H	-7.10645300	-2.19073800	-2.38646300
C	-5.62004800	-3.41039200	-1.44236100
H	-5.56891600	-4.09335200	-2.28687900
C	-4.72010400	-3.55352500	-0.37131100
C	-4.92030800	-2.73600900	0.74767700
H	-4.26074400	-2.79584500	1.60904600
C	-5.80896700	-1.66571300	0.69767200
H	-5.79098600	-0.95957300	1.52407100
C	-3.41883200	-4.25971700	-0.51660800
C	-2.66852500	-4.69743100	0.58399800
H	-3.13896600	-4.77869400	1.56050800
C	-1.29840000	-4.91824600	0.47301600
H	-0.72939900	-5.13687700	1.37331700
C	-0.62747000	-4.72745400	-0.74375700
C	-1.41139600	-4.47432300	-1.87688100
H	-0.93903100	-4.38268500	-2.85135800
C	-2.77516300	-4.23598900	-1.76361300
H	-3.32646100	-3.92439600	-2.64634400
C	0.84626400	-4.53765300	-0.77827700
C	1.38564800	-3.55408700	-1.62374600
C	2.71783100	-3.16669200	-1.53651900
H	3.09849200	-2.40532900	-2.21251200
C	3.57070400	-3.74076700	-0.58634100
C	3.04649700	-4.75888400	0.22509500
H	3.68846500	-5.22315700	0.96936300
C	1.71622700	-5.15633800	0.12739900
H	0.73853300	-3.03893500	-2.32850700
H	1.34433300	-5.93491800	0.78863000
H	-0.04040100	5.58340600	-2.22514500
H	-0.37368700	2.97271700	1.17613900
C	7.32829300	-4.12934700	-0.29604300
C	7.79107500	-2.85579800	-0.20702100
C	6.88263100	-1.74529500	-0.23719600
C	5.45683400	-1.97470900	-0.36857800
C	5.00322900	-3.35839000	-0.46181600
H	-0.66553100	2.90941500	3.32361700
C	-2.94835600	1.50899800	3.76600900
H	-3.12036500	2.58596500	3.66020500
H	-3.81021500	0.97054400	3.36940600
H	-2.83496500	1.27553700	4.83351100
C	-0.71594600	-1.37258100	3.55761800
N	-0.15910500	-1.96398100	4.38168600

[6CPP_1R. GF]
E = -2483.391161

C	5.38994500	1.27659800	-0.51529400
C	6.82084300	-0.63399100	-0.93870000
C	7.81133500	1.47951200	-0.35273700
C	5.53658900	-0.08251000	-0.87379100
C	7.95307000	0.13330200	-0.68022700
C	6.53896800	2.03791000	-0.27041100
C	4.05543100	1.91994000	-0.34229500
C	3.74700300	3.12599800	-0.98202700
H	4.49072500	3.59302300	-1.62395000
C	2.49090100	3.71606300	-0.84068900
C	1.51141800	3.12405000	-0.03771200
C	1.83245400	1.93196900	0.62951600
C	3.07981900	1.33961800	0.47745900
H	3.29357100	0.39615700	0.97617300
C	0.10813300	3.60882000	0.02572100
C	-0.59875800	3.66580600	1.23539100
H	-0.05826000	3.57575900	2.17499400
C	-1.98716900	3.74816100	1.25334700
H	-2.49665300	3.71547200	2.21190000
C	-2.72840700	3.72616800	0.05979800
C	-2.00585200	3.85773300	-1.13758700
H	-2.53054300	3.84777700	-2.08788500
C	-0.61504700	3.81196500	-1.15478500
H	-0.09557000	3.79271400	-2.10997900
C	-4.10033200	3.14445400	-0.00069000
C	-4.83246000	3.07906700	-1.20036600
H	-4.71120800	3.84757800	-1.95934600
C	-5.57564300	1.94707000	-1.51143700
H	-6.01049000	1.86791100	-2.50409200
C	-5.58299100	0.83656700	-0.64472800
C	-5.19210400	1.08717900	0.67908300
H	-5.23281200	0.29730900	1.42423200
C	-4.47160400	2.23345600	0.99723400
H	-4.01263400	2.28074200	1.97732300
C	-5.42334000	-0.55325800	-1.16525100
C	-4.89389400	-0.69692100	-2.45902100
H	-5.02088700	0.09288800	-3.19199000
C	-3.99229600	-1.70999900	-2.75217200
H	-3.46675400	-1.66180800	-3.70062700
C	-3.58638200	-2.62126000	-1.76570000
C	-4.35806000	-2.66273900	-0.59369300
H	-4.15104700	-3.40220900	0.17406200
C	-5.26564800	-1.64989800	-0.30155700
H	-5.72118700	-1.64431800	0.68431700
C	-2.19360500	-3.14405900	-1.82217300
C	-1.47402600	-3.27031500	-0.62380900
H	-2.00047500	-3.26584900	0.32704800
C	-0.08472100	-3.18799000	-0.61581100
H	0.43893200	-3.14780500	0.33661800

C	5.92634800	-4.37009000	-0.42395700
C	7.36307200	-0.45044900	-0.12689500
C	4.60825400	-0.86815100	-0.37556400
C	5.08996500	0.46196000	-0.28630100
C	6.50838000	0.66553700	-0.15118300
C	7.00666200	1.97624100	-0.05925700
H	8.08093800	2.12610100	0.03343900
C	6.17159800	3.08063000	-0.10176000
C	4.73899700	2.89837400	-0.23920600
C	4.24146000	1.59858500	-0.33444200
H	8.43437100	-0.28902600	-0.02004100
H	8.01243500	-4.97234000	-0.27947900
H	8.85414500	-2.64852700	-0.11234000
H	5.58086100	-5.39610300	-0.51926700
H	3.53700500	-1.01753300	-0.44283200
C	4.51253600	5.32875200	-0.25257000
C	5.92485300	5.49397300	-0.11864500
C	6.72760800	4.40217300	-0.03662400
C	3.91346100	4.09743500	-0.30376500
H	3.88427000	6.21501700	-0.28271600
H	6.34107700	6.49578600	-0.07097400
H	7.80470800	4.50205700	0.07281300
H	3.17345100	1.44324200	-0.47106000
P	-2.94643000	0.46995700	1.98117600
O	-3.42994300	-0.70774300	2.72831400
O	-1.37747500	0.74903000	2.04531800
C	-3.42667400	0.61445300	0.26155800
H	-3.33782100	-0.36398100	-0.21593400
H	-4.47048700	0.92678100	0.21563100
H	-2.80140400	1.35368600	-0.24916400
C	-0.45112000	-0.20943300	2.63176900
C	0.26141200	-0.96549600	1.49614800
F	-3.48895000	1.83344900	2.59958200
C	1.22240600	-0.05546400	0.73225900
H	0.72221500	0.86811700	0.42166400
H	2.11096500	0.20216200	1.32201300
H	1.55495500	-0.57163400	-0.17613200
C	-0.78069400	-1.48605600	0.50008700
H	-1.62362600	-1.98220200	0.99939400
H	-1.15882800	-0.66974400	-0.12118900
H	-0.31739800	-2.21232700	-0.17029000
C	1.02762200	-2.15294600	2.08807100
H	1.55863800	-2.68167100	1.28942100
H	1.77387100	-1.83809700	2.82562500
H	0.34494200	-2.86574400	2.56855200
C	0.44306700	0.59320900	3.56010800
H	0.93526400	1.40495300	3.01524400
H	-0.15408300	1.03378900	4.36292600
H	1.21191500	-0.04116000	4.00949800
H	-1.04462300	-0.93059800	3.20736300

C	0.63180500	-2.96650000	-1.79668000
C	-0.07048600	-3.05026100	-3.00697700
H	0.46862500	-2.96529500	-3.94768800
C	-1.45666000	-3.15574800	-3.01876900
H	-1.97358600	-3.15485700	-3.97452200
C	2.00390900	-2.40753800	-1.71172400
C	2.29307100	-1.24155500	-2.43701400
C	3.44869500	-0.51434600	-2.18929600
H	3.62515100	0.41746400	-2.72081900
C	4.36214900	-0.93216400	-1.21503000
C	4.12104000	-2.14159700	-0.55457400
H	4.82786800	-2.49072200	0.19532900
C	2.95410300	-2.86584500	-0.79311100
H	1.56398100	-0.86232100	-3.14851300
H	2.76043500	-3.77754400	-0.23158800
H	2.26789400	4.64271500	-1.36439800
H	1.07276200	1.43244600	1.22778700
H	6.92552200	-1.67712800	-1.22756500
H	8.93971700	-0.31587000	-0.74618600
H	8.68561100	2.09080600	-0.14876900
H	6.42261800	3.07902700	0.02111500
P	-1.84803100	-0.38043000	1.63039200
O	-2.74071500	-1.52279400	1.91018500
O	-0.31278500	-0.52020200	2.04021100
F	-2.26227700	0.88165100	2.52244700
C	-1.76144000	0.23804100	-0.04641500
H	-1.31040700	1.23519500	-0.06329100
H	-1.14815800	-0.45561300	-0.63175700
H	-2.76977200	0.27474400	-0.47147900
C	0.12054500	-3.17940700	3.25206300
C	1.64708100	-3.16197200	3.09999600
C	2.16250500	-1.83114700	2.53862000
C	1.64873500	-0.65020300	3.36474800
C	0.12640700	-0.66396200	3.42247900
C	-0.38596300	-1.96628900	4.03429100
H	3.25930200	-1.82659500	2.52016400
H	2.10395700	-3.32977600	4.08644500
H	1.97124100	-3.99310300	2.46090100
H	-0.36452400	-3.18074400	2.26844600
H	-0.19752200	-4.09990700	3.75380900
H	2.02391600	-0.71495300	4.39500400
H	1.99821700	0.30332100	2.95448100
H	-0.25215100	0.20435800	3.97461200
H	-0.01511500	-2.00052000	5.06819500
H	-1.47918900	-1.96761100	4.07694500
H	1.83346400	-1.70515000	1.49849800

TET_6R1
E = -2077.443502

C	-3.01578700	2.48894500	2.66158200
C	-3.10368900	3.74601700	2.04709400
H	-3.01997700	4.64073900	2.65882100
C	-3.28238800	3.86935800	0.67360900
C	-3.36379800	2.73714600	-0.14583500
C	-3.28906900	1.47970300	0.47218600
C	-3.12717000	1.35592400	1.84721800
H	-3.09037300	0.36614600	2.29525900
C	-3.40273200	2.82935600	-1.62656500
C	-3.96891400	1.80838200	-2.39954700

	H	-4.55745000	1.03121900	-1.91969100
	C	-3.71055000	1.71586600	-3.76116700
	H	-4.05483000	0.83699400	-4.29849100
	C	-2.89578000	2.65342900	-4.41258500
	C	-2.49837200	3.77877000	-3.67565500
	H	-1.91765300	4.56216800	-4.15547400
	C	-2.73408700	3.85717300	-2.30773100
	H	-2.30753600	4.68612500	-1.74915700
	C	-2.21056700	2.24602500	-5.66787800
	C	-0.85639600	2.57324200	-5.81257300
	H	-0.42039500	3.35888600	-5.20316800
	C	0.00000000	1.75425900	-6.53674900
	H	1.06929400	1.92679000	-6.45711900
	C	-0.46903600	0.57751500	-7.13600500
	C	-1.86137300	0.42390900	-7.23811400
	H	-2.27385200	-0.42158400	-7.78386200
	C	-2.71858700	1.24715100	-6.51603000
	H	-3.78478500	1.03476300	-6.52461000
	C	0.46903600	-0.57751500	-7.13600500
	C	1.86137300	-0.42390900	-7.23811400
	H	2.27385200	0.42158400	-7.78386200
	C	2.71858700	-1.24715100	-6.51603000
	H	3.78478500	-1.03476300	-6.52461000
	C	2.21056700	-2.24602500	-5.66787800
	C	0.85639600	-2.57324200	-5.81257300
	H	0.42039500	-3.35888600	-5.20316800
	C	0.00000000	-1.75425900	-6.53674900
	H	-1.06929400	-1.92679000	-6.45711900
	C	2.89578000	-2.65342900	-4.41258500
	C	2.49837200	-3.77877000	-3.67565500
	H	1.91765300	-4.56216800	-4.15547400
	C	2.73408700	-3.85717300	-2.30773100
	H	2.30753600	-4.68612500	-1.74915700
	C	3.40273200	-2.82935600	-1.62656500
	C	3.96891400	-1.80838200	-2.39954700
	H	4.55745000	-1.03121900	-1.91969100
	C	3.71055000	-1.71586600	-3.76116700
	H	4.05483000	-0.83699400	-4.29849100
	C	3.36379800	-2.73714600	-0.14583500
	C	3.28906900	-1.47970300	0.47218600
	C	3.12717000	-1.35592400	1.84721800
	H	3.09037300	-0.36614600	2.29525900
	C	3.01578700	-2.48894500	2.66158200
	C	3.10368900	-3.74601700	2.04709400
	H	3.01997700	-4.64073900	2.65882100
	C	3.28238800	-3.86935800	0.67360900
	H	3.32065000	-0.58000400	-0.13538600
	H	3.35708500	-4.86086500	0.23458800
	H	-3.35708500	4.86086500	0.23458800
	H	-3.32065000	0.58000400	-0.13538600
	C	3.72953500	-3.12444500	6.32834400
	C	2.82458800	-2.35044000	6.98212100
	C	1.88267300	-1.55192800	6.25080400
	C	1.89648400	-1.56347000	4.80085700
	C	2.89095700	-2.39761700	4.14042500
	C	3.75626200	-3.13963200	4.90020700
	C	0.94684600	-0.78374000	6.92528500
	C	0.94798600	-0.80030000	4.12321800
	C	0.00000000	0.00000000	4.80422400
	C	0.00000000	0.00000000	6.24298100

	C	-0.94684600	0.78374000	6.92528500
	H	-0.93941200	0.79005300	8.01382800
	C	-1.88267300	1.55192800	6.25080400
	C	-1.89648400	1.56347000	4.80085700
	C	-0.94798600	0.80030000	4.12321800
	H	0.93941200	-0.79005300	8.01382800
	H	4.44530100	-3.72685000	6.87974200
	H	2.79198500	-2.31880000	8.06837500
	H	4.51024100	-3.74027200	4.39820900
	H	0.91273000	-0.82131700	3.03743300
	C	-3.75626200	3.13963200	4.90020700
	C	-3.72953500	3.12444500	6.32834400
	C	-2.82458800	2.35044000	6.98212100
	C	-2.89095700	2.39761700	4.14042500
	H	-4.51024100	3.74027200	4.39820900
	H	-4.44530100	3.72685000	6.87974200
	H	-2.79198500	2.31880000	8.06837500
	H	-0.91273000	0.82131700	3.03743300
	**[TET_6R1. GB]**<br>**E = -2827.462799**			
	C	2.89482200	3.95617200	-0.22776200
	C	2.27094600	4.77635100	-1.17612700
	H	2.87981800	5.28853000	-1.91698800
	C	0.88952100	4.93687500	-1.18903400
	C	0.07681700	4.26453600	-0.26806700
	C	0.70367500	3.44926800	0.68707100
	C	2.08656200	3.31250200	0.71502900
	H	2.55298800	2.70287700	1.48470800
	C	-1.40415700	4.31825800	-0.34012700
	C	-2.19347400	4.09246900	0.79406200
	H	-1.73169300	4.02750000	1.77534600
	C	-3.55733000	3.85282000	0.68005000
	H	-4.10212200	3.55288000	1.56921600
	C	-4.19302300	3.83831000	-0.56969500
	C	-3.43558800	4.24559400	-1.67864100
	H	-3.89816500	4.30923600	-2.65977100
	C	-2.06810000	4.46697900	-1.56746700
	H	-1.49755900	4.67496200	-2.46847300
	C	-5.44991400	3.05683700	-0.71652200
	C	-5.61193500	2.27306900	-1.86625500
	H	-5.02863900	2.48666800	-2.75654200
	C	-6.31505400	1.07575200	-1.81783600
	H	-6.24322700	0.40165600	-2.66632600
	C	-6.87742300	0.62140000	-0.61620500
	C	-6.97937500	1.55414000	0.42984900
	H	-7.50096800	1.28576800	1.34518700
	C	-6.27565300	2.75259000	0.37993400
	H	-6.27517100	3.39649800	1.25533900
	C	-6.82200900	-0.84593000	-0.37326400
	C	-6.87008600	-1.78431800	-1.41794600
	H	-7.42192500	-1.55532800	-2.32656700
	C	-6.08914200	-2.93372800	-1.37005000
	H	-6.06211200	-3.58816500	-2.23776800
	C	-5.23866400	-3.17836400	-0.27736200
	C	-5.42743200	-2.39756900	0.86909700
	H	-4.80280000	-2.54141500	1.74440000
	C	-6.21155400	-1.25172600	0.82125600
	H	-6.16520000	-0.56972200	1.66660500

	C	-3.96172800	-3.92756300	-0.40638500
	C	-3.22575200	-4.32521400	0.71875100
	H	-3.69705400	-4.34974000	1.69654900
	C	-1.86213400	-4.57250000	0.62845800
	H	-1.30627500	-4.75693200	1.54377000
	C	-1.17970100	-4.45536800	-0.59094300
	C	-1.94628700	-4.22486700	-1.74006100
	H	-1.46449600	-4.18605100	-2.71357100
	C	-3.30738900	-3.95859600	-1.64756200
	H	-3.84435700	-3.67038800	-2.54676200
	C	0.30152700	-4.37563500	-0.61247700
	C	0.94601700	-3.51061800	-1.50940500
	C	2.31179500	-3.26204100	-1.42326800
	H	2.78062200	-2.58789500	-2.13599700
	C	3.08866600	-3.86609100	-0.42745500
	C	2.45166300	-4.75614200	0.44891100
	H	3.03664600	-5.24235800	1.22555600
	C	1.08887800	-5.01366300	0.35382300
	H	0.36270500	-2.98000100	-2.25699400
	H	0.63067200	-5.70877800	1.05228300
	H	0.44031300	5.59567000	-1.92752300
	H	0.10288100	2.88286400	1.39454900
	C	6.77121500	-4.72133100	-0.20061300
	C	7.39073800	-3.51367800	-0.15327800
	C	6.62715800	-2.29880200	-0.18116400
	C	5.18038700	-2.35257900	-0.26582600
	C	4.55901900	-3.66779200	-0.32915900
	C	5.34721400	-4.78748200	-0.29075200
	C	7.26819300	-1.07269100	-0.11205900
	C	4.46977700	-1.15335900	-0.25633600
	C	5.11832500	0.10447500	-0.20052300
	C	6.55552700	0.13862200	-0.12496300
	C	7.20988100	1.38156100	-0.07403500
	H	8.29740300	1.40240800	-0.03081300
	C	6.51017000	2.57730100	-0.09206900
	C	5.06216700	2.56227100	-0.15928900
	C	4.41183000	1.33086600	-0.22289000
	H	8.35424400	-1.04379800	-0.04370500
	H	7.34647100	-5.64206100	-0.18242200
	H	8.47364100	-3.43878800	-0.09219400
	H	4.87258700	-5.76287900	-0.35966600
	H	3.38236500	-1.16908300	-0.28354000
	C	5.10794400	5.00283100	-0.18193300
	C	6.53548400	5.00427400	-0.12267300
	C	7.21333900	3.82830100	-0.06972000
	C	4.37577200	3.84576200	-0.19069400
	H	4.58220900	5.95417900	-0.18869900
	H	7.06598900	5.95164700	-0.10680500
	H	8.29891100	3.80700000	-0.01447200
	H	3.32898500	1.29668600	-0.31487900
	P	-2.59244200	0.12785800	1.78165700
	O	-2.96648100	-1.14400300	2.42957400
	O	-1.03735900	0.49445200	1.82898700
	C	-3.13451900	0.39013800	0.09451400
	H	-4.22209900	0.50996100	0.07200600
	H	-2.65679500	1.28275000	-0.32096500
	H	-2.86576800	-0.49349100	-0.49353500
	F	-3.18996200	1.39778200	2.52842900
	C	-0.05265500	-0.53081400	2.14690000
	H	-0.43136600	-1.09412900	3.00604100

C	1.22520300	0.20123700	2.50244100
H	1.98472400	-0.51437600	2.83241100
H	1.60754700	0.72362200	1.61971100
H	1.05532700	0.92962000	3.30080600
C	0.12186200	-1.44549600	0.94969800
H	-0.80448500	-1.98650500	0.72477400
H	0.41610900	-0.85946000	0.07062700
H	0.90401700	-2.18877300	1.14416500

[TET_6R1. GF]
E = -2944.157879

C	3.23945500	-3.73483500	-0.80327800
C	2.65396800	-4.69904000	0.02983100
H	3.27967100	-5.23435900	0.73965200
C	1.28831800	-4.95755400	-0.01095100
C	0.44861500	-4.24805000	-0.87743900
C	1.03965600	-3.30336800	-1.72972600
C	2.40805900	-3.05842900	-1.70327600
H	2.83441600	-2.32953000	-2.38740500
C	-1.02866500	-4.37261700	-0.83064100
C	-1.81134100	-4.12292100	-1.96461800
H	-1.33596400	-4.01656000	-2.93609400
C	-3.18231600	-3.92710900	-1.85679900
H	-3.73828500	-3.62144500	-2.73855900
C	-3.83013000	-3.98570500	-0.61374600
C	-3.07206800	-4.40418500	0.48936700
H	-3.54381600	-4.50722400	1.46264600
C	-1.69793700	-4.58605200	0.38322100
H	-1.12844500	-4.81164000	1.28085400
C	-5.13515100	-3.29218900	-0.45730700
C	-5.32085000	-2.50665200	0.68582300
H	-4.67694700	-2.62210200	1.55180100
C	-6.13881900	-1.38366700	0.64697400
H	-6.08333100	-0.69446800	1.48551400
C	-6.79103600	-1.01270300	-0.53613000
C	-6.83384200	-1.96123900	-1.57204800
H	-7.41345900	-1.75656200	-2.46924700
C	-6.01560500	-3.08445900	-1.53374200
H	-5.98348700	-3.74449200	-2.39720600
C	-6.91050500	0.44812000	-0.79183900
C	-7.06161900	1.38996600	0.23984000
H	-7.56211100	1.10600700	1.16230600
C	-6.43258300	2.62851500	0.16453100
H	-6.46848500	3.28668000	1.02856700
C	-5.62773600	2.96195500	-0.93964800
C	-5.74244200	2.14714500	-2.07348700
H	-5.17079800	2.37641000	-2.96735000
C	-6.37609000	0.91282600	-2.00222500
H	-6.26720300	0.22802300	-2.83811200
C	-4.40834800	3.80417200	-0.80389200
C	-3.65706600	4.21703300	-1.91586700
H	-4.11627600	4.25086300	-2.90013900
C	-2.29479900	4.47199900	-1.80240800
H	-1.72006800	4.67903300	-2.70166400
C	-1.63673100	4.34858700	-0.56968500
C	-2.42561200	4.13319500	0.56554700
H	-1.96318700	4.09222500	1.54796000
C	-3.78281500	3.86245800	0.44930700
H	-4.32425100	3.56434200	1.34120200

	C	-0.15776100	4.29485000	-0.48096700
	C	0.44620700	3.39403600	0.40964400
	C	1.82857900	3.26930700	0.48140700
	H	2.26963400	2.58265500	1.20024900
	C	2.66161300	4.02733200	-0.35089800
	C	2.05685900	4.91582000	-1.25071200
	H	2.68510500	5.50656900	-1.91238700
	C	0.67411600	5.05336500	-1.31174200
	H	-0.17858400	2.76016300	1.03525400
	H	0.23840800	5.76651700	-2.00704800
	H	0.86884300	-5.71104200	0.65111000
	H	0.41384600	-2.72044600	-2.39958300
	C	6.23379800	5.24311100	-0.00459200
	C	6.97365800	4.10393800	0.00081600
	C	6.34139800	2.82162000	-0.12642100
	C	4.89919100	2.73779500	-0.25201900
	C	4.14373500	3.98175800	-0.24273600
	C	4.81206000	5.17165000	-0.12667700
	C	7.10445800	1.66457900	-0.15311300
	C	4.31847200	1.48092300	-0.40247800
	C	5.08917700	0.29442600	-0.41271600
	C	6.51923700	0.39357500	-0.29480200
	C	7.28799400	-0.78319900	-0.33305400
	H	8.37027100	-0.70888100	-0.24203600
	C	6.70629700	-2.03287800	-0.48361000
	C	5.26523600	-2.15042200	-0.59244900
	C	4.50168300	-0.98558000	-0.54102300
	H	8.18739700	1.74043400	-0.07190600
	H	6.70956000	6.21416500	0.09489600
	H	8.05593200	4.13690900	0.09873000
	H	4.23799700	6.09402000	-0.09729500
	H	3.24311900	1.39219900	-0.53450400
	C	5.54196800	-4.57020200	-0.74551000
	C	6.96086500	-4.44178300	-0.64315200
	C	7.52404000	-3.21201700	-0.51668500
	C	4.70305700	-3.48781300	-0.72435300
	H	5.11353900	-5.56192700	-0.86512100
	H	7.57849000	-5.33438800	-0.67248600
	H	8.60188300	-3.09274300	-0.43844300
	H	3.41766800	-1.04033900	-0.59232400
	P	-2.77866400	0.24504600	1.81849300
	O	-3.50537700	-0.77662100	2.59637100
	O	-1.18825800	0.15729700	1.81627100
	F	-3.01118600	1.69404000	2.45284000
	C	-3.19998100	0.47390200	0.09597500
	H	-2.55290100	1.23094600	-0.35732200
	H	-3.09131500	-0.48484000	-0.42072100
	H	-4.24401000	0.79644500	0.02562100
	C	0.44399800	-2.31011200	2.13475800
	C	1.83328400	-1.67562900	2.05464100
	C	1.73551000	-0.16326600	1.83730100
	C	0.89568400	0.48855500	2.94087600
	C	-0.48278700	-0.15134200	3.05768500
	C	-0.40267600	-1.66454800	3.23305900
	H	2.73809800	0.28297800	1.81858500
	H	2.37740700	-1.86917400	2.99151200
	H	2.41307500	-2.14281300	1.25164400
	H	-0.06348100	-2.20171800	1.16630900
	H	0.53289400	-3.38674700	2.32118600
	H	1.39997900	0.36086100	3.90856300

	H	0.78170400	1.56701000	2.77977200
	H	-1.04146200	0.30291600	3.88509900
	H	0.05370700	-1.85636700	4.21458400
	H	-1.41606400	-2.07876600	3.25651500
	H	1.27339700	0.04104900	0.85985900
